# Microfluidic device with brain extracellular matrix promotes structural and functional maturation of human brain organoids

**DOI:** 10.1038/s41467-021-24775-5

**Published:** 2021-08-05

**Authors:** Ann-Na Cho, Yoonhee Jin, Yeonjoo An, Jin Kim, Yi Sun Choi, Jung Seung Lee, Junghoon Kim, Won-Young Choi, Dong-Jun Koo, Weonjin Yu, Gyeong-Eon Chang, Dong-Yoon Kim, Sung-Hyun Jo, Jihun Kim, Sung-Yon Kim, Yun-Gon Kim, Ju Young Kim, Nakwon Choi, Eunji Cheong, Young-Joon Kim, Hyunsoo Shawn Je, Hoon-Chul Kang, Seung-Woo Cho

**Affiliations:** 1 Department of Biotechnology, Yonsei University, Seoul, Republic of Korea; 2 Department of Biochemistry, Yonsei University, Seoul, Republic of Korea; 3 Institute of Molecular Biology and Genetics, Seoul National University, Seoul, Republic of Korea; 4 Signature Program in Neuroscience and Behavioral Disorders, Duke-NUS Medical School, Singapore, Singapore; 5 Department of Chemical Engineering, Soongsil University, Seoul, Republic of Korea; 6 Division of Pediatric Neurology, Department of Pediatrics, Severance Children’s Hospital, Yonsei University College of Medicine, Seoul, Republic of Korea; 7 Department of Chemistry, Seoul National University, Seoul, Republic of Korea; 8 Department of Advanced Materials Engineering, Kangwon National University, Samcheok, Republic of Korea; 9 Brain Science Institute, Korea Institute of Science and Technology (KIST), Seoul, Republic of Korea; 10 Center for Nanomedicine, Institute for Basic science (IBS), Seoul, Republic of Korea; 11 Graduate Program of Nano Biomedical Engineering (NanoBME), Advanced Science Institute, Yonsei University, Seoul, Republic of Korea

**Keywords:** Microfluidics, Biomaterials, Stem-cell biotechnology, Tissue engineering, Neurogenesis

## Abstract

Brain organoids derived from human pluripotent stem cells provide a highly valuable in vitro model to recapitulate human brain development and neurological diseases. However, the current systems for brain organoid culture require further improvement for the reliable production of high-quality organoids. Here, we demonstrate two engineering elements to improve human brain organoid culture, (1) a human brain extracellular matrix to provide brain-specific cues and (2) a microfluidic device with periodic flow to improve the survival and reduce the variability of organoids. A three-dimensional culture modified with brain extracellular matrix significantly enhanced neurogenesis in developing brain organoids from human induced pluripotent stem cells. Cortical layer development, volumetric augmentation, and electrophysiological function of human brain organoids were further improved in a reproducible manner by dynamic culture in microfluidic chamber devices. Our engineering concept of reconstituting brain-mimetic microenvironments facilitates the development of a reliable culture platform for brain organoids, enabling effective modeling and drug development for human brain diseases.

## Abstract (web summary)

Brain organoids derived from human pluripotent stem cells can model human brain development and disease, though current culture systems fail to ensure reliable production of high-quality organoids. Here the authors combine human brain extracellular matrix and culture in a microfluidic device to promote structural and functional maturation of human brain organoids.

## Introduction

Recent breakthroughs in brain organoid technology have enabled the development of a new in vitro model that promotes significant advancements in the study of nervous system development and diseases^1^. Sasai and co-workers proposed the idea that differentiated pluripotent cells can form multi-layered organized structures that recapitulate embryonic development when grown in three-dimensional (3D) culture^2^. Lancaster et al. developed a 3D culture model termed “cerebral organoids” that recapitulate many key features of the human brain in vivo and develop various distinct and interdependent brain regions^3^. The generation of cerebral organoids depends on the intrinsic ability of pluripotent stem cells (PSCs) to spontaneously self-organize upon precisely timed manipulation of culture conditions even in the absence of external patterning factors^3–5^. Cerebral organoids representing the whole brain have significant advantages over brain region-specific^2,6–12^ or extensively patterned organoids^13–17^ due to their ability to generate a diverse range of brain cells and recapitulate the major events in overall brain development^18^. Despite the potential of cerebral organoid technology, there are several challenges. Due to the lack of instructive signals during the generation of human cerebral organoids, they recapitulate only some of the earliest stages of human embryonic brain development^5^ and are not able to mimic the later stages of neurogenesis until extended cultivation for 6–9 months^19^. Another critical limitation is the extensive cell death in the developing organoids at later stages due to diffusional limitations in oxygen and nutrient transfer^4,8^.

Several engineering strategies with biomaterials, bioreactors, devices, and genetic modification have been demonstrated to overcome such limitations of current brain organoid culture. For example, synthetic polymer microfilaments enhanced neuroectoderm formation and cortical development by facilitating guided self-organization via neuroepithelium elongation^20^. In other study, miniaturized spinning bioreactors were tested for improving the dynamic culture of brain organoids, which generated more robust disease models with Zika virus infection^8^. To increase the oxygen supply, air–liquid interface culture was adapted for cerebral organoids, resulting in improved survival and morphology with extensive axonal outgrowths^21^. Organ-on-a-chip systems were also employed to improve the oxygen supply to the brain organoids^22,23^. The vascularization of brain organoids by grafting human brain organoids into the mouse brain or gene editing of vascular transcription factor resulted in progressive neurogenesis with improved neuronal survival^24^. Despite these recent technical improvements, certain progenitor cells still showed low abundance, and the cytoarchitecture of the basal zones and cortical layers was not complete^3,4^. Moreover, the current protocols based on spontaneous self-organization have exhibited a significant batch-to-batch variation, which in turn results in poor reproducibility. Therefore, cerebral organoids still need to be improved further for neuronal development, structural maturation, and better electrophysiological functionality, as well as for ensuring consistent organoid quality.

Here, we propose a strategy to engineer human PSC-derived cerebral organoids by reconstituting a 3D brain-mimetic microenvironment with a decellularized human brain tissue-derived brain extracellular matrix (BEM) and dynamic microfluidic systems. BEM can recreate brain-mimetic niches necessary to guide neural and glial differentiation for brain organogenesis, which would likely be deficient in the non-neuronal matrix (e.g. Matrigel)^25^. The application of microfluidic devices capable of achieving a gravity-driven flow that mimics a fluid flow existing in the cerebrospinal and interstitial spaces can facilitate the oxygen supply and nutrient/waste exchange, leading to a significant reduction of cell death throughout the structure of organoids. Thus, we reason that providing brain-specific extracellular matrix (ECM) cues together with improved nutrient and oxygen exchange will support cell expansion as well as neuronal differentiation and functional maturation, thereby recapitulating prominent features of human embryonic cortical development in a much precise and reproducible manner.

## Results

### Characterization of a human brain-mimicking 3D hydrogel matrix

**Fig. 1:**
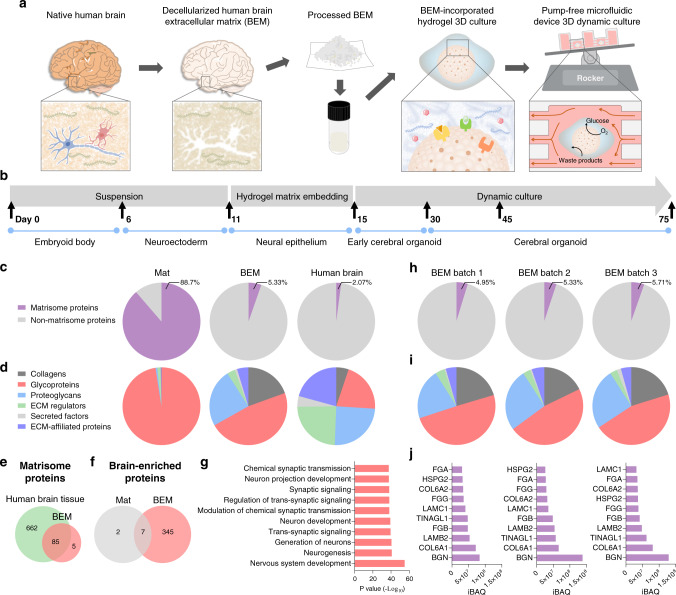
Characterization of decellularized human brain-derived extracellular matrix (BEM). **a** Schematic illustration of the cerebral organoid culture system with a combination of 3D BEM hydrogel culture and the microfluidic device. **b** Schematic of the culture protocol for generating cerebral organoids from human induced pluripotent stem cells (iPSCs) using a brain-mimetic culture system. The developmental stage of organoids is shown at the bottom, the timeline for each developmental stage is shown in the middle, and the culture condition is shown at the top. **c**–**j** Proteomic analysis for the identification of extracellular matrix (ECM) components in human BEM. The percentages of (**c**) matrisome proteins out of total proteins, and (**d**) the subtypes of matrisome proteins identified in BEM, Matrigel (Mat), and human brain tissue (*n* = 1 for Mat, *n* = 3 for BEM). **e** Total numbers of matrisome proteins detected in the human brain tissue and BEM. **f** The number of the brain-enriched proteins by at least 4-fold compared to other organs. **g** The top 10 biological process terms ordered by *p* value after gene ontology enrichment analysis of brain-enriched proteins exclusively present in BEM, which are not detected in Mat. The compositions of (**h**) matrisome proteins relative to total proteins and (**i**) the subtypes of matrisome proteins in three batches of BEM (biological replicates = 3). **j** Most abundant matrisome proteins found in each batch of BEM. Source data are provided as a Source Data file.

A bioengineering platform to improve human brain organoid culture was set up with human brain-mimicking 3D hydrogel and a microfluidic system. Human cerebral organoids were generated from human induced pluripotent stem cells (iPSCs) as described in Lancaster’s protocol (Supplementary Fig. 1)^4^. When embryoid bodies (EBs) were induced to develop into a neuroepithelial lineage at day 11, they were embedded in a 3D matrix supplemented with human BEM (0.4 mg/ml) (Fig. 1a). Because Matrigel, a common and essential component of the organoid culture, is refractory to the tissue-specific ECM cues that are required by different tissue types^26,27^, modification of Matrigel-based organoid culture by supplying human BEM would provide enhanced cell growth and more favorable interactions at an early stage of neurogenesis. After four days of culture in BEM-incorporated gel, the organoids were transferred into the microfluidic device under dynamic conditions (Fig. 1a, b). Our microfluidic platform can allow for independent control of the cerebral organoids in much smaller medium volume and precisely controlled medium flow with low fluid shear stress (Supplementary Fig. 2), compared to typical bulk scale bioreactors (e.g. spinner flasks, orbital shakers) which require larger volumes and evoke cell damage due to the high shear stress. With the precisely controlled fluid flow, the effective exchange of oxygen, nutrients, and bioactive molecules in the medium leads to the robust expansion and reduced cell apoptosis at an early stage of organoid development. Consequently, more complex structures with elongated cortical layers would be evident in cerebral organoids.

BEM enriched with brain ECM components was prepared by the decellularization of human brain tissue and subsequent tissue processing. Human brain samples were pooled before decellularization (Supplementary Table 1). Cellular components in brain tissue were removed by treatment with a non-ionic detergent (Supplementary Fig. 3a, b). The decellularization process did not have a significant effect on the glycosaminoglycan (GAG) content (Supplementary Fig. 3c). Histological analysis of Hematoxylin and Eosin (H&E) and Masson’s Trichrome (MT) stained samples suggested that most of the cells were removed, while collagen, one of the major ECM components, remained intact after decellularization (Supplementary Fig. 3d). Proteomic analysis of BEM with mass spectrometry revealed that BEM contains the enrichment of brain-specific ECM components, including various collagen subtypes, proteoglycans (e.g. heparan sulfate, neurocan, versican), and glycoproteins (e.g. laminin, tenascin) (Supplementary Table 2). Collagens, proteoglycans (e.g. neurocan, versican), and glycoproteins (e.g. laminin, tenascin)—detected in BEM—can affect various processes involved in neurogenesis, such as neuronal polarization and migration, neurite outgrowth, axon guidance, and synapse development^28–31^. Thus, BEM can provide a variety of biological components that influence the development and function of brain organoids. Rheological analysis of BEM and Matrigel (Mat) hydrogels identified a higher level of storage (elastic) modulus than loss (viscous) modulus in all tested frequency ranges (0.1–10 Hz), indicating the formation of a stable internal network in both hydrogel systems (Supplementary Fig. 4a). The average storage modulus of Mat and BEM hydrogels at 1 Hz was 115.56 ± 5.55 Pa and 126.17 ± 13.52 Pa, respectively (Supplementary Fig. 4b), indicating that there is no significant difference in terms of mechanical properties between these two hydrogels. These data suggest that the positive effects of BEM hydrogel on the development of brain organoids are more likely to be due to the biochemical signals rather than due to mechanical cues.

The ECM profiles in BEM were compared with those in Mat and human brain tissue. All identified proteins were categorized as either ECM or its associated proteins, commonly referred as ‘Matrisome’^32–34^. We checked ‘Human Protein Atlas’ to examine whether BEM has comparable ECM profiles to the normal human brain (Fig. 1c–f). It appears that BEM and human brain tissue contain a similar percentage of matrisome proteins out of total proteins (2–5%), whereas majority of proteins in Mat are matrisomes (~90%) (Fig. 1c). Significant percentages of collagens, glycoproteins, and proteoglycans are contained in BEM and human brain tissue, but Mat primarily consists of glycoproteins (Fig. 1d). We identified 90 ECM proteins in BEM, whereas 747 are found in human brain tissue in Human Protein Atlas (Supplementary Table 3). We found that 85 out of 90 ECM proteins (94%) in BEM are expressed in the native brain tissue (Fig. 1e). The lists of identified proteins in Mat and BEM were also compared with the list of proteins that are known to show elevated expression in the human brain tissue at least four-fold higher compared to other tissue types^35^. Only 9 brain tissue-enriched proteins were found in Mat, whereas 352 proteins were identified in BEM (Fig. 1f). A gene ontology biological process (GOBP) analysis indicates that the brain tissue-enriched proteins only identified in BEM are involved in nervous system development and neurogenesis (Fig. 1g). Overall, our proteomics data revealed that the portion and types of matrisome proteins in BEM are much closer to those of human brain tissue than Mat.

The variability of BEM batches arising from the isolation of BEM from different sources should be addressed to standardize the BEM for brain organoid culture. The proteomic analysis of different batches of BEM showed that portion and compositions of matrisomal contents are quite similar in three batches of BEM (batch 1, 2, 3) (Fig. 1h, i). Additionally, the top 10 proteins with the highest intensity-based absolute quantification (iBAQ) values completely overlapped in all three batches (Fig. 1j). Although each batch of BEM was pooled from 2–3 patient samples with different age and gender, batch-to-batch variability of BEM was not significant in our study. It was previously reported that brain ECM composition is altered in cortical dysplasia and temporal lobe epilepsy^36^. For example, upregulation of neurocan and tenascin-C was observed in a murine model of temporal lobe epilepsy, while major ECMs, such as glycoproteins, laminin, and fibronectin that are implicated in tissue remodeling, showed no significant change^37^. Although the expression of some of the ECM proteins might be altered in BEM derived from epilepsy patients, apparent proteins that elicit pathological signals (e.g. pro-inflammatory cytokines, matrix metalloproteinase-9)^37,38^ were not identified in our BEM samples. The GOBP analysis of the total proteins as well as non-matrisome proteins also suggested that BEM does not contain proteins that are involved in inflammation (Supplementary Table 4).

### BEM increases the neuronal population and enhances neurogenesis in the brain organoid

**Fig. 2:**
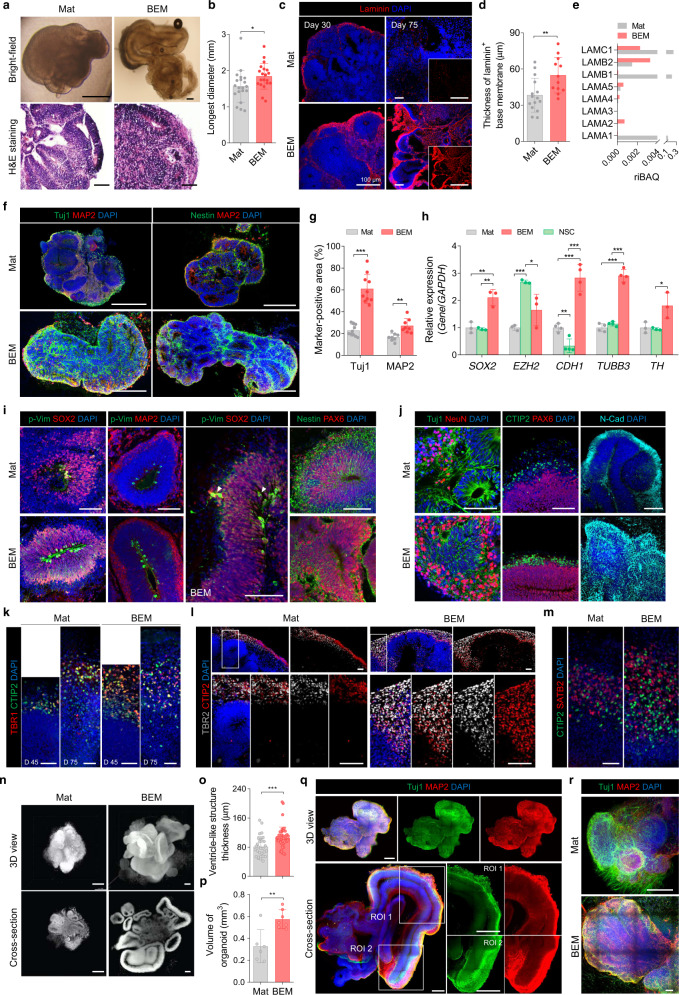
BEM improves neurogenesis and organization of cortical layers in cerebral organoids. **a** Bright-field images and hematoxylin and eosin (H&E) staining of Mat- and BEM-embedded brain organoids at day 30 (scale bars = 500 μm for bright-field and 100 μm for H&E staining images, independent replicates = 3). **b** Quantification of the longest diameter of Mat and BEM organoids based on the bright-field images of whole organoids (*n* = 20, Mat versus BEM *p* = 0.0349, independent replicates = 3). **c** Laminin staining of Mat and BEM organoids at 30 and 75 days (scale bars = 100 μm), and (**d**) quantification of the thickness of laminin^+^ basement membrane covering the outer surface of the organoids at day 30 (*n* = 15 for Mat and *n* = 12 for BEM, Mat versus BEM *p* = 0.0066, independent replicates = 5). **e** Comparison of relative intensity-based absolute quantification (riBAQ) values of laminin subtypes identified in Mat and BEM. **f** Immunohistochemical staining of neural progenitor marker (Nestin) and neuronal markers (Tuj1 and MAP2) at 30 days of culture (scale bars = 500 μm, independent replicates = 1–4). **g** Image-based quantification of the Tuj1- and MAP2-positive area in the Mat and BEM organoids at day 30 (*n* = 10 for Tuj1 and *n* = 15 for MAP2, Mat versus BEM *p* < 0.0001 for Tuj1; Mat versus BEM *p* = 0.0012 for MAP2, independent replicates = 2–7). **h** The quantitative real-time polymerase chain reaction (qPCR) analysis to compare gene expression between Mat, neural stem cells (NSCs), and BEM organoids at day 30 (*n* = 3 for *SOX2*, *EZH2*, and *TH* and *n* = 4 for *CDH1* and *TUBB3*, 10–15 brain organoids collected as one sample batch, Mat versus BEM *p* = 0.0061, NSC versus BEM *p* = 0.0027 for *SOX2*; Mat versus NSC *p* < 0.0001, NSC versus BEM *p* = 0.0371 for *EZH2*; Mat versus BEM *p* = 0.0004, Mat versus NSC *p* = 0.0039, NSC versus BEM *p* = 0.0001 for *CDH1*; Mat versus BEM *p* < 0.0001, NSC versus BEM *p* < 0.0001 for *TUBB3*; NSC versus BEM *p* = 0.0346 for *TH*, independent replicates = 3). **i** Immunohistochemical staining for mitotic radial glia marker phosphorylated vimentin (p-Vim), SOX2, Nestin, and PAX6, and neuron marker MAP2 in Mat and BEM organoids at day 30 (scale bars = 100 μm, independent replicates = 6). Note that some p-Vim^+^/SOX2^+^ cells are located above the apical domain (white arrowheads). **j** Immunohistochemical staining for Tuj1 and NeuN at day 30, CTIP2 and PAX6, and N-cadherin (N-Cad) at day 45 in Mat and BEM organoids (scale bars = 100 μm, independent replicates = 2–5). **k** Immunohistochemical stained images for deep-layer neuron markers TBR1 and CTIP2 at 45 and 75 days (scale bars = 50 μm, independent replicate = 1). Immunostaining images for (**l**) intermediate progenitor marker TBR2 and CTIP2 and (**m**) superficial-layer neuron marker SATB2 and CTIP2 in 75-day organoids (scale bars = 50 μm, independent replicate = 1). **n** Nucleus-stained (Cyto16) light-sheet microscopic images of Mat and BEM organoids at day 75 (scale bars = 500 μm, independent replicates = 5). Quantification of (**o**) the thickness of ventricle-like structures (*n* = 30 for Mat and *n* = 45 for BEM, Mat versus BEM *p* = 0.0007) a*n*d (**p**) the total organoid volume (*n* = 6 for Mat and *n* = 9 for BEM, Mat versus BEM *p* = 0.0035) based on nucleus-stained (Cyto16) light-sheet microscopic images of Mat and BEM organoids at day 75 (independent replicates = 5). **q** 3D reconstituted images of 30-day BEM organoids obtained by tissue clearing and subsequent immunostaining for Tuj1 and MAP2 (scale bars = 1 mm, independent replicate = 1). **r** 3D images of Mat and BEM organoids stained for Tuj1 and MAP2 after tissue clearing at day 75 (scale bars = 200 μm, independent replicates = 3–5). Mat and BEM organoids were cultured in a dish on an orbital shaker. All data are expressed as mean ± standard deviation (SD). Statistical differences between the groups were determined with a two-sided *t*-test (*p < 0.05, **p < 0.01, ***p < 0.001). Source data are provided as a Source Data file.

The potential of BEM for promoting brain organoid formation and development of the neuronal population was investigated. Brain organoids grown in BEM hydrogel (BEM-incorporated Matrigel) were compared with those in Mat alone at 30 days of culture. BEM organoids were significantly larger (*p* < 0.05) than Mat organoids (1.56 ± 0.44 and 1.84 ± 0.35 mm in diameter for Mat and BEM organoids, respectively) (Fig. 2a, b). Some samples in the BEM group grew up to 4–5 mm by 30 days of culture (Fig. 2a, bright-field image). Immunostaining analysis revealed the presence of laminin-rich basement membranes at the outer border of the brain organoids in both the BEM and Mat groups (Fig. 2c, d), which is an important phenomenon in neocortical development in vivo. The formation of a laminin layer in BEM organoids was consistently relatively thicker than in Mat organoids. Although both BEM and Mat organoids initially formed a laminin-rich basement membrane in early neuroepithelium at day 30 (Fig. 2c), only BEM organoids maintained a thick laminin basement membrane at day 75 (Fig. 2c). Mat organoids showed only sparse and punctate signals in laminin staining (Fig. 2c), which indicates that they failed to maintain the basement membrane upon the generation and basal migration of neurons^3^. This observation suggests that BEM contributed to the maintenance of a laminin basement layer that could induce the organization of radially aligned neurons in the cortical layers of the organoids. In the proteomic analysis, BEM was found to contain more abundant subtypes of laminin (α2, α4, α5, and β2) than Mat except for α1, β1, and γ1, but Mat contains approximately 80 times higher relative intensity-based absolute quantification (riBAQ) values of total laminin subtypes (0.594 for Mat and 0.007 for BEM) (Fig. 2e). Given that Mat is much more enriched with laminins than BEM, the thicker laminin layer formation in the BEM organoids (Fig. 2c, d) is more likely to be derived intrinsically from the developing organoids rather than ectopically supplied from BEM.

To examine the generation of the neural population and neuronal differentiation, the expression of neural progenitor (NP) markers [Nestin, sex-determining region Y-box 2 (SOX2)] and neuronal markers [class III beta-tubulin (Tuj1), microtubule-associated protein 2 (MAP2)] was analyzed (Fig. 2f, g and Supplementary Fig. 5a, b). The areas positive for Tuj1 and MAP2 were more apparent in the BEM organoids, indicating that BEM increased the neuronal populations and enhanced neuronal differentiation in the organoids (Fig. 2f, g). In the BEM organoids, Tuj1-expressing neuronal cells were present throughout the whole constructs, whereas a layer of neurons expressing MAP2—a marker of mature neurons—was mainly located at the basal side of the ventricle-like structures in the peripheral regions of the organoids (Supplementary Fig. 5b, c). The apicobasal axis formation was more evidently observed in brain organoids grown in BEM hydrogel than in organoids produced in Mat (Fig. 2f and Supplementary Fig. 5d), suggesting that BEM organoids better mimicked a distribution of neuronal population showing apicobasal migration in vivo^6^. In Mat organoids, smaller ventricle-like structures were developed, and apicobasal axis polarity was less frequently observed (Fig. 2f). These collective observations demonstrated that the exposure of developing organoids to BEM resulted in the formation of highly dense ventricle-like structures with few vacant spaces, their integration into well-organized structures, and enhanced neuronal differentiation with radial polarity.

Compared to organoids cultured in Mat and neurospheres of human fetal brain-derived neural stem cells (NSCs), human brain organoids produced in the BEM hydrogel exhibited gene expression profiles that are indicative of enhanced neurogenesis (Fig. 2h). As analyzed by quantitative real-time polymerase chain reaction (qPCR) at 30 days of culture, BEM organoids displayed approximately two-fold upregulation of an NP marker, *SOX2*, compared to Mat organoids and NSCs. The highest expression of the Enhancer of zeste homolog 2 (*EZH2*) was found in NSCs, and the lowest expression was found in Mat organoids. *EZH2* involved in stem cell renewal and maintenance is highly expressed in NSCs or progenitor cells of the cortex, but its expression decreases in differentiated neurons^39–41^. The expression of *CDH1*—a co-factor that is required for the regulation of cortical neurogenesis and survival^42^—was noticeably higher in the BEM group than in other groups. BEM organoids displayed the highest *TUBB3* (Tuj1) expression, and Mat organoids and NSCs showed similar levels of *TUBB3* expression. The expression of tyrosine hydroxylase (*TH*), a dopaminergic neuron marker, was also the highest in the BEM group. Immunostaining for a forebrain marker FOXG1 showed that BEM organoids at 75 days of culture had larger FOXG1^+^ brain lobules than those in Mat organoids, suggesting enhanced forebrain identity (Supplementary Fig. 5e). BEM organoids are likely to have more NPs than Mat organoids, while undergoing enhanced neurogenesis at the same time, as indicated by upregulated expression of both progenitor marker (*SOX2*) and neuronal marker (*TUBB3*) (Fig. 2h). This might be because proliferation and differentiation of NPs simultaneously occur at early phase of brain organoid development (~day 30). The rates of proliferation and neurogenesis continuously change during brain development, and their balance is thought to determine the brain size^43^. Thus, enhanced proliferation of NPs in BEM organoids may contribute to the increased size of brain organoids (Fig. 2b).

Surprisingly, an increase in BEM dose did not improve organoid development (Supplementary Fig. 6). We determined 0.4 mg/ml BEM concentration as an optimal dose for human brain organoid culture. Microscopic observation and immunohistochemical staining for Tuj1 and SOX2 indicated that higher BEM concentrations (1 and 2 mg/ml) than 0.4 mg/ml BEM did not enhance neuronal marker expression and neuroepithelial outgrowth in brain organoids (Supplementary Fig. 6a). qPCR analysis at 30 days of culture revealed that the expression of neuronal differentiation markers (*Nestin*, *PAX6*, *TUBB3, MAP2*) was generally higher in brain organoids grown in 0.4 mg/ml BEM hydrogel than in hydrogel with other BEM concentrations (Supplementary Fig. 6b). These data suggest that an optimal concentration of BEM is required for the development of brain organoids with sophisticated neurogenesis and neuroectoderm-like morphology.

### BEM increases the radial glial cell population and promotes cortical layer development in brain organoids

Brain organoids grown in BEM hydrogel contain a larger radial glial cell (RGC) population along the ventricular zone (VZ) (Fig. 2i). RGCs represent a major progenitor pool during early development that can give rise to neurons and glia, and act as an axis to guide neuronal migration^44,45^. RGCs also play a key role in gyrification^46^. Immunostaining for radial glial markers phosphorylated vimentin (p-Vim), SOX2 and PAX6 at day 30 showed more densely packed cells in BEM organoids than in Mat organoids (Fig. 2i). The majority of p-Vim^+^ mitotic radial glial-like cells were located at the apical surface of the VZ in both Mat and BEM organoids. In BEM organoids, radial glial-like cells that extend basal cellular processes toward the outer surface were frequently observed, and some were located above the apical domain, suggesting basal (or outer) radial glia (Fig. 2i). Mat organoids had sparse populations of p-Vim^+^ RG-like cells (Fig. 2i). Furthermore, a larger number of PAX6^+^ radial glial progenitor populations were observed in BEM organoids than in Mat organoids (Fig. 2i). At day 45, BEM organoids displayed formation of more densely populated CTIP2^+^ deep-layer above PAX6^+^ VZ, compared to Mat organoids (Fig. 2j). This may be due to the presence of an organized layer of laminin-rich basement membrane in the BEM organoids (Fig. 2c), which supports cortical layer formation. Immunostaining of brain organoids cultured for 45 days showed that N-cadherin, which is involved in the radial migration of multipolar cells^47^ and maintaining the normal architecture of the neuroepithelial or RGCs^48^, was more highly expressed in BEM organoids than in Mat organoids (Fig. 2j).

To determine whether developing organoids in the BEM hydrogel could recapitulate the cortical spatial organization, the organoids were stained for cortical layer markers on days 45 and 75 in the culture. At day 45, the deep layer marked by TBR1^+^ (cortical layer VI) and CTIP2^+^ (cortical layer V) neurons started to express (Fig. 2k). A thicker layer of TBR1^+^ and CTIP2^+^ neurons was found in the BEM organoids than in the Mat organoids at both 45 and 75 days (Fig. 2k). Radial glia gave rise to intermediate progenitor cells (IPCs) that express TBR2 and move to the subventricular zone (SVZ)^44,45,49^. A well-organized layer containing a mixed population of TBR2^+^ IPCs and CTIP2^+^ neurons was reproducibly observed above VZ in the BEM organoids at day 75 (Fig. 2l). Similarly, a thicker layer containing CTIP2^+^ neurons and SATB2^+^ late-born neurons was observed in the BEM organoids at day 75 (Fig. 2m). At this stage, the BEM organoids contained different neuronal subtypes expressing SVZ, upper- and deep-layer markers that did not show distinguishably separate layers but given a distribution pattern of dorsal cortical subtype-specific neuronal populations that resembles in vivo regional sub-specification, BEM organoids could model the organization of the neocortex in vivo more closely than the Mat organoids. Bright-field images revealed more elongated neuroepithelium structures in the BEM organoids, which was rarely observed in the Mat organoids (Supplementary Fig. 7a). The BEM organoids displayed a more complex 3D architecture with larger cortical layers than the Mat organoids (Fig. 2n and Supplementary Video 1). The brain organoid culture in BEM hydrogel induced the significant enlargement of individual ventricle-like structure thickness as well as the overall volume of the organoid (Fig. 2o, p). The organization of cortical regions in the BEM organoids was further confirmed by tissue clearing using the CUBIC protocol^50^ and subsequent staining for neuronal markers (Tuj1, MAP2) (Fig. 2q, r, Supplementary Fig. 7b, c, and Supplementary Video 2). The elongated neuroepithelial structures were observed, while maintaining a high density of cells, and neurons were strongly polarized along their apicobasal axis (Fig. 2q and Supplementary Fig. 7b, c). BEM organoids contained much larger lobes than the Mat organoids (Fig. 2r).

### BEM elicits the maturation of cerebral organoids at molecular and functional levels

**Fig. 3:**
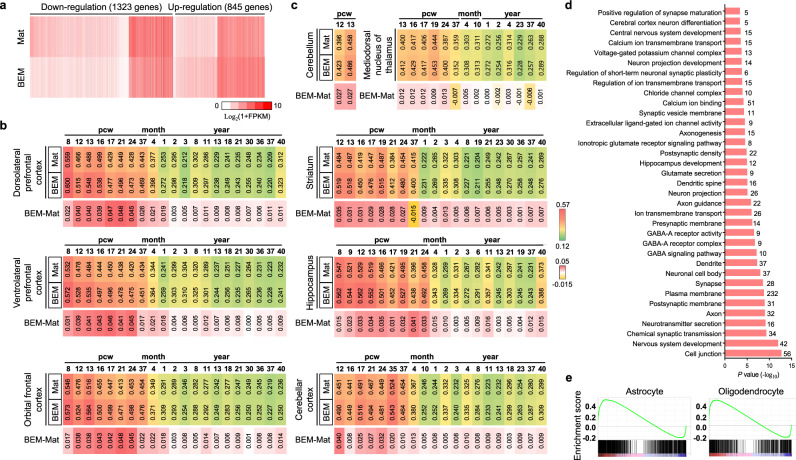
The genome-wide transcriptome analysis of 75-day brain organoids. **a** Heatmap showing the expression of differentially expressed genes (DEG) between Mat and BEM organoids (*n* = 3 per group, independent replicate = 1). **b**, **c** Heatmaps of Pearson’s correlation analysis of RNA-sequencing datasets of the Mat and BEM organoids for comparison with published transcriptome datasets of (**b**) three different human frontal regions across different stages and (**c**) five different regions of the brain at fetal and postnatal stages. Values in the heatmaps of Mat and BEM groups dictate Pearson’s correlation coefficients (PCC). Values in the heatmaps of BEM-Mat indicate the differences in PCC between BEM and Mat groups. **d** The 35 enriched gene ontology (GO) terms of upregulated genes in BEM organoids versus Mat organoids (shown in terms of *p*-values). Numbers on the right-hand side of the bar indicate the number of DEGs within the GO terms. **e** The gene set enrichment analysis (GSEA) of the astrocyte and oligodendrocyte probe sets in the BEM group versus the Mat group. Mat and BEM organoids were cultured in a dish on an orbital shaker. Source data are provided as a Source Data file.

To further examine the effects of BEM on brain organoid development and to compare the developmental level of BEM organoids with the human brain tissue, an RNA-sequencing analysis of global transcriptomes was conducted with three batches of BEM and Mat organoids at 75 days of culture (Fig. 3). Compared to Mat organoids, 1323 genes were downregulated, and 845 genes were upregulated in BEM organoids (Fig. 3a). We compared organoid transcriptional profiles with datasets of brain-specific regions of fetal and adult human brains; three different parts of the human frontal brain and five regions of the brain, including the cerebellar cortex, hippocampus, cerebellum, striatum, and mediodorsal nucleus of the thalamus. A Pearson correlation analysis of three regions of the frontal cortex revealed that both the brain organoid groups generally correlated more closely with the fetal brains (Fig. 3b). In all three regions, BEM organoids showed stronger correlations with human brains than the Mat organoids. Similarly, gene expression patterns related to five other brain regions in the organoids were more strongly correlated with those in the fetal brains, where BEM organoids also exhibited higher correlation coefficient values with human brains than the Mat organoids (Fig. 3c). These findings suggest that the organoids generally resembled identities of various human fetal brain regions in the gestational weeks, and BEM organoids showed a higher correlation with the human brain than the Mat organoids. Gene ontology (GO) performed to assess the differentially expressed genes between BEM and Mat organoids supported the effectiveness of BEM for brain organoid maturation. The GO terms involved in nervous system development, neurotransmitter secretion, axon, and synapse were significantly upregulated in BEM organoids (Fig. 3d). These results confirm our earlier observations by immunostaining and qPCR that BEM promoted brain development and differentiation of neuronal populations. Gene set enrichment analysis (GSEA) revealed the significantly increased expression of gene sets for astrocyte and oligodendrocyte, both of which start to appear at a later stage during brain organogenesis, in BEM organoids compared to that in Mat organoids (Fig. 3e). Interestingly, differentially expressed genes in developing BEM organoids significantly overlapped with known risk genes related to schizophrenia, autism, epilepsy, and stroke (Supplementary Fig. 8). Therefore, brain organoids generated with BEM might be useful for disease modeling and disease mechanism studies during human brain development.

Importantly, BEM organoids exhibited improved electrophysiological functionality and neurotransmitter response over Mat organoids. The level of calcium channel activation was examined in response to glutamate-induced depolarization using a calcium influx indicator Fluo-4 AM at day 45 (Supplementary Fig. 9a–c). Upon exposure to glutamate, greater calcium influx changes were detected in BEM organoids than those in Mat organoids (Supplementary Fig. 9a, b). The quantification of the influx intensity indicated more noticeable changes in the cytoplasmic calcium influx in individual cell populations of BEM organoids (Supplementary Fig. 9c). The percentage of glutamate-responsive cells was also much higher in BEM organoids (Supplementary Fig. 9c), indicating the existence of electrically more functional neural cell population in the cerebral organoids grown in the BEM hydrogel. Immunohistochemical analysis for vesicular glutamate transporter 1 (VGLUT1), an excitatory neuronal marker, (Supplementary Fig. 9d) indicated that BEM organoids at 45 days contained VGLUT1^+^ mature excitatory neurons that are known to emerge during the second trimester of the neonatal brain^51^. In contrast, the Mat organoids contained only a few VGLUT1^+^ cells at this stage. To assess the electrophysiological properties of cells in brain organoids, a whole-cell patch analysis was performed (Supplementary Fig. 9e, f). A voltage-dependent tetrodotoxin (TTX)-sensitive sodium current was detected and action potential (AP) was also evoked with current injections. A physiologically functional recording was barely observed in the Mat organoids at the same time point. Specific cell populations in BEM organoids responded to γ-aminobutyric acid (GABA) treatment (Supplementary Fig. 9g)^8^. Glutamate decarboxylase 1 (GAD1)^+^ GABAergic neurons that produce GABA for inhibitory neuronal activity were detected in BEM organoids (Supplementary Fig. 9h). Therefore, our findings demonstrate that BEM organoids mimic some features of the later developmental stages of the human brain with more functional neuronal properties than the control organoids grown in Matrigel. Overall, the exposure of organoids with neuroectoderm identity to the complex networks of BEM at an early developmental stage triggered NP expansion, cortical layer organization, and enhanced neurogenesis, leading to the structural maturation and functional improvement of organoids.

### The tissue-specific effects of BEM on brain organoid generation

**Fig. 4:**
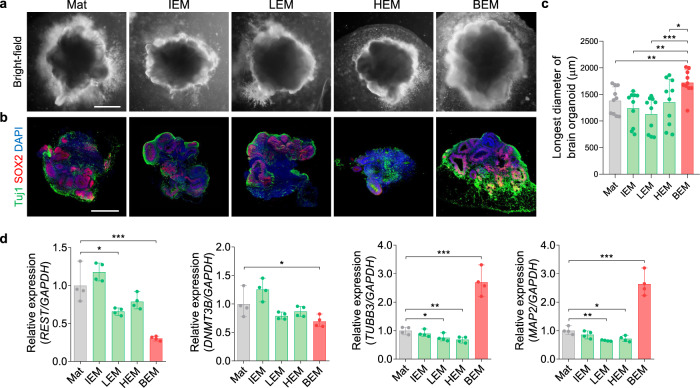
The tissue-specific effects of 3D ECM hydrogels on brain organoid development. **a** Bright-field images of brain organoids encapsulated in ECM hydrogels derived from different decellularized organs [brain (BEM), intestine (IEM), liver (LEM), and heart (HEM)] at day 30 (scale bars = 500 μm). **b** Immunostaining of brain organoids on day 30 for Tuj1 and SOX2 (scale bars = 500 μm). **c** Image-based quantification of the longest diameter of brain organoid at day 30 (*n* = 10 per group, BEM versus Mat *p* = 0.0069, BEM versus IEM *p* = 0.0013, BEM versus LEM *p* = 0.0002, BEM versus HEM *p* = 0.0301). **d** The gene expression analysis of brain organoids at day 30 by qPCR for transcriptional factors (*REST*, *DNMT3B*) and neuronal markers (*TUBB3*, *MAP2*) (*n* = 4 per group, Mat versus LEM *p* = 0.025, Mat versus BEM *p* = 0.0009 for *REST*; Mat versus BEM *p* = 0.0498 for *DNMT3B*; Mat versus LEM *p* = 0.031, Mat versus HEM *p* = 0.0085, Mat versus BEM *p* = 0.0004 for *TUBB3*; Mat versus LEM *p* = 0.0017, Mat versus HEM *p* = 0.0128, Mat versus BEM *p* = 0.0003 for *MAP2*). Mat and BEM organoids were cultured in a dish on an orbital shaker. All quantitative data are expressed as mean ± SD. Statistical differences between the groups were determined by unpaired two-tailed *t*-test (*p < 0.05, **p < 0.01, ***p < 0.001). Independent replicates for all data in (**a**–**d**) = 4. Source data are provided as a Source Data file.

BEM can provide complex networks of brain-specific ECM optimal for brain organoid development. The effects of ECM on lineage specification and organoid development from stem cells could be tissue-specific. Our previous studies have reported that the tissue-specific microenvironments provided by a decellularized tissue matrix improved the survival, differentiation, and function of various types of stem cells, reprogrammed cells, and primary cells^52–56^. The biochemical compositions of ECM hydrogel vary depending on the tissue source from which the ECM is isolated. Therefore, the tissue-specific effects of BEM for supporting the development of brain organoids were investigated by comparing the brain organoid supporting the ability of BEM-supplemented hydrogel with that of Matrigel incorporated with ECM derived from other types of tissues, including intestine (IEM), liver (LEM), and heart (HEM) (Fig. 4). At day 30, the size of organoids generated in Mat and other tissue-derived ECM hydrogels was similar (Mat; 1385 ± 255.7 μm, IEM; 1241 ± 319.3 μm, LEM; 1130 ± 327.4 μm, HEM; 1355 ± 431.7 μm), whereas the brain organoids cultured in the BEM hydrogel were larger in diameter (BEM; 1723 ± 239.4 μm) than the control groups (Fig. 4a, c). In addition, BEM facilitated the formation of larger neuroepithelial structures and increased Tuj1^+^ neuronal populations (Fig. 4a, b). Next, the gene expression profiles of the brain organoids cultured in various matrices at day 30 were compared (Fig. 4d). The expression of repressor element 1 silencing transcription factor (*REST*) and *DNMT3B*—which are known to suppress neuronal differentiation—was the lowest in BEM organoids among the tested groups. When compared with the organoids in Mat and other ECM control groups, the expression of neuronal markers *TUBB3* and *MAP2* was significantly upregulated in the BEM group, demonstrating the neuronal differentiation accelerated by the BEM signals. Overall, these results prove the critical role and necessity for tissue-specific ECM in organoid development.

### Microfluidic BEM conditions improve the survival of brain organoids

**Fig. 5:**
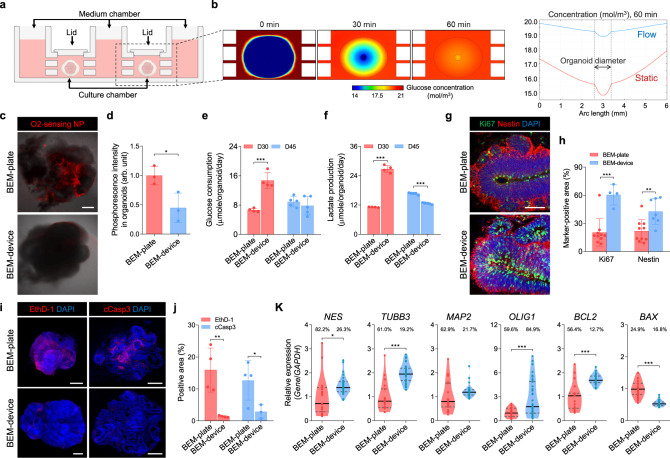
Microfluidic dynamic culture supports the proliferation and prevents the apoptosis of brain organoids grown in the 3D BEM. **a** Schematic diagram of the microfluidic device for the brain organoid culture. **b** Computational simulation of glucose concentration within the brain organoid and its surroundings in a culture chamber under the fluid flow conditions (left). Comparison of glucose concentration within the brain organoid in the presence and absence of fluid flow (right). **c** Representative merged images showing organoids (gray) and phosphorescence of oxygen-sensing (PtTFPP-PUAN) nanoparticles at 754 nm (red) for BEM-plate (top) and BEM-device (bottom) organoids on day 30 (scale bars = 200 μm, independent replicates = 4). **d** The normalized mean phosphorescence intensity in BEM-plate and BEM-device organoids (*n* = 3 per group, BEM-plate versus BEM-device *p* = 0.032, independent replicates = 4). Measurement of (**e**) glucose level in organoids and (**f**) lactate level in the medium at days 30 and 45 (*n* = 4 for D30 and *n* = 5 for D45, BEM-plate versus BEM-device *p* < 0.0001 at D30 and *p* < 0.0001 at D45, independent replicates = 2 for day 30 and 1 for day 45). **g** Immunostaining for the proliferation marker Ki67 and the progenitor marker Nestin in the BEM-plate and BEM-device organoids (scale bars = 50 μm, independent replicates = 3). **h** The quantification analyses of Ki67^+^ and Nestin^+^ cells in the BEM-plate and BEM-device organoids (*n* = 10 for BEM-plate group, and *n* = 4 for Ki67^+^ and *n* = 7 for Nestin^+^ in BEM-device group, BEM-plate versus BEM-device *p* = 0.0004 for Ki67 and *p* = 0.005 for Nestin, independent replicates = 3). **i** Brain organoids stained with ethidium homodimer-1 (EthD-1) to label dead cells at day 30 and cleaved caspase-3 (cCasp3) at day 45 (scale bars = 500 μm). **j** Quantification of EthD-1^+^ and cCasp3^+^ area per organoid (*n* = 4 for BEM-plate group, *n* = 4 for EthD-1^+^ and *n* = 3 for cCasp3^+^ in BEM-device group, BEM-plate versus BEM-device *p* = 0.0056 for EthD-1 and *p* = 0.049 for cCasp3, independent replicates = 3). **k** Differential gene expression analyses by qPCR with BEM-plate and BEM-device organoids. One sample was prepared from a single organoid. The coefficient of variations is dictated above the bars for each group. Data are expressed as violin plots. Dark gray dashed lines and black lines indicate 25–75% quartiles and median, respectively (*n* = 19 for BEM-plate group and *n* = 30 for BEM-device group in all markers except for *OLIG1*, *n* = 29 for BEM-plate group and *n* = 35 for BEM-device group in *OLIG1*, BEM-plate versus BEM-device *p* = 0.0102 for *NES, p* < 0.0001 for *TUBB3*, *p* = 0.0001 for *OLIG1*, *p* < 0.0001 for *BCL2*, and *p* < 0.0001 for *BAX*, independent replicates = 4). All analyses were performed over 30 days in the culture. All data are expressed as mean ± SD, otherwise stated separately. Statistical differences between the groups were determined by unpaired two-tailed *t*-test (*p < 0.05, **p < 0.01, ***p < 0.001 versus BEM-plate grou*p*). Source data are provided as a Source Data file.

A low level of fluid flow existing in the cerebrospinal and interstitial spaces of the brain has been found to play an essential role in supporting the development of ventricle-like structures and the formation of the neuronal layers in the cerebral cortex^57^. Cerebrospinal fluid circulation induces pulsatile and bi-directional flow exchange at the blood barrier and the borders between cerebrospinal fluid and interstitial fluid spaces^58,59^. Therefore, we hypothesized that the presence of fluid flow that mimics the bi-directional cerebrospinal fluid would be able to amplify the effects of BEM for brain organoid development by facilitating molecular diffusion in and out of the organoids, leading to further improvement of neuronal differentiation and ventricle-like structure formation in developing organoids. Based on these considerations, a multi-layered chamber microfluidic device was developed that can generate precisely controlled fluid flow (Fig. 5a and Supplementary Fig. 2a, b). The device consists of three layers with five chambers that are fluidically connected by microchannels, enabling continuous fluid perfusion between the chambers without the need for external tubing and pumping. Two chambers are designed for brain organoid cultures and three chambers for medium reservoirs. Fluid flow through the microchannels connecting the chambers could be generated by hydrostatic pressure created by different medium levels in the chambers (Supplementary Fig. 2c), which was simply achieved by placing the device on a bi-directional laboratory rocker. Simulations preceding the design of the device (Supplementary Table 5) confirmed that our device could mediate the effective transfer of nutrients, such as glucose, into large organoids embedded in hydrogels (Supplementary Fig. 10). Four different designs of the device were simulated; (1) *z*1-channel model, (2) *z*3-channel model, (3) *y*5-channel model, and (4) *y*5-*z*3-channel model comprising a combination of *z*3- and *y*5-channel designs. Based on the simulation results of glucose transfer under gravity-driven medium flow, the *y*5-*z*3-channel model was adopted as a final device design for brain organoid culture, which shows more uniform glucose gradient profiles in the vertical axis (Supplementary Fig. 10a) and greater glucose transfer to the organoids (Supplementary Fig. 10b). As our approach does not require complicated settings with pumps and tubing, a large number of devices could be operated in parallel using a single rocker system. Thus, large-scale organoid cultures and high-throughput assays and screening are possible with our device under microfluidic conditions.

To check whether our device platform can improve molecular diffusion into organoids, after stabilization of brain organoids in BEM hydrogel for four days, the organoids embedded in BEM hydrogel were transferred into a 24-well plate (BEM-plate) or microfluidic device (BEM-device) on the laboratory rocker (Fig. 1a). The computational simulation of glucose diffusion through the brain organoids in the device predicted that at a given fluid flow, the glucose present in the medium is actively transferred to the brain organoids, which equilibrated with the glucose concentration in the medium within 60 min of dynamic culture (Fig. 5b). However, a low level of glucose was diffused into the organoids in the well plate under static conditions without medium flow (Supplementary Fig. 11a). Next, intra-organoid oxygen levels were visualized on day 30 by incorporating oxygen-sensing phosphor nanoparticles composed of Pt(II) meso-tetra(pentafluorophenyl)porphine (PtTFPP)-poly(urethane acrylate nonionomer) (PUAN)^60^. Higher oxygen levels lead to lower phosphorescence of the nanoparticles, which is attributed to collisional quenching between oxygen molecules and PtTFPP (Supplementary Fig. 11b). When the oxygen-sensing nanoparticles were incorporated in the BEM-plate and BEM-device organoids on day 30, the normalized mean phosphorescence intensity of the BEM-plate organoids was more than two-fold higher than that of the BEM-device organoids (Fig. 5c, d). This significant difference was more pronounced in the core region of the organoids. Therefore, our results indicate that the BEM-device conditions allowed for significantly higher and more uniform intra-organoid oxygen levels. This observation together with glucose diffusion simulation demonstrates that the precise control of the medium flow at a microscale level may be more effective for oxygen/nutrient supply and molecular exchange in organoids than irregular dynamic flow under bulk scale conditions.

Consequently, BEM-device organoids exhibited higher cell proliferation and less cell death, and a notably larger size than BEM-plate organoids (Fig. 5e–j, and Supplementary Fig. 12a–c). Proliferative cells mainly undergo anaerobic glycolysis converting glucose into lactate, whereas postmitotic neurons undergo a switch towards mitochondrial metabolism^61^. Quantification of metabolite glucose and lactate at day 30 revealed that BEM organoids cultured in the device consumed more glucose and secreted more lactate, compared to those ones cultured in the plates, suggesting that BEM-device organoids display the metabolic profile of higher proliferative cells (Fig. 5e, f). By day 45, BEM-plate organoids maintained the levels of glucose consumption and lactate production, whereas the levels decreased in BEM-device organoids, which may indicate that cells in BEM-device organoids switched to differentiation metabolic state. In the BEM organoids cultured in the device, the neuroepithelium was organized in a manner reminiscent of the early developing cortex, where cells positive for the proliferation (Ki67) and NP markers (Nestin) were abundant at the apical surface of the VZ (Fig. 5g). Compared to the BEM-plate organoids, the BEM-device organoids contained a larger number of Ki67^+^ and Nestin^+^ cells (Fig. 5h). Significantly less necrotic areas were found in BEM-device organoids than in BEM-plate organoids (Fig. 5i, j). When the apoptotic core regions were analyzed by quantifying the area stained with an active form of Caspase 3 at day 45 (Fig. 5i), cleaved Caspase 3^+^ apoptotic cells were observed in the BEM-plate and BEM-device organoids with the average area of 12.7 ± 6.2% and 2.9 ± 2.2%, respectively (Fig. 5j). Only very few numbers of Caspase 3^+^ cells were found in the core region of BEM-device organoids. An increase in the proliferative cell population along with reduced apoptosis in the BEM-device organoids resulted in noticeably larger organoids than that in the BEM-plate organoids (Supplementary Fig. 12a, b). Some BEM-device organoids grew up to 8 mm in diameter by day 60 (Supplementary Fig. 12c). Interestingly, the presence of BEM is likely to prevent the formation of necrotic clusters that were seen in Mat organoids (Supplementary Fig. 12d, e). To confirm that the microfluidic device allows better oxygen supply to the core region of the organoids, immunohistochemical staining for a hypoxia marker hypoxia-inducible factor-1α (HIF-1α) was performed at day 120 (Supplementary Fig. 12f, g). HIF-1α-positive area in the BEM-device organoids was significantly smaller than that of Mat and BEM organoids cultured in the plates, indicating that hypoxia in the organoid core was alleviated by microfluidic culture. These results support again that the microfluidic device facilitates oxygen transfer to the core of large organoids, resulting in the prevention of necrotic core formation via improved cell survival and decreased apoptosis. BEM-device organoids contained a larger number of FOXG1^+^ lobes than BEM-plate organoids (Supplementary Fig. 12h). The periodic application of bi-directional medium flow in the microfluidic device contributed to improving the overall quality of the brain organoids. When we examined the expression of a choroid plexus (CP) epithelial cell marker transthyretin (TTR) in Mat, BEM, and BEM-device organoids, all groups were found to contain similar TTR^+^ area (Supplementary Fig. 12i, j), suggesting that BEM did not affect CP development.

Importantly, the use of a microfluidic device reduced the variability in the brain organoids grown in BEM hydrogel. The gene expression profiles of organoids in the BEM-plate and BEM-device conditions were analyzed using a single organoid for each group (Fig. 5k). The relative expression of the neuronal markers, *Nestin*, *TUBB3, MAP2*, and oligodendrocyte marker oligodendrocyte transcription factor 1 (*OLIG1*) was higher in the BEM-device organoid than that in the BEM-plate organoid. Relative to the BEM-plate organoid, the expression of the anti-apoptotic marker B-cell lymphoma 2 (*BCL2*) was upregulated, while that of the apoptotic marker BCL2-associated X (*BAX*) was downregulated in the BEM-device organoid. Additionally, the variation in gene expression in the BEM-device organoid was much less than that in the BEM-plate organoid—as indicated by the lower variation coefficient value—with the exception of *OLIG1* (Fig. 5k). The BEM organoids cultured in multi-well microfluidic device designed to have 24 chambers for scalable organoid culture (Supplementary Fig. 13a, b and Supplementary Video 3) not only displayed relatively higher expression of *PAX6* and *TUBB3*, but also showed significantly less variation in the expression levels of both genes, compared to the BEM organoids cultured using conventional plates (Supplementary Fig. 13c–f). Taken together, these results demonstrate that the microfluidic organoid culture platform increased neuroepithelial outgrowth and overall organoid size by promoting cell proliferation and reducing apoptosis and necrotic cluster. In addition, the microfluidic BEM condition might reduce the variation between organoid samples in terms of gene expression profiles, thereby improving the homogeneity of brain organoids that is critically important for precise evaluation and data interpretation during drug testing and disease modeling.

### The microfluidic BEM platform enhances corticogenesis and radial glial cell generation in brain organoids

**Fig. 6:**
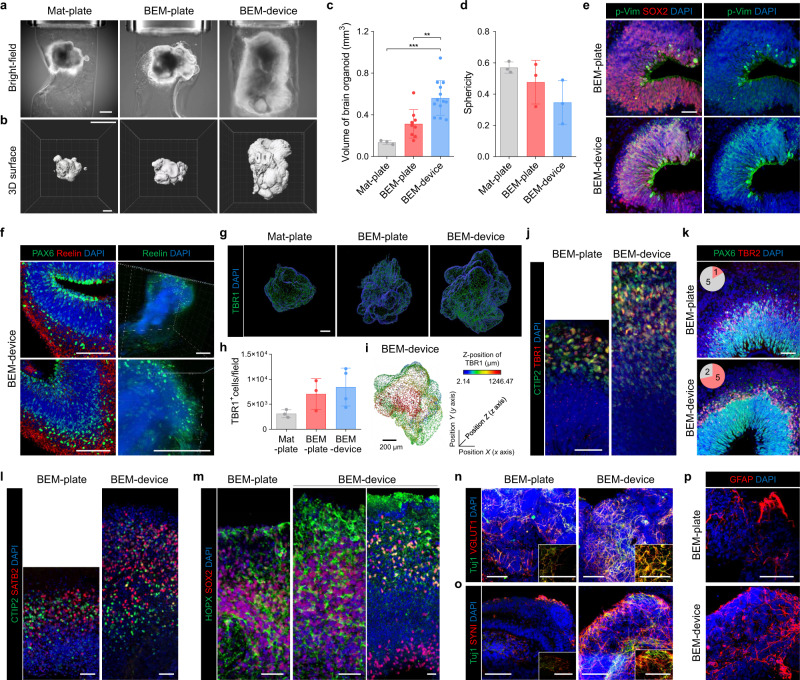
Bioengineering of the brain organoids by the microfluidic BEM system improves radial glial generation and cortical organization. **a** Light-sheet microscopic bright-field images of 3D brain organoids encapsulated in Mat and BEM cultured in a plate or microfluidic device at 60 days of culture (scale bar = 500 μm, independent replicates = 3). **b** Reconstructed light-sheet microscopic images of 60-day Mat-plate, BEM-plate, and BEM-device organoids (scale bar = 500 μm, independent replicates = 3). **c** Quantification analyses for the 3D organoid volume (*n* = 3, 9, and 13 for Mat-plate, BEM-plate, and BEM-device groups, respectively, Mat-plate versus BEM-device *p* = 0.0007, BEM-plate versus BEM-device *p* = 0.0014, independent replicates = 3), and (**d**) sphericity (*n* = 3 per group, independent replicates = 3) of brain organoids using IMARIS software. **e** Representative immunostaining images for mitotic radial glia marker phosphorylated vimentin (p-Vim) and SOX2 in the BEM-plate and BEM-device organoids at day 30 (scale bar = 50 μm, independent replicates = 5). **f** Expression of the radial glial marker PAX6 and extracellular glycoprotein marker Reelin in BEM-device organoids (left panel, scale bars = 200 μm, independent replicates = 2) and 3D imaging of Reelin expression in BEM-device organoids at day 30 (right panel, scale bars = 200 μm, technical replicates = 5). **g** 3D plotting and (**h**) quantification of deep-layer marker TBR1-expressing cells in Mat-plate, BEM-plate, and BEM-device organoids at day 30 using IMARIS software (*n* = 3 for Mat-plate and BEM-plate groups, and *n* = 4 for BEM-device group, independent replicates = 3). **i** 3D plotting analysis of TBR1^+^ cells in BEM-device organoids on different z-positions in the radiometric color spectrum (scale bar = 200 μm, biological replicates = 3). **j** Immunohistochemically stained sections for TBR1 and CTIP2 at day 45 (scale bar = 50 μm, independent replicate = 1). **k** Immunohistochemical staining images for subventricular marker TBR2 and PAX6 at day 45 (scale bar = 50 μm, independent replicates = 2). Pink color in the pie-charts indicates the number of cortical structures with separated layers of TBR2^+^ and PAX6^+^ cells, and grey color indicates no layer preferences. **l** Immunostaining images showing CTIP2 and superficial-layer neuron marker SATB2 (scale bars = 50 μm, independent replicate = 1), and (**m**) basal radial glia marker SOX2 and HOPX at day 75 (scale bars = 50 μm, independent replicate = 1). **n** Immunostaining of Tuj1 and glutamatergic neuron marker VGLUT1 (independent replicates = 2), (**o**) presynaptic marker SYNI (independent replicates = 2), and (**p**) astrocyte marker GFAP in the BEM-plate and BEM-device organoids at day 60 (scale bars = 100 μm, independent replicates = 3). All data are presented as mean ± SD. Statistical differences between the groups were determined with unpaired two-tailed *t*-test (**p < 0.01, ***p < 0.001). Source data are provided as a Source Data file.

Enhancement of organoid growth and cortical structure formation by microfluidic BEM culture was assessed by 3D light-sheet microscopy and subsequent morphological analysis (Fig. 6a–c). Microfluidic culture improved oxygen supply to the interior of the organoids that leads to increased proliferation, expanded NP population, and thicker neuroepithelium. Accordingly, the microfluidic BEM device induced significantly faster growth and development of more complex surface structure in brain organoids than the control culture conditions (Mat-plate, BEM-plate) (Fig. 6a, b). The overall volume of organoids in Mat-plate, BEM-plate, and BEM-device groups was 0.14 ± 0.02 mm^3^, 0.31 ± 0.14 mm^3^, and 0.56 ± 0.17 mm^3^, respectively, indicating a marked difference between the groups (Fig. 6c). The degree of sphericity was 0.57 ± 0.03, 0.47 ± 0.14, and 0.35 ± 0.11 in Mat-plate, BEM-plate, and BEM-device organoids, respectively (Fig. 6d). Mat organoids were found to be morphologically smoother and smaller relative to the BEM organoids (Fig. 6a–d). The sectioned, nuclei-stained images obtained by confocal microscopy also showed larger and more elongated lobes in the BEM organoids compared to that in the Mat organoids (Supplementary Fig. 14a). These results suggest that BEM promoted volume expansion, structural maturation, and the formation of the elongated epithelium in organoids, and microfluidic devices magnified these effects of BEM by providing favorable dynamic microenvironments for the brain organoid cultures. Overall, BEM combined with a microfluidic system facilitated organoid growth and induced a high level of cellular accumulation, leading to the formation of highly complex and more advanced structural developments^62^.

The efficient supply and exchange of nutrients/oxygen by well-controlled bi-directional fluid flow promoted NP proliferation with the expansion of radial glia during the early stage of brain organogenesis. In BEM-device organoids, the increase in proliferation coincided with an expansion of the NP pool, as shown by immunostaining for radial glia markers p-Vim, SOX2, and PAX6 (Fig. 6e and Supplementary Fig. 14b, c). The larger number of p-Vim^+^ mitotic RGCs was detected in the BEM-device organoids than that in BEM-plate organoids (Fig. 6e and Supplementary Fig. 14d). At 30 days, some of the p-Vim^+^ and PAX6^+^ NPs showing a typical radial glial morphology with processes contacting both the apical and basal surfaces of the neuroepithelium were observed in the BEM-device organoids (Supplementary Fig. 14c). Cortical layer formation in vivo depends upon the proper migration of postmitotic neurons from their sites of origin to their final destinations, and neuronal migration is preciously orchestrated by various intrinsic and extrinsic factors^63,64^. The presence of Cajal-Retzius cells, which are involved in neuronal migration and cortical lamination^64,65^, was examined by immunostaining for Reelin in the BEM-device organoids at day 30 (Fig. 6f). The sectioned organoid images stained for Reelin revealed a wide distribution of Reelin^+^ signals in the most superficial regions (Fig. 6f, left panel). The 3D imaging analysis indicated more dispersed signals of Reelin as a secreted factor (Fig. 6f, right panel). Therefore, the enrichment of the cell types and their roles supporting cortical layer formation in BEM-device organoids was speculated.

As a result, the microfluidic BEM system significantly promoted corticogenesis in developing brain organoids. The expression of a deep-layer marker TBR1 in organoids at day 30 was examined by confocal microscopy (Fig. 6g–i). Compared to Mat organoids, the BEM organoids contained an increased TBR1^+^ cell population (Fig. 6g, h). TBR1 expression with a broader spectrum of the z-axis along the cortical layer was detected in the BEM-device organoids (Fig. 6i and Supplementary Fig. 15a, b). BEM-device organoids formed a thicker deep layer containing CTIP2^+^ and TBR1^+^ neurons at day 45 (Fig. 6j). In addition, a thicker layer of TBR2^+^ cells with a highly packed band of cells in the cortical region of the BEM-device organoids was also observed at day 45 (Fig. 6k), indicating that the formation of the SVZ layer with a high density of cells was promoted in brain organoids cultured under BEM-device conditions. Chondroitin sulfate proteoglycan (CSPG)^+^ layer indicating preplate splitting was observed in BEM-device organoids (Supplementary Fig. 15c). BEM and BEM-device organoids displayed a mixed population of deep-layer marker CTIP2^+^ neurons and upper-layer marker SATB2^+^ neurons 75 days of culture (Fig. 6l). Compared to BEM organoids, a substantially thicker layer of SATB2^+^ neurons was formed in the BEM-device organoids (Fig. 6l). Both BEM and BEM-device organoids contained SOX2^+^ and HOPX^+^ basal radial glia-like populations at day 75. A SOX2^+^/HOPX^+^ outer SVZ structure separated from SOX2^+^/HOPX^−^ VZ was more frequently observed in the BEM-device organoids (Fig. 6m).

BEM-device organoids innately developed a higher population of ionized calcium-binding adaptor molecule 1 (IBA1)^+^ and CD68^+^ microglia (Supplementary Fig. 16a, b). IBA1^+^ microglia were initially found sparse in small clusters at day 30, but IBA1^+^ and CD68^+^ cells were present throughout the BEM-device organoids at day 74. Quantification of microglial population in Mat, BEM, and BEM-device organoids at day 74 indicated the highest numbers of IBA1^+^ and CD68^+^ microglia in the BEM-device organoids (Supplementary Fig. 16c, d). By day 60, the BEM-device organoids showed a higher density of Tuj1 and mature neuronal marker VGLUT1 or presynaptic marker Synapsin I (SYNI) co-positive cell populations than BEM-plate organoids (Fig. 6n, o). At this time point, only BEM-device organoids expressed a mature synaptic marker, postsynaptic density protein 95 (PSD95) (Supplementary Fig. 16e). In addition, glial fibrillary acidic protein (GFAP)^+^ astrocytes, which are detected in the late stages of brain organogenesis, were more abundantly present in BEM-device organoids (Fig. 6p). The combination of BEM with a microfluidic system facilitated the maturation of neural populations in brain organoids even without exogenous neurotrophic factors in a relatively short period. Overall, bioengineering organoid culture with brain ECM- and cerebrospinal fluid-mimetic cues increased populations of proliferative progenitors and Reelin-secreting Cajal-Retzius cells, resulting in a robust organoid growth and accelerated cortical layer organization along the radial axis that contains mature neurons and glial cells. This contributes to the formation of elongated and continuous cortical layers and increases the complexity of the structures.

### The further maturation of brain organoids at the molecular and functional levels by micro-controlled fluid flow

**Fig. 7:**
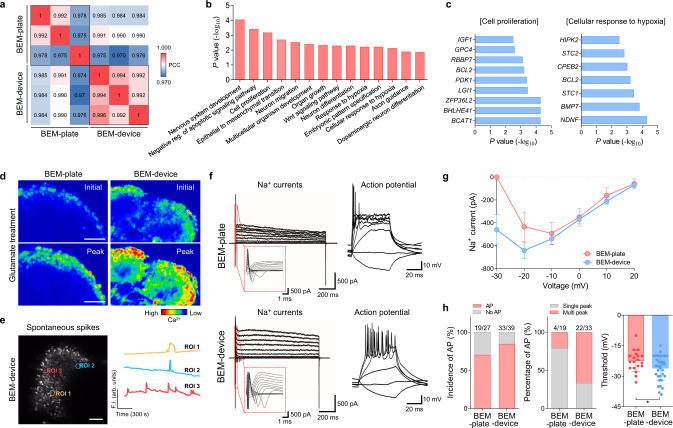
The transcriptome profile analyses and electrophysiological function characterization of brain organoids cultured in the microfluidic BEM system. **a** Pearson’s correlation matrix for whole-genome profiles in BEM-plate and BEM-device organoids (*n* = 3, independent replicate = 1). Pearson’s correlation coefficient (PCC) values are indicated in each box. **b** The top 14 enriched gene ontology (GO) terms of upregulated genes in the BEM-device group versus the BEM-plate group (shown in terms of *p* values). **c** Lists of differentially upregulated genes classified in two GO functional categories; ‘Cellular response to hypoxia’ and ‘Cell proliferation’. **d** Ratiometric images of calcium imaging before and after 100 μM glutamate treatment in Fluo-4 AM-loaded BEM-plate and BEM-device organoids (scale bars = 50 μm, independent replicates = 4). The color scale indicates fluorescence intensity of Fluo-4 AM. **e** A fluorescence image showing spontaneous calcium (Ca^2+^) transient of cells in BEM-device organoids (left) (scale bar = 100 μm, independent replicates = 3). Representative time-course peaks showing spontaneous changes in Fluo-4 AM fluorescence intensity measured in cells in the BEM-device organoid (right). Traces were obtained from the region of intensity (ROI) marked on the fluorescence image. **f** Representative current traces recorded in a neuron with a voltage-clamp mode (left two panels) and representative traces of evoked action potentials (APs) recorded in a neuron with a current-clamp mode (right two panels) in brain organoids grown in either a BEM-plate (top panels) or a BEM-device (bottom panels). **g** Quantification of sodium (Na^+^) currents in response to increased voltage steps starting from −30 mV to +20 mV (10 mV step size) in neurons within brain organoids grown in either a BEM-plate or a BEM-device (*n* = 5). **h** Quantification of AP incidence (BEM-plate: *n* = 27; BEM-device: *n* = 39, independent replicates = 7), spike numbers (single versus multiple) in each condition, and threshold potentials to evoke AP (BEM-plate: *n* = 19; BEM-device: *n* = 33, unpaired two-tailed *t*-test (*p < 0.05), BEM-plate versus BEM-device *p* = 0.0380). Brain organoids cultured for (**a**–**e**) 75 days and (**f**–**h**) 60 days were analyzed. All data are expressed as mean ± SD. Source data are provided as a Source Data file.

The microfluidic BEM system facilitated the further maturation of brain organoids in terms of transcriptome profiles and electrophysiological properties. To investigate the effects of microscale dynamic flow on the transcriptome profiles in brain organoids, RNA-sequencing was performed with three batches of BEM-plate and BEM-device organoid samples at day 75 (Fig. 7a–c and Supplementary Fig. 17). A heatmap of the differential expression analysis showed that 214 genes were upregulated and 802 genes were downregulated in BEM-device organoids compared with that in the BEM-plate organoids (Supplementary Fig. 17a). Pearson’s correlation coefficient values between samples in the same group and principal component analysis (PCA) demonstrated that there was less variability between the samples in the BEM-device group than in those in the BEM-plate group (Fig. 7a). GO analysis identified the enrichment of neuronal differentiation, axonal guidance, and neuronal migration among upregulated genes in the BEM-device group compared to the BEM-plate group (Fig. 7b). Especially, the expression of genes involved in mediating the cellular response to hypoxia and cell proliferation increased (Fig. 7c). GSEA analysis showed that genes involved in apoptosis were enriched in the BEM-plate group (Supplementary Fig. 17b). These results coincide with the earlier observation that bi-directional fluid flow generated by our microfluidic platform could provide higher oxygen content for the organoids, leading to increased cell expansion and decreased apoptosis. BEM-device organoids also showed upregulated expression of risk genes related to schizophrenia, autism, and Parkinson’s disease, compared with BEM-plate organoids (Supplementary Fig. 17c).

Improvement of the electrophysiological functional properties of cells in brain organoids by microfluidic 3D BEM culture with the dynamic flow was assessed by calcium influx imaging and a whole-cell patch-clamp recording at 75 and 60 days of culture, respectively. Cells that responded to glutamate in BEM-device organoids showed a more significant increase in intracellular Ca^2+^ levels, compared with those in the BEM-plate organoids (Fig. 7d). A few cells in the BEM-device organoids exhibited spontaneous spikes of Ca^2+^ influx, even in the absence of any neurotransmitters (Fig. 7e and Supplementary Video 4). An electrophysiological study using whole-cell patch clamping demonstrated that recorded neurons in both organoid groups showed Na^+^ currents in response to voltage steps (Fig. 7f). The average amplitudes of Na^+^ currents in response to voltage ramps between −30 mV and 20 mV were much higher for neurons in BEM-device organoids than those in BEM-plate organoids (Fig. 7g). Neurons in both organoid groups were capable of eliciting APs in response to depolarizing current injection, which are similar to those of mature functional neurons (Fig. 7f). Neurons in BEM-device organoids were able to produce APs at a higher rate, and higher percentages of these cells fired multi-spikes of APs compared to those in BEM-plate organoids (Fig. 7h). The threshold levels of APs were also increased in BEM-device organoids (Fig. 7h). Importantly, a postsynaptic current (PSC) was detected only in the BEM-device organoids, even though this was rare, indicating the formation of a postsynaptic compartment and the possible establishment of synaptic networks (Supplementary Fig. 18). Our results support that the organoid culture in microfluidic BEM condition could improve the electrophysiological functional properties of brain organoids. Overall, a precise perfusion culture of human cerebral organoids in our customized bioreactor based on a microfluidic device platform facilitated the efficient exchange of nutrients, oxygen, and waste, leading to volumetric expansion with complex structures, and further improvement in corticogenesis and electrophysiological functionalities of individual neuronal populations.

### Reproducible improvement in brain organoid development by microfluidic BEM platforms

**Fig. 8:**
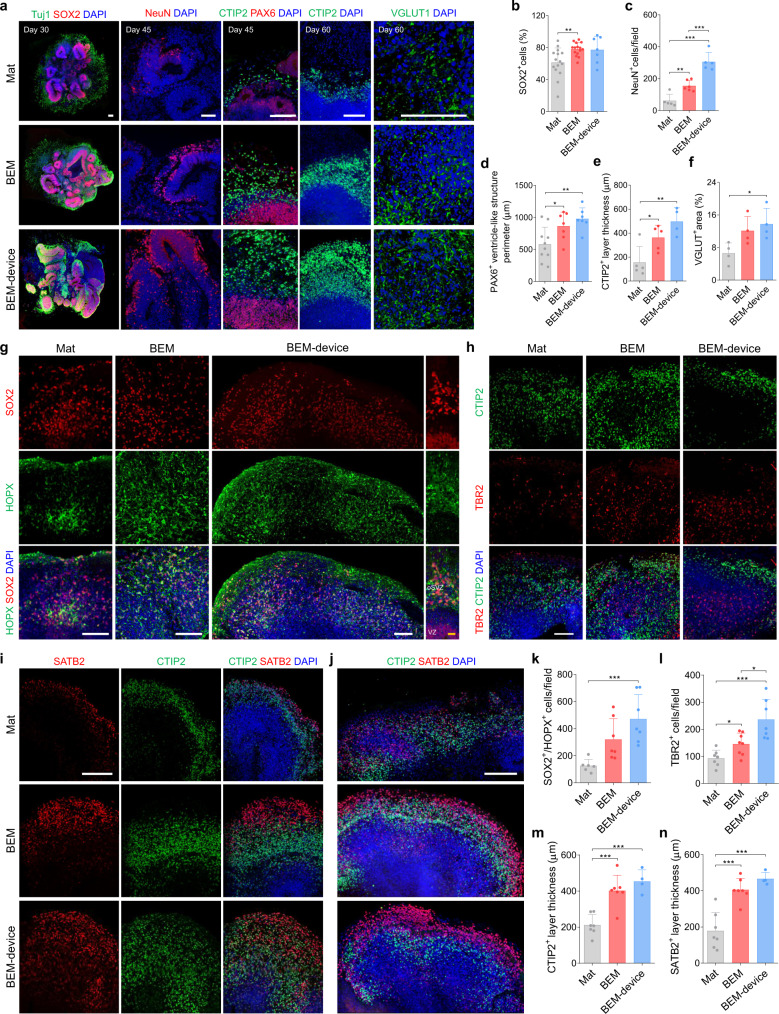
Combination of BEM and microfluidic device reproducibly improves organization of progenitor zones and cortical layers in brain organoids. **a** Immunostaining of Tuj1 and SOX2 (day 30), NeuN, PAX6, and CTIP2 (day 45), CTIP2 and VGLUT1 (day 60) in Mat, BEM, and BEM-device organoid groups (scale bars = 100 μm, independent replicates = 2). Quantification of (**b**) SOX2^+^ cells at day 30 (*n* = 15 for Mat and BEM groups and *n* = 7 for BEM-device group, Mat versus BEM *p* = 0.003, independent replicates = 3), (**c**) NeuN^+^ cells at day 45 (*n* = 5 for Mat and BEM-device groups and *n* = 6 for BEM group, Mat versus BEM *p* = 0.0022, Mat versus BEM-device *p* < 0.0001, BEM versus BEM-device *p* = 0.0004, independent replicates = 2), (**d**) PAX6^+^ ventricle^-^like structure perimeter at day 45 (*n* = 10 for Mat group, and *n* = 7 for BEM and BEM-device groups, Mat versus BEM *p* = 0.0357, Mat versus BEM-device *p* = 0.0036, independent replicates = 2), (**e**) CTIP2^+^ layer thickness at day 60 (*n* = 5 for Mat and BEM groups, and *n* = 4 for BEM-device group, Mat versus BEM *p* = 0.0243, Mat versus BEM-device *p* = 0.0047, independent replicates = 2), and (**f**) VGLUT^+^ cell area at day 60 (*n* = 4, Mat versus BEM-device *p* = 0.0258, independent replicates = 2). **g** Immunostaining of basal radial glia markers SOX2 and HOPX at day 100 (scale bars = 100 μm for white line and 20 μm for yellow line, independent replicates = 2). Note the SOX2^+^/HOPX^+^ outer subventricular (SVZ) region clearly separated from the SOX2^+^/HOPX^-^ ventricular zone (VZ)-like region in BEM-device organoid. Immunostaining of (**h**) SVZ marker TBR2 and deep-layer marker CTIP2 at day 100 (scale bar = 100 μm, independent replicates = 2), and (**i**, **j**) upper-layer marker SATB2 and CTIP2 at (**i**) day 100 (scale bar = 200 μm, independent replicates = 2) and (**j**) day 120 (scale bar = 200 μm, independent replicates = 2) in Mat and BEM organoids cultured in the petri dish on the orbital shaker, and BEM-device organoids cultured in the microfluidic device on the bi-directional rocker. Quantification of (**k**) SOX2^+^/HOPX^+^ cells (*n* = 6 for Mat group, and *n* = 7 for BEM and BEM-device groups, Mat versus BEM-device *p* = 0.0008, independent replicates = 2), **l** TBR2^+^ intermediate progenitor cells (*n* = 7 for Mat and BEM-device groups, and *n* = 8 for BEM group), Mat versus BEM *p* = 0.0147, Mat versus BEM-device *p* = 0.0005, BEM versus BEM-device *p* = 0.0101, independent replicates = 2), (**m**) the thickness of CTIP2^+^ deep-layer neurons at day 100 (*n* = 7 for Mat and BEM groups, and *n* = 4 for BEM-device group, Mat versus BEM *p* = 0.0004, Mat versus BEM-device *p* = 0.0001, independent replicates = 2), and (**n**) the thickness of SATB2^+^ superficial-layer neurons at day 100 (*n* = 7 for Mat and BEM groups, and *n* = 4 for BEM-device group, Mat versus BEM *p* = 0.0003, Mat versus BEM-device *p* = 0.0004, independent replicates = 2). All data are presented as mean ± SD. Statistical differences between the groups were determined with unpaired two-tailed *t*-test (*p < 0.05, **p < 0.01, and ***p < 0.001). Source data are provided as a Source Data file.

We examined whether microfluidic BEM culture supports reproducible generation of brain organoids exhibiting improved neurogenesis and structural maturation. To this end, neurogenesis of BEM-device organoids was directly compared with that of BEM organoids (in petri dish) cultured using the standard conventional orbital shaker method. At day 30, BEM organoids cultured in dish on the orbital shaker and BEM-device organoids had approximately 1.2 times higher number of SOX2^+^ NPs than Mat organoids in dish on the orbital shaker (Fig. 8a, b). At day 45, BEM-device organoids contained substantially higher number of NeuN^+^ neurons compared to organoids in other two groups cultured with an orbital shaker (Fig. 8a, c). BEM-device organoids also displayed significantly larger PAX6^+^ ventricle-like structures than orbital shaker-cultured organoids (Fig. 8a, d). At day 60, the presence of the thickest layer of CTIP2^+^ deep-layer neurons and the largest area of VGLUT1^+^ excitatory neurons were observed in the BEM-device organoids (Fig. 8a, e, f). We reproducibly observed that the use of microfluidic device reduces the variation in the size of brain organoids derived from iPSCs with different passage numbers as indicated by the lowest coefficient of variation in the BEM-device organoids (Supplementary Fig. 19).

In addition, the effects of BEM and microfluidic device were further confirmed by generating organoids using another iPSC line (KYOU-DXR0109B, ATCC) (Supplementary Fig. 20). When BEM-device organoids were directly compared with Mat and BEM organoids cultured on an orbital shaker, similar features of BEM-device organoids, including faster growth, substantially longer diameter, and increased neuronal marker expression, were reproducibly observed with the new iPSC line. Combination of BEM and microfluidics enhanced neurogenesis during the development of brain organoids derived from new cell line, as indicated by more densely packed SOX2^+^ NPs at the apical side at day 30 (Supplementary Fig. 20a), and thicker CTIP2^+^ deep layer and SATB2^+^ superficial-layer neurons observed at day 60 (Supplementary Fig. 20b). SATB2^+^ neurons were barely observed in Mat and BEM organoids at this time point (Supplementary Fig. 20b). BEM-device organoids from the new iPSC line at day 60 were also notably larger than Mat and BEM organoids without microfluidic culture, while showing the reduced variation in the organoid size (Supplementary Fig. 20c). The expression of several neuronal markers was significantly elevated in the BEM-device organoids compared to Mat and BEM organoids at day 60 (Supplementary Fig. 20d). These data validate the reproducibility of our culture system based on BEM and microfluidics for reliable production of human brain organoids, irrespective of iPSC lines.

Specialized basal radial glia (bRG) in the SVZ is an important hallmark of human cortical development. To analyze the distribution of cortical multi-layer neuronal subtypes in SVZ layer development, we conducted the immunostaining for several markers (TBR2, SOX2, HOPX) in the Mat, BEM, and BEM-device organoids 100 days of the culture (Fig. 8g, h). We observed outer SVZ-like structures containing HOPX^+^ bRG-like cells (Fig. 8g). The number of SOX2^+^ HOPX^+^ bRG-like cells substantially increased in the BEM and BEM-device organoids in comparison to Mat organoids (Fig. 8g, k). The highest populations of SOX2^+^ HOPX^+^ bRG-like cells were found in the BEM-device organoids. In the BEM-device organoids, we also observed SOX2^+^ HOPX^+^ region clearly separated from the SOX2^+^ HOPX^-^ VZ region (Fig. 8g). A thin SOX2^-^ HOPX^-^ layer appeared between these two regions, indicating an inner SVZ-like region (Fig. 8g). At day 100, Mat organoids contained a layer of a mixed population of TBR2^+^ cells and CTIP2^+^ neurons showing no layer preferences (Fig. 8h). In contrast, BEM and BEM-device organoids displayed CTIP2^+^ cortical plate-like layer that was formed above TBR2^+^ SVZ-like layer (Fig. 8h). The highest number of TBR2^+^ IPCs was observed in the BEM-device organoids among all groups (Fig. 8h, l). The CTIP2^+^ layers were significantly thicker in BEM and BEM-device organoids than in Mat organoids (Fig. 8m). These results demonstrate that BEM enhances the number of bRG-like cells in the SVZ layer and the microfluidic device improves the formation of multi-layer progenitor zones.

Co-staining of the brain organoids for upper-layer marker SATB2 and deep-layer marker CTIP2 on day 100 showed that all groups contained partially separated layers of early born CTIP2^+^ neurons and late-born SATB2^+^ neurons (Fig. 8i), suggesting a layer specification of deep and upper layers. Thicker CTIP2^+^ and SATB2^+^ layers were found in the BEM and BEM-device organoids compared to Mat organoids (Fig. 8i, m, n). In the BEM-device organoids, the thickness of the superficial layer was more consistent and the distribution of SATB2^+^ neurons was localized more superficially to the CTIP2^+^ neurons by day 120 (Fig. 8j). Accordingly, this two-layer separation became more distinct in BEM-device organoid by day 120 (Fig. 8j). These data support that the culture of brain organoids using BEM and microfluidic device facilitates the development of upper-layer neurons as well as deep-layer neurons.

### The feasibility of porcine BEM for culturing human brain organoids

We explored the feasibility of porcine brain tissue-derived BEM (pBEM) for brain organoid culture. Human sources may be more desirable for human brain organoids but due to a limited availability, application of BEM derived from porcine brain tissues, which can be prepared from the same donor conditions (*e.g*. brain regions, donor ages, etc.), may be a more preferred option in terms of standardization (Supplementary Fig. 21a–h). Proteomic analysis of cortex-derived pBEM confirmed similar numbers and profiles of matrisomal contents to those of human BEM (Supplementary Table 2). pBEM was found to have higher content of glycoproteins and less proteoglycans compared to human BEM, but the overall profiles of matrisome compositions were similar (Supplementary Fig. 21c–h). pBEM contained 457 brain tissue-enriched proteins, and GOBP analysis revealed that these proteins are involved in synapse signaling, nervous system development, and neurogenesis (Supplementary Fig. 21b). Brain organoid culture using pBEM and microfluidics showed similar improvement as seen in human BEM-based culture (Supplementary Fig. 22). Combination of pBEM and microfluidics promoted organoid growth, and increased the populations of PAX6^+^ progenitors, CTIP2^+^ and NeuN^+^ neurons at 60 days of culture (Supplementary Fig. 22a–f). Gene expression analyses by qPCR indicated higher neuronal gene expression (*PAX6*, *TUBB3*, and *MAP2*) in pBEM and pBEM-device organoids compared to Mat organoids (Supplementary Fig. 22g). These results may suggest that pBEM is comparable to human BEM.

We also examined (1) batch variation (different pBEM batches derived from the same donor), (2) sample variation (pBEM samples derived from different donors), and (3) region variation (pBEM derived from different brain regions). The content and composition of matrisome proteins in pBEM derived from the cortex of the same donor were similar between different preparation batches of pBEM (porcine A cortex #1 versus porcine A cortex #2) (Supplementary Fig. 21c, d). In addition, the top 10 matrisome proteins with the highest expression completely overlapped between different batches of pBEM (red highlighted) (Supplementary Fig. 21e). The variability of pBEM derived from porcine brain cortical tissues of different donors was then investigated (porcine A cortex versus porcine B cortex). The content and composition of matrisome proteins were similar, and 9 out of the top 10 proteins with the highest expression were matched (Supplementary Fig. 21f–h). When the ECM profiles of pBEM derived from different brain regions were compared (cortex versus cerebellum), the content and composition of matrisome proteins in pBEM prepared from cerebellum were different from those in pBEM derived from cortex (Supplementary Fig. 21i, j). Cerebellum-derived pBEM contained higher content of matrisome proteins (~18%) than cortex-derived pBEM (~8%). However, both samples shared 8 out of 10 proteins in the top 10 proteins with the highest expression (Supplementary Fig. 21k). A heatmap of Spearman’s rank correlation coefficients and PCA plot of all samples confirm higher degree of similarity in the pBEM derived from the same brain regions, regardless different batches or donor samples. (Supplementary Fig. 21l, m). There was less similarity between pBEM derived from cortex and cerebellum. Volcano plots of matrisome proteins identified in cortex pBEM versus cerebellum pBEM also demonstrate the substantial numbers of differentially expressed proteins (52 out of 108 matrisome proteins differentially expressed by ≥ 2 folds with *p* < 0.05) in both types of pBEM samples (Supplementary Fig. 21n). When we compared cerebral brain organoids cultured in porcine cortex- and cerebellum-derived pBEM hydrogels, interestingly they showed differential effects on the development of FOXG1^+^ forebrain regions. Brain organoids grown in cerebellum pBEM displayed smaller FOXG1^+^ area than organoids cultured in cortex pBEM, which was comparable to Mat organoids (Supplementary Fig. 23a, b), indicating that cerebellum ECM is not effective for forebrain region development. Therefore, BEM derived from different brain regions may affect the compositions of developing brain regions in the cerebral organoids.

Overall, proteomic analysis demonstrates that the ECM profile variation arising from different brain regions exists, but the batch-to-batch variation from BEM preparation and different donor samples is relatively low in the case of BEM derived from the same brain region. Given that porcine tissues with varied ages are available and also not strictly limited to specific brain regions, pBEM derived from porcine brain tissue may be able to provide a reliable source for standardization and practical applications in brain organoid culture.

### Combination of microfluidic BEM culture platform with other advanced brain organoid protocols

Finally, we tested a combination of microfluidic BEM platform with other advanced brain organoid protocols. Previously, the original cerebral organoid protocol could be improved by using poly(lactide-co-glycolide) (PLGA) fiber scaffolds and treating with short pulses of CHIR99021^20^. Thus, we combined our platform with PLGA microfilament-engineered cerebral organoid (enCOR) method by encapsulating PLGA-incorporated EBs into BEM hydrogel and culturing the constructs in our microfluidic device (enCOR-BEM-device) (Supplementary Fig. 24). We observed higher SOX2^+^ progenitor population at day 30 and higher number of NeuN^+^ neurons at day 50 in the enCOR-BEM-device organoids than in the enCOR-Mat organoids or enCOR-BEM organoids. Although these preliminary data may indicate a potential of our microfluidic BEM system to be integrated with enCOR method, more in-depth investigation would be required to check epithelium elongation and cortical layer development. For the improvement of generating region-specific organoids, our platform may also be combined with region-selective differentiation culture methods^6,8,66,67^ by supplementing BEM in the medium instead of Mat and culturing the constructs under the microfluidic device at a later stage.

## Discussion

Here, we develop a brain-mimetic 3D organoid culture platform by combining two basic tissue-engineering elements; matrix (human brain tissue-derived ECM) and bioreactor (microfluidic chamber device). This combination aims to solve problems of current organoid culturing, which have hindered the practical application of brain organoids for disease modeling and drug screening, by providing brain-specific ECM cues and a precisely controlled brain fluid-mimetic dynamic environment. The organoid culture with BEM-integrated microfluidics enables the crucial features of human brain development that have been limitedly demonstrated in the cerebral organoids previously reported. Our bioengineered human cerebral organoids exhibit structural, phenotypic, and functional features being observed during whole human brain development as follows; (1) the structural features of spontaneous brain morphogenesis (e.g. widespread corticogenesis, organization of highly complex structures with elongated cortical layers, apicobasal polarization of radial organization, preplate splitting), (2) mature neuronal identities (e.g. abundant expression of mature neuronal markers (NeuN, VGLUT1, GAD, GABA), extensive neural networks (N-cadherin), and synaptogenesis (SYNI, PSD95) at earlier time points), (3) divergent brain cell population (e.g. radial glial cells, microglia, astrocytes, excitatory/inhibitory neurons, Cajal-Retzius cells), and (4) electrophysiologically active properties (e.g. sodium current, multi-peaks of AP, postsynaptic current).

Although improvements in some of the cellular events and features related to whole brain development have been demonstrated in previously reported mature organoids engineered with several technologies, to the best of our knowledge, there have been few cerebral organoids simultaneously exhibiting all those characteristics. For example, genetic modification of PSCs increased NP proliferation and induced expansion and folding in cerebral organoids, but delayed neuronal differentiation^68^. Synthetic polymer fiber microfilaments generated cerebral organoids with more continuous neuroepithelium^20^. However, the maturity of engineered organoids was not confirmed in terms of mature neuronal marker expression, folding structure, and electrophysiological functionality. Moreover, the treatment of CHIR99021, an exogenous patterning factor, led to more reproducible forebrain formation rather than whole brain regions. As our organoids do not involve any small molecules and solely rely on spontaneous development in the presence of brain-specific ECMs, they resemble more closely to the whole brain development. Furthermore, the developmental processes were significantly accelerated in our cerebral organoids than in previously engineered organoids. In another study, supplementation of differentiation medium with brain-derived neurotrophic factor reduced cell death and improved cerebral organoid development for an extended period^19^. However, the expression of mature neuron and astrocyte markers (e.g. VGLUT1, vesicular GABA transporter, GFAP) appeared only at 6 months. The SYNI^+^ neurons began to be expressed at 3 months. A recent study also reported that the expression of mature neuronal markers, including VGLUT1 and GAD1, was observed in 6-month-old cerebral organoids^69^. In our study, such mature developmental markers GAD1, GABA, and VGLUT1 were expressed in the organoids at 45 days of culture (Supplementary Fig. 9d, h), implying that combination of BEM and microfluidics shortened the time for the appearance of excitatory and inhibitory neuronal populations. In addition, GFAP and SYNI were detected at 60 days of culture (Fig. 6o, p), indicating that glial differentiation and synaptogenesis which occur at a late state of brain development might be accelerated by BEM-incorporated microfluidics. Therefore, we believe that our engineered organoids show the positive expression for excitatory and inhibitory neurons, astrocytes, and synapses at earlier time points than cerebral organoids generated using previous technologies. The superiority of our organoids may lie on the ability to recapitulate more rapidly spontaneous development of whole human brain.

Early exposure of brain organoids to biochemical and biophysical cues provided by BEM facilitates the development of brain organoids. We found that like Mat, BEM hydrogel is retained for approximately 60~70 days of culture. The improvements in BEM organoids at late time points after BEM disappears are probably due to the prolonged effect of the signals from ECM given at earlier days of culture. Brain region-specific organoid development methods involve the use of patterning factors at an early stage to specify progenitor fate. These factors are then removed or minimized, and subsequent differentiation follows intrinsic programs. Likewise, biochemical and biophysical cues of ECM at early point seem to be critical because neuroepithelium expansion occurs and fate of progenitors are specified at an early stage of brain organoid development, which affects subsequent maturation stages. In the future study, it may be preferable to provide brain organoids with ECM microenvironments adjusted to developmental stages by applying fetal BEM and adult BEM at early and later time points of organoid culture, respectively. A recent study to compare porcine fetal and adult brain ECMs demonstrated that all types of ECM proteins were included in both ECMs, but fetal brain-derived ECM contained higher contents of fibrillin and biglycan^70^. Therefore, it would be important to investigate any changes in ECM compositions in-depth during brain development and reflect such ECM changes to different stages of brain organoid culture.

A fine-tuned consistent dynamic flow provided by the BEM-incorporated microfluidic device not only significantly reduced the formation of the necrotic region and decreased apoptotic cell death throughout the structures of cerebral organoids, but also affected metabolic states and enhanced cell proliferation. Although several methods have been applied to facilitate oxygen/nutrient supply and waste exchange and reduce necrotic region in organoids, our cerebral organoids engineered with BEM-microfluidic device underwent improved structural, phenotypic, and functional maturation compared to previously reported cerebral organoids. A recent study adapted organotypic slice culture at the air–liquid interface to cerebral organoids (ALI-CO), leading to improved neuronal survival and axon outgrowth^21^. However, because the organoids were cultured in slide sections, the whole structure as well as the cortical layers of brain organoid might not be well established. Another recent study generated vascularized human cortical organoids by ectopically expressing the vascular transcription factor in PSCs^71^. The presence of functional vascular-like networks in the organoids alleviated the apoptotic and hypoxic condition of the interior, and resulted in higher incidences of APs, but there was marginal improvement in cortical layer structure formation. This study examined maturity of vascularized cortical organoids by analyzing SYNI expression and incidence of AP. Our bioengineered organoids showed positive expression of SYNI at earlier time point (Fig. 6o) and higher incidence of APs (Fig. 7h). Miniaturized spinning bioreactors were developed to overcome some of the major limitations of cerebral organoids (*e.g*. large batch-to-batch variability, diffusional limitations of oxygen/nutrient, high cost, etc.)^8^, but the study did not examine synaptogenesis and the development of the organoids relied on region-specific differentiation protocols. Cerebral organoids generated by our methodology showed positive expression for mature synaptic markers (SYNI, PSD95) (Fig. 6o and Supplementary Fig. 16e). The presence of IBA1^+^ microglia (Supplementary Fig. 16a, b) indicates the diversity of cell population in our cerebral organoids. Importantly, brain organoids cultured in our microfluidic devices exhibited less variation in terms of size and gene expression levels (Fig. 5k and Supplementary Figs. 12a, 13e, f, 19, 20c, 22g) probably owing to uniform flow of medium to reduce variation in culture condition, demonstrating that microfluidic culture could improve the quality control of organoids. Although BEM enhanced overall growth, neuronal differentiation, cortical layer development, and electrophysiological function of brain organoids, BEM alone did not reduce the variation in the size of organoids in the orbital shaker without the use of microfluidic device (Figs. 2b and 4c). As BEM-incorporated microfluidics could generate cerebral organoids representing the development of the whole human brain with reduced variability, our cerebral organoids could satisfy both diversity and consistency in recapitulating brain development.

Due to the significant advantages of the microfluidic system as an in vitro culture platform, a couple of studies have previously demonstrated the application of chip systems for brain organoid cultures^22,23^. For example, the commercially available millifluidic system called Quasi Vivo increases oxygen transport, resulting in an increased proportion of dopaminergic neurons in midbrain organoids for up to 30 days of culture^23^. In another study, cerebral organoid culture using a 5-channel chip^23^ was applied in modeling neurodevelopmental disorders under chemical exposure^72^. However, the syringe pump-based perfusion method was employed for organoid culture in the chips, which decreases the throughput for culture and analysis due to a need for additional equipment. In addition, only short-term cultures were demonstrated in both studies where cortical layers and the radial organization of cell populations were not observed in the organoids. In contrast, our microfluidic chamber device was established as a simple-to-use, pump-free perfusion-based microfluidic culture platform. Cerebrospinal fluid-mimetic dynamic environments were achieved with a gravity-driven flow by laboratory rocker. Our system not only takes the beneficial features of microfluidic technologies, such as small-medium volumes, effective fluid exchanges, and precisely controlled fluid properties at the microscale, but also overcomes the limitations of current microfluidics for dynamic 3D cell cultures, including a low throughput in culture and analysis, and a requirement for complicated pumps, tubing, and expensive external devices.

Collectively, our organoid culture system based on combining material biology and microfabrication technology enables the generation of high-quality cerebral organoids with increased reproducibility, throughput with reduced cost, and feasibility for long-term 3D culture, providing a useful platform advancing the organoid models for studying human organogenesis and disorders. Our bioengineering strategy based on microfluidic brain matrix would be more effective for generating cerebral organoids that enable in vitro recapitulation of spontaneous whole brain development processes than previously reported technologies, while satisfying all important criteria of cerebral organoids, including diversity, maturity, and consistency. The maturation of cerebral organoids without involving exogenous morphogens and genetic alteration would be regarded as a significant advancement in cerebral organoid technology. As region-specific organoids generated by guided methods with supplementation of exogenous patterning factors have great potential for revealing the human-specific aspects of particular brain regions, our culture platform could be applied to modified cerebral organoid protocols with added exogenous factors or regional organoids to expand its applications for disease modeling and drug development in the future. It would also be broadly applicable to other types of 3D tissue organoid culture beyond the nervous system.

## Methods

### Preparation of the BEM

Human brain tissues were collected from the patients by excision surgery (Supplementary Table 1) and stored at −80 °C until use. For the use of human brain tissue for BEM preparation, informed consent was obtained from the participants and the study with human brain tissue was approved by the Institutional Review Board (IRB) (Permit Number: 4-2014-0769) of Yonsei University College of Medicine. Porcine brain tissues were obtained from the commercial market. The decellularization of the brain tissue was conducted following the protocol of our previous report^53^. The frozen brain tissues were slowly thawed, and dura mater was carefully removed. Then, brain tissues were cut into small pieces and agitated in buffer solutions in a consecutive order as follows: distilled water (24 h, 60 rpm), 0.05% (v/v) trypsin/ethylenediaminetetraacetic acid (trypsin/EDTA, #GIB-15400-054; Thermo Fisher Scientific, Waltham, MA, USA) (90 min at 37 °C, 60 rpm), 3% (v/v) Triton X-100 (#X100; Sigma-Aldrich, St. Louis, MO, USA) (120 min), 1 M sucrose solution (#84097; Sigma-Aldrich) (30 min), distilled water (15 min), 3% (v/w) sodium dodecyl sulfate (#71736; Sigma-Aldrich) (60 min, 150 rpm), 4% (v/v) ethanol (#E7023; Sigma-Aldrich) (120 min, 150 rpm), 1% (v/v) Triton X-100 (#X100; Sigma-Aldrich) with 0.1% (v/v) ammonium hydroxide (Sigma-Aldrich; #320145) (60 min), phosphate-buffered saline (PBS; Sigma-Aldrich) (15 min), 1% (v/v) penicillin/streptomycin (P/S, #GIB-15140-122; Thermo Fisher Scientific) (30 min, 60 rpm), distilled water (15 min, 90 rpm), and PBS (15 min, 90 rpm). After the detergent treatments, decellularized tissues were washed with distilled water thrice before preceding to the next step. All procedures were performed at 4 °C and agitated at 120 rpm if not stated specifically. The decellularized brain tissues were lyophilized and stored at 4 °C until use. BEM was prepared by solubilizing decellularized brain tissue with 4 mg/ml pepsin (#P7000; Sigma-Aldrich) in 0.02 M HCl and stirred at 120 rpm at room temperature for two days. For organoid culture, a BEM solution of 40 mg/ml (w/v) concentration was mixed with Matrigel (#354277; Corning Inc., Corning, NY, USA) to adjust the final concentration to 400 μg/ml (w/v) BEM.

### Fabrication of microfluidic device

The microfluidic device was fabricated by a standard soft lithography technique. The mixture of poly(dimethylsiloxane) (PDMS) pre-polymer solution (Dow Corning, Inc., Midland, MI, USA) and curing agent (Sylgard^®^ 184, Dow Corning, Inc.) at a ratio of 10:1 (v/v) was poured onto the patterned master wafer with a 2.2 mm thickness to mold the microfluidic devices as per the traditional replica molding process^53^. The details on the dimensions of the microfluidic device are provided in Supplementary Fig. 2a. An 8-mm diameter biopsy punch was used to punch holes into the chambers (Kai Industries Co., Ltd., Gifu, Japan). The samples were exposed to oxygen plasma (CUTE; Femto Science, Seoul, Korea) to bond the PDMS layers. Two layers of the patterned PDMS were stacked on top of each other, and a thin film of PDMS was bonded at the bottom as a seal. For sterilization, the assembled devices were autoclaved and dried under ultraviolet light before use.

### Human iPSC maintenance

Human iPSC lines WT3 (kindly provided by the Yonsei University School of Medicine) and KYOU-DXR0109B (#ACS-1023; American Type Culture Collection, Manassas, VA, USA) were used. The use of human iPSCs was approved by the Institutional Review Board (IRB) of Yonsei University (Permit Number: 1040917-201510-BR-229-01E, 7001988-202104-BR-1167-01E). The culture and maintenance of human iPSCs were conducted as previously reported^4,73^. Human iPSCs were cultured in iPSC-maintaining medium composed of Dulbecco’s Modified Eagle’s Medium/Nutrient Mixture F12 (DMEM/F12, #11320-082; Thermo Fisher Scientific), 20% (v/v) Knockout Serum Replacement (KSR, #10828-028; Thermo Fisher Scientific), 1% (v/v) penicillin-streptomycin (P/S, #GIB-15140-122; Thermo Fisher Scientific), 1× non-essential amino acid (NEAA, #GIB-11140-050; Thermo Fisher Scientific), 0.1 mM β-mercaptoethanol (#63689-25ML-F; Sigma-Aldrich), and 10 ng/ml basic fibroblast growth factor (bFGF, #4114-TC-01M; R&D System Inc., Minneapolis, MN, USA). The cells were maintained on the feeder cell layers of 10 μg/ml (w/v) mitomycin C (#M4287; Sigma-Aldrich)-treated STO fibroblasts (#CRL-1503; American Type Culture Collection) and sub-cultured every seven days using 2 mg/ml (w/v) collagenase type IV (#17104-019; Thermo Fisher Scientific). Human iPSCs were also maintained in feeder-free conditions on Matrigel-coated plates and cultured in mTeSR^TM^ Plus medium (#100-0276; STEMCELL Technologies, Vancouver, Canada).

### Generation of brain organoids

Brain organoids were generated and cultured with a slightly modified protocol from a previously described method by Lancaster *et al*.^3,4,20^. Human iPSCs were detached from STO feeder layers by 2 mg/ml collagenase type IV treatment for 30–45 min at 37 °C. Then, iPSC clumps were dissociated by Accutase (#A1110501; Thermo Fisher Scientific) to generate single cells. For human iPSCs cultured on Matrigel-coated plates, single-cell suspension was prepared by trypLE (#12604013; Thermo Fisher Scientific) treatment. EBs were generated by seeding 8,000 cells into a well of ultra-low-attachment 96-well plate (#CLS7007, Corning Inc.). EBs generated from iPSCs cultured on the feeder layer were cultured in iPSC-maintaining medium supplemented with 4 ng/ml bFGF (#4114-TC-01M; R&D System Inc.) and 50 μM Rho-associated protein kinase (ROCK) inhibitor (#Y0503; Sigma-Aldrich). EBs generated from feeder-free conditions were cultured in Essential 8 medium (Thermo Fisher Scientific) and 50 μM ROCK inhibitor. After 5–6 days of culture, the formed EBs were transferred into a low-attachment Petri dish (21.5 cm^2^, #10060; SPL Life Sciences, Pocheon, Korea). To induce the neuroepithelium-like structures, the EBs were cultured in suspension in a neural induction medium composed of DMEM/F12 (#11320-082; Thermo Fisher Scientific), 1× N2 supplement (#GIB-17502-048; Thermo Fisher Scientific), 1× Glutamax (#35050061; Thermo Fisher Scientific), 1× MEM-NEAA (#GIB-11140-050; Thermo Fisher Scientific), and 1 mg/ml heparin (#H3149-10KU; Sigma-Aldrich). The medium was exchanged daily for five days. At day 11, EBs with neuroepithelium identity were encapsulated in 30 μl of Matrigel (#354277; Corning Inc.) or BEM-supplemented Matrigel, and then transferred to a 24-well plate (#CT-3526; Corning Inc.) and were further cultured in a differentiation medium comprising DMEM/F12 (#11320-082; Thermo Fisher Scientific) and Neurobasal (#GIB-21103-049; Thermo Fisher Scientific) (1:1) ratio supplemented with 1:200 (v/v) N2 supplement (#17502-048; Thermo Fisher Scientific), 1:100 (v/v) B27 supplement without retinoic acid (#12587-010; Thermo Fisher Scientific), 1:100 (v/v) Glutamax (#35050061; Thermo Fisher Scientific), 1:200 (v/v) MEM-NEAA (#GIB-11140-050; Thermo Fisher Scientific), 1:4000 (v/v) insulin (#I9278; Sigma-Aldrich), and 3.5 μl/ml β-mercaptoethanol (#21985023; Sigma-Aldrich) for four days. From day 15, the organoids encapsulated in Matrigel or BEM hydrogel were cultured in a 60-mm dish on an orbital shaker (Mat organoid or BEM organoid, respectively) (#NBT101SRC; BioTek, Winooski, VT, USA). The organoids encapsulated in BEM hydrogel were also cultured in a well plate on a bi-directional rocker (BEM-plate organoid) (IKA, #26-01270-01; Staufen, Germany). The differentiation medium for the dynamic culture had the same composition, except for the B27 supplement with retinoic acid (#17504-044; Thermo Fisher Scientific). For the dynamic culture in the microfluidic device, a single organoid in BEM hydrogel was transferred into each organoid culture chamber (one organoid per chamber) in between the medium chambers (BEM-device organoid). The culture chambers were then covered with a PDMS lid and placed on a bi-directional rocker. Two organoids were cultured per device.

Microfilament-engineered cerebral organoids (enCORs) were generated as previously reported^20,74^. In brief, coated VICRYL (polyglactin 910) suture (size 5-0, Ethicon, Cincinnati, OH, USA) was mechanically sheared using a scalpel and cut into 1-mm pieces. At day 13 (2 days after encapsulation into Matrigel or BEM hydrogel), neural induction medium was replaced with differentiation medium. CHIR99021 (3 μM, #C6556; LC Laboratory, Woburn, MA, USA) was added into culture medium from day 13 to 15. For dynamic culture, enCORs in the dish were moved onto an orbital shaker or enCORs in the microfluidic device were placed on a rocking shaker.

### Characterization of the BEM

Histological analysis of decellularized human brain tissue was performed to confirm the removal of cellular components and the preservation of ECM components. Decellularized human brain tissues were embedded in the OCT compound (#HCP-0100-00A; CellPath, Hemel, Hempstead, UK), frozen at −80 °C, and sectioned to 6 μm thickness. Hematoxylin and eosin (H&E) and Masson’s Trichrome (MT) staining of the sectioned tissues were conducted to identify the removal of cells and the presence of collagen, respectively. To further verify the removal of cellular components, DNA was quantified in brain tissue before and after decellularization. The total DNA was isolated using a DNA extraction kit (#K-3032; Bioneer, Daejeon, Korea), according to the manufacturer’s instructions, and the DNA content of each sample was determined by measuring its absorbance at 260 nm on a microplate reader (Infinite M200 Pro, Tecan, Maennedorf, Switzerland). The GAG content was measured in the native brain and decellularized brain tissues using 1,9-dimethyl methylene blue dye solution (#341088; Sigma-Aldrich, St. Louis, MO, USA) and chondroitin sulfate A (#C9819; Sigma-Aldrich) as a standard, as previously described^53^.

### Proteomic analysis

Proteomic analysis was performed on total 12 samples of Matrigel (*n* = 1, biological replicate = 1), human BEM (*n* = 3, biological replicates = 3), pBEM derived from porcine cortex (*n* = 4, biological replicates = 4), and pBEM derived from porcine cerebellum (*n* = 4, biological replicates = 4). Proteomic analysis was performed using A Thermo Scientific Q Exactive Hybrid Quadrupole-Orbitrap equipped with Dionex U 3000 RSLCnano HPLC system (Thermo Fisher Scientific). The purified peptide sample was reconstituted in solvent A (Water/Acetonitrile (98:2 v/v), 0.1% Formic acid), and then injected into LC-nano ESI-MS/MS system. The sample was trapped in an Acclaim PepMap 100 trap column (100 μm × 2 cm, nanoViper C18, 5 μm, 100 Å, Thermo Fisher Scientific) and washed for 6 min with 98% solvent A at a flow rate of 4 μl/minute, and then separated on an Acclaim PepMap 100 capillary column (75 µm × 15 cm, nanoViper C18, 3 µm, 100 Å, Thermo Fisher Scientific) at a flow rate of 300 nl/minute. The LC gradient was run at 2 to 35% solvent B over 30 min, then from 35 to 90% over 10 min, followed by 90% solvent B for 5 min, and finally 5% solvent B for 15 min. Xcaliber software version 3.1 was used to collect MS data. The Orbitrap analyzer scanned precursor ions with a mass range of 350–1800 *m/z* with 70,000 resolution at *m/z* 200. For collision-induced dissociation, up to the 15 most abundant precursor ions were selected. The normalized collision energy was 32. Mass data were acquired automatically using proteome discoverer 2.2 (Thermo Fisher Scientific).

Protein identification was performed by Thermo Proteome Discoverer (version 2.4.1.15). Proteins were identified by searching MS and MS/MS data of peptides against the *Homo sapiens* UniProt database (2020.10 release) for human BEM, *Sus scrofa* UniProt database (2020.12 release) for pBEM, and *Mus musculus* UniProt database (2020.12 release) for Matrigel. Trypsin was used as the protease for cleavage and up to two missed cleavages were allowed. Carbamidomethylation of cysteines was set as a static modification. Oxidation of methionine, N-terminal acetylation, and N-terminal methionine excision were set as dynamic modifications. Precursor mass tolerance was set to 10 ppm and fragment mass tolerance was set to 0.02 Da. Proteins and peptides were filtered for false discovery rate (FDR) less than 1%.

We compared the identified proteins in Matrigel and human BEM with an Atlas of human brain-expressed genes^75^. The contents and composition of matrisome proteins in Matrigel, human BEM, pBEM, and human brain tissue^34^ were compared based on relative intensity-based absolute quantification (riBAQ) values, which are approximately proportional to the molar amount of proteins present in the matrices. Gene ontology biological process (GOBP) analysis^76–78^ was performed on the proteins identified in BEM, but absent in Matrigel, which have at least four-fold elevation of expression in human brain tissue compared to other tissues^35,78,79^. For statistical significance, proteins and peptides were filtered for FDR and *p* value less than 0.05. Raw *p* value was determined by Fisher’s exact test.

### Histology and immunohistochemistry

At several time points in the organoid culture (30, 45, 60, 75, 100, and 120 days), the brain organoids were fixed in 10% formalin solution (#HT501640; Sigma-Aldrich) for one hour at room temperature, washed twice using phosphate-buffered saline (PBS, Biosesang, Seongnam, Korea), and immersed in 30% (w/v) sucrose dissolved in distilled water overnight at 4 °C for cryoprotection. The organoids were embedded in OCT compound and frozen at −80 °C. The samples were then sectioned in tissue slices of 20 μm thickness using cryostat (Leica Inc., Wetzlar, Germany). For the histological analysis, the tissue sections were examined with H&E staining to analyze the overall cellular morphology and cavity in the organoids. For immunohistochemical staining, the sectioned tissues were washed with PBS to remove excess OCT and permeabilized with 0.2% (v/v) Triton X-100 (#X100; Sigma-Aldrich) in PBS for 20 min. Then, the sections were treated with 4% (w/v) bovine serum albumin (#216006980; MP Biomedicals, Santa Ana, CA, USA) and 2% (v/v) horse serum (#16050130; Thermo Fisher Scientific) for one hour to block the non-specific binding of antibodies. The samples were incubated with primary antibodies listed in Supplementary Table 6. The stained samples were then washed with PBS three times and incubated with Alexa Fluor 488 or 594-conjugated secondary antibodies (1:200; Thermo Fisher Scientific) for signal visualization. The nuclei were counterstained with 4′,6-diamidino-2-phenylindole (DAPI, #A2412; TCI America, Portland, OR, USA) for 30 min and washed with PBS. The samples were mounted using a fluorescent mounting medium (#H1400; Vector laboratories, Burlingame, CA, USA) and observed under a confocal microscope (LSM 880, Carl Zeiss, Oberkochen, Germany).

### Image-based quantification

Fluorescence intensities of Tuj1 and MAP2 in the whole section of organoids were measured by selecting the marker positive area and using ‘Integrated density’ function in ‘set measurements’ setting in ImageJ. The corrected total organoid fluorescence was calculated by the following equation; Integrated density – (Area of selected cell × Mean fluorescence of background readings). The area and perimeter of PAX6^+^ ventricle-like structures from 4 representative organoids were measured using ImageJ. The average number of cells positive for SOX2, NeuN, TBR2, HOPX, IBA1, and CD68 per field was manually counted using ImageJ. The average percentage ratio of cells positive for SOX2 per field was quantified by manually counting the positively stained cells and dividing them by the number of DAPI-stained nuclei. The positive areas for EthD1, Ki67, FOXG1, and NeuN were determined by measuring the area stained positively for markers and normalized to DAPI^+^ area using Image J. For Nestin^+^, cCASP3^+^, HIF-1α^+^, VGLUT1^+^ area measurements, the area positively stained for each marker was normalized to the total area of the image. The thickness of the cortical plate, defined as the densely packed CTIP2^+^ regions, and the superficial layer defined as the SATB2^+^ regions were measured using ImageJ. Three measurements were taken at 45-degree angles to obtain the mean value.

### qPCR analysis

The gene expression of the brain organoids at 30 and 75 days of culturing was investigated using qPCR analysis, as previously described^53,73^. Total RNA was isolated from approximately ten brain organoids per sample using an RNA extraction kit (#9767 A; Takara Bio lnc., Kusatsu, Shiga, Japan) following the manufacturer’s instructions. The RNA concentration was calculated by measuring the absorbance of the samples at 260 nm using a spectrophotometer (Infinite M200 Pro, Tecan). The total RNA was reverse-transcribed into cDNA using a TaKaRa PrimeScript II First-strand cDNA synthesis kit (#6110 A; TaKaRa Bio lnc.). The synthesized cDNA was used for the qPCR reaction with the TaqMan Fast Universal PCR MasterMix (#4366073; Thermo Fisher Scientific). The gene expression was quantified with the Step One Plus Real-Time PCR system (Applied Biosystems, Foster City, CA, USA) for the targets listed in Supplementary Table 7. TaqMan Assay primers were purchased from Thermo Fisher Scientific. The relative gene expression of each target was calculated by the comparative C_t_ method and normalized using an endogenous reference glyceraldehyde 3-phosphate dehydrogenase (*GAPDH*). The gene expressions of the human brain organoids were compared with human neural stem cells (NSCs, provided by Prof. Kook In Park at Yonsei University College of Medicine) isolated from the brain tissue of a 13-week fetus with full written parental consent and approval of the research ethics committee of Yonsei University College of Medicine (protocol #4-2003-0078).

### Tissue-specific ECM experiment

To investigate the tissue-specific effect of ECM on brain organoid development, decellularized ECM derived from porcine organs, including the brain (BEM), intestines (IEM), liver (LEM), and heart (HEM), was tested for brain organoid culture. Decellularization of the porcine liver and heart was performed as previously reported^56^. The porcine small intestine was decellularized as follows: the intestine was thoroughly washed and cut laterally alongside the intestinal wall so that it was in a flat, rectangular form. Then, it was cut into segments of 20 cm in length and agitated in the following solutions for the time indicated: distilled water (24 h), decellularizing solution containing 1% (v/v) Triton X-100 with 0.1% (v/v) ammonium hydroxide (#320145; Sigma-Aldrich) (48 h), distilled water (24 h), 1% (v/v) penicillin/streptomycin (#GIB-15140-122; Thermo Fisher Scientific) (1 h), and distilled water (1 h). All processes were performed at 4 °C and agitated at 120 rpm. The decellularized intestine tissues were lyophilized and stored at 4 °C until use. The porcine brain was decellularized using the same protocol for the human brain. At day 11, the EBs with neuroepithelium identity were encapsulated in 30 μl Matrigel (#354277; Corning Inc., Corning, NY) or decellularized ECM gel (0.4 mg/ml ECM + Matrigel) and cultured in 24-well plates (Corning Inc.) in differentiation medium for four days. Then, 24-well plates containing organoids were placed on a bi-directional rocker (#26-01270-01; IKA, Staufen, Germany) for dynamic culture. At 30 days of culture, brain organoids were analyzed by light microscope observation, immunostaining, and qPCR.

### Calcium imaging

To examine the response of brain organoids to neurotransmitters, the organoids were incubated with the intracellular calcium indicator 2 μM Fluo-4 AM (#F14201; Thermo Fisher Scientific) for 40 min at 37 °C and additionally for 30 min at 25 °C. Time-lapse changes of intracellular Ca^2+^ levels in brain organoids were imaged after treatment with 100 μM glutamate (Sigma-Aldrich) or 50 μM GABA (#A2129; Sigma-Aldrich). The cells in the treated organoids were imaged every 3.725 s using a confocal microscope (LSM 880, Carl Zeiss) at room temperature. The ZEN software (Carl Zeiss) was used to determine the changes in the intensity of fluorescence. Briefly, the ROI was drawn around individual cells identified by their morphology. The Δ*F*_*1*_/*F*_*0*_ trace for each ROI was calculated by dividing the time-varying fluorescence by the initial baseline fluorescence.

### Synthesis and spectral characterizations of oxygen-sensing nanoparticles

First, an amphiphilic precursor, urethane acrylate nonionomer (UAN), was synthesized as reported previously^60^. The UAN precursor consisted of hydrophilic polyethylene oxide (PEO; MW 1,500 g/mol) and hydrophobic polypropylene oxide (PPO; MW 700 g/mol) segments that were linked covalently in a T-shape. To carry out the polymerization of the UAN precursors via a radical reaction among their vinyl groups, 10 g of UAN was dissolved in dimethyl sulfoxide (DMSO) to obtain a homogeneous UAN-DMSO solution. After adding 0.02 g of azobisisobutyronitrile (AIBN), a radical initiator, the temperature of the solution was slowly increased to 60 °C and maintained for three hours with gentle stirring. Polymerized UAN-DMSO solution was named as poly(UAN) (PUAN)-DMSO solution. Then, 5.5 mg of the oxygen-sensitive phosphor, Pt(II) meso-tetra(pentafluorophenyl)porphine (PtTFPP; Frontier Scientific, Inc.), was added to 1.0 mL of PUAN-DMSO solution which was further diluted with DMSO to form a stock solution at a concentration of 1860 ppm. PtTFPP-PUAN nanoparticles were formed via the nano-precipitation method^80^. Briefly, the stock solution of PtTFPP-PUAN was dropped very slowly into PBS with vigorous stirring at a volumetric 1:10 mixing ratio of PtTFPP-PUAN solution and PBS. Because PtTFPP was immiscible in the aqueous phase, the oxygen-sensitive phosphor remained partitioned within the hydrophobic core of PUAN nanoparticles^60^ in the course of formation of micelle-like PUAN nanoparticles dispersed in the aqueous PBS solution. The PtTFPP-PUAN stock solution in DMSO can be stored at room temperature before forming nanoparticle suspensions.

To identify the excitation wavelength for the oxygen-sensing nanoparticles, absorption was measured by exciting the PtTFPP-PUAN nanoparticle suspension from 300 to 600 nm under both normoxic and deoxygenated conditions. Gentle bubbling with N_2_ gas into the nanoparticle suspension (<1 ml) contained in a 15 ml conical tube for 30 min allowed for complete deoxygenation of the nanoparticle suspension. Immediately after N_2_-bubbling, the deoxygenated nanoparticle suspension was transferred into a 96-well plate (50 μl/well), and excitation spectra were acquired at room temperature using a microplate reader (Infinite 200 Pro, Tecan). Similarly, the emission spectra under the normoxic and deoxygenated conditions were also obtained by measuring the phosphorescence intensity of the oxygen-sensing nanoparticles from 600 to 850 nm with 430 nm as a fixed excitation wavelength. Based on the Stern–Volmer equation, the relative phosphorescence intensity, which is represented by phosphorescence under the deoxygenated condition (*I*_0_) divided by phosphorescence (*I*), is linearly proportional to oxygen (i.e. quencher) concentration^60^.

### Measurement of oxygen levels in brain organoids

To monitor the oxygen levels within organoids, the oxygen-sensing nanoparticles were allowed to diffuse into the brain organoids by incubating the organoids in the medium containing the PtTFPP-PUAN nanoparticles at 37 °C overnight. The stock PtTFPP-PUAN solution in DMSO was pre-mixed with the culture medium at a ratio of 1:1 (v/v). Then, nanoparticle-incorporated brain organoids were rinsed with Dulbecco’s PBS (#D5652; Sigma-Aldrich) before analysis. The working principle of the optical measurement of the oxygen level is as follows. Upon photoexcitation of the oxygen-sensitive phosphor (i.e. PtTFPP) encapsulated within the PUAN nanoparticles at 430 nm, electrons in PtTFPP become excited in the triplet state. As these electrons in the excited triplet state fall into the ground singlet state, PtTFPP emits phosphorescence. When oxygen molecules are abundant around the excited PtTFPP (*e.g*. high oxygen concentration), random collisions between the oxygen molecules and the excited PtTFPP lead to energy transfer to oxygen; thus, such PtTFPP is no longer capable of emitting phosphorescence. This process is called dynamic quenching, which explains why high oxygen levels result in low phosphorescence intensity. The phosphorescence of PtTFPP-PUAN nanoparticles incorporated within the brain organoids was observed at 754 nm while exciting at 430 nm on a laser scanning confocal microscope (LSM 880, Carl Zeiss). The ZEN microscope software was used to measure the intensity of phosphorescence signals. The mean phosphorescence intensity of the BEM-device group was normalized to that of the BEM-plate group using Zen software.

### Tissue clearing and imaging of whole 3D brain organoids

Brain organoids were cleared using Clear, Unobstructed Brain Imaging Cocktails, and Computational analysis (CUBIC) using the following protocol^81^; the organoids were incubated with 4% paraformaldehyde (Sigma-Aldrich) at 4 °C overnight. The fixed samples were then immersed in modified ScaleCUBIC-1 solution consisting of 10% (w/w) Triton X-100, 5% (w/w) NNNN-tetrakis (2-HP) ethylenediamine, 10% (w/w) urea, and distilled water for three days at 37 °C, and washed with PBS three times at room temperature. Cleared brain organoid samples were analyzed by immunohistochemical staining for confocal microscopy imaging. The samples were incubated with primary antibodies for three days at room temperature, washed at least three times with PBS, incubated with secondary antibodies labeled with Alexa Fluor-conjugated 488 or 594 for two days at room temperature, and counterstained with DAPI for nucleus staining. For light-sheet microscopic imaging, the nuclei of the samples were stained with Syto16 (Thermo Fisher Scientific). The samples were incubated in ScaleCUBIC-2 solution [25% (w/w) urea, 50% (w/w) sucrose, 10% (w/w) triethanolamine, and distilled water] for 24 h to improve transparency, mounted with the same solution, and visualized on a confocal microscope (LSM 880, Carl Zeiss and Ni-E upright microscope +A1, Nikon, Tokyo, Japan) or light-sheet microscope (Zeiss Z1, Carl Zeiss). The 3D reconstruction and analysis of the images were performed using the IMARIS program (version 9.2, Oxford Instruments, Abingdon, UK). To visualize the 3D surface of brain organoids, 3D stack images were modified using the normal shading view. The location of TBR1^+^ cells was analyzed using a spot tool. The volume and sphericity of the organoids were measured using a surface tool

### Electrophysiology

The whole-cell patch-clamp recording was performed as previously described^10^. During recording, brain organoids were submerged and continuously perfused with an artificial cerebrospinal fluid (aCSF) consisting of (in mM): 119 NaCl, 2.5 KCl, 11 glucose, 26 NaHCO_3_, 1.25 NaH_2_PO_4_, 2.5 CaCl_2_, and 1.3 MgCl_2_ saturated with 95% O_2_ and 5% CO_2_ at 30–32 °C. Recording glass pipettes (2–4 MΩ) were filled with an intracellular solution containing (in mM): 135 potassium methanesulfonate, 10 KCl, 10 HEPES, 1 EGTA, and 2 Na_2_ATP (pH 7.2 to 7.4 adjusted with KOH). The whole-cell recordings were performed using an upright microscope (BX51WI, Olympus, Tokyo, Japan) with IR-DIC optics. Signals were recorded using a MultiClamp 700B amplifier, filtered at 3 kHz using a Bessel filter, and digitized at 10 kHz with a Digidata 1550B analog-to-digital board (Molecular Devices, Sunnyvale, CA, USA). To measure Na^+^ currents, cells were subjected to a series of voltage steps (1000 msec duration) from −30 to +20 mV (in 10 mV increments) at a holding potential of −60 mV in voltage-clamp mode. We measured the resting membrane potential, and then a series of current pulses (500 msec) with increasing amplitude between −5 pA and +25 pA (in 5 pA steps) were applied to evoke APs. The AP threshold was defined as the somatic voltage at which d*V*/d*t* exceeds 20 V/s. Synaptic currents were acquired from a neuron at a resting membrane potential (−60 mV).

### RNA-sequencing analysis

Brain organoids in each condition (Mat-plate, BEM-plate, BEM-device) were collected at the indicated time points (*n* = 3). For sample preparation, 5~10 organoids were collected from the 3D matrix per one batch of samples for each experimental condition. RNA was isolated using an RNA extraction kit (Takara Bio Inc.) according to the manufacturer’s instructions. RNA concentrations were measured using a NanoDrop8000 spectrophotometer (#ND-8000-GL; Thermo Fisher Scientific), and total RNA integrity was checked using an Agilent Technologies 2100 Bioanalyzer (#G2939BA; Agilent, Santa Clara, CA, USA). RNA was enriched for mRNA sequencing using an Illumina Truseq stranded mRNA library prep kit (#20020595; Illumina, San Diego, CA, USA). mRNA was enriched by poly-T oligo-attached magnetic beads (Truseq stranded mRNA library prep kit, Illumina) with two rounds of the purification process and fragmented from at least 1 μg of total RNA. Fragments of cleaved RNA which were primed with random hexamers were reverse-transcribed into the first strand of cDNA using reverse transcriptase (SuperScript™ II Reverse Transcriptase, #18064014; Thermo Fisher Scientific) in the presence of random primers. Afterward, a single ‘A’ nucleotide was attached to the 3′ ends of the cDNA fragments, and the adapter was subsequently ligated. To create the final strand-specific cDNA library, the samples were purified and amplified by PCR. The quality of the enriched cDNA libraries was verified by capillary electrophoresis (Bioanalyzer, Agilent). Subsequently, cDNA libraries were combined with a SYBR Green PCR MasterMix system (#4364346; Applied Biosystems) to present each value at equimolar concentration in the pool. A cBot 2 automated system (#SY-312-2001; Illumina) was used for the cluster generation of barcoded samples, which were applied for sequencing by synthesis on a HISEQ 2500 sequencing system (Illumina) with 100 bp length. The reads were aligned with Tophat 13 (v2.0.13; http://ccb.jhu.edu/software/tophat/) to map the reads and to calculate the expression levels between samples and a reference genome (human genome: hg19). To observe the differentially expressed genes (DEGs), aligned reads were analyzed by Cuffdiff (v2.2.0; http://cole-trapnell-lab.github.io/cufflinks/cuffdiff/). Then, to visualize RNA-sequencing data, Cuffdiff data were loaded to CummeRbund (v2.8.2; http://compbio.mit.edu/cummeRbund/manual_2_0.html). Aligned reads of the control and cases were subjected to fragments per kilobase of transcript per million (FPKM) estimation with Cufflinks (v1.3.0).

For the comparison of organoids in several brain regions (dorsolateral prefrontal cortex, ventrolateral prefrontal cortex, orbital frontal cortex, cerebellar cortex, hippocampus, cerebellum, striatum, and mediodorsal nucleus of the thalamus), the FPKM expression values of Brainspan were downloaded from the Allen BrainSpan human transcriptome dataset (http://www.brainspan.org/static/download.html). FPKM values were filtered for differential expression and joined with the Brainspan via the gene symbols. The similarity of expression was compared by the rank of the expressed gene via Pearson’s correlation. Pearson’s correlation coefficients between brain organoid samples and brain regions from fetal to adult stages were expressed as a heatmap.

GO term enrichment analysis was performed on genes with adjusted *p*-values < 0.05 that were considered to be significantly differentially expressed in comparison to the control group using the DAVID program (http://david.abcc.ncifcrf.gov) to obtain a comprehensive set of functional annotation.

GSEA was performed using brain tissue-related gene lists^19,82–87^ against normalized RNA-seq reads of Mat and BEM organoids samples with gene sets of 15 to 500 components included in the analysis using Broad Institute GSEA software. The weighted scoring scheme with a default parameter of other criteria was applied, and filtered gene sets were significantly enriched (*p-*value < 0.01).

To find disease-related term enrichment, the DAVID algorithm with GAD (Genetic Association Database) DISEASE term was utilized. Based on the upregulated gene list between the BEM-plate versus BEM-device organoid datasets with *P*-values < 0.05, the results of the functional annotation chart were checked and the GAD DISEASE related term with *P*-values < 0.01 was observed.

To check the overlap of differentially expressed genes among brain organoids under different culture conditions with known disease-related genes, BrainSpan datasets were utilized. The statistical significance of the overlap between the two groups of genes was calculated with Perl scripts in the online tool (http://nemates.org/MA/progs/overlap_stats.html). Disease-related terms showing significant overlapping with upregulated or downregulated gene lists in the BEM-plate versus BEM-device group datasets were the primary focus.

### Computational simulation of glucose concentration

A computational program was applied to simulate the glucose levels in brain organoids grown in the plate and microfluidic device. The model simulated the coupled convection‐diffusion and single‐phase flow, governed by Brinkman equations and Navier–Stokes equations. All simulations were performed using a commercial finite element solver in COMSOL Multiphysics software (COMSOL Inc., Burlington, MA, USA). The factors used in this simulation are listed in Supplementary Table 5. To simulate the gravity‐driven flow generated by the axial rocker, the boundary condition of the inlet was set to be sinusoidal pressure deriving back and forth flow with a maximum flow rate of 1.63 µl s^−1^. Since the microfluidic device has a symmetrical design, only half of the device with a symmetry boundary on its axis was simulated.

### Glucose and lactate measurements

Quantification of glucose level in brain organoids and lactate level secreted from organoids to culture medium was performed using a glucose assay kit (Abcam) and a lactate assay kit (Abcam), respectively, according to the manufacturer’s instructions.

### Necrotic area measurement

To compare necrotic area in brain organoids cultured in different conditions, the cells in organoids were exposed to ethidium homodimer-1 (Thermo Fisher Scientific) for 1 h at 37 °C and fixed in 10% formalin solution for 1 h. The samples were then incubated with DAPI overnight at 4 °C. The signals of dead cells and nuclear stain were imaged using a confocal microscopy (LSM 880).

### Statistical analysis

Quantitative data are described as mean ± standard deviation (SD) for *n* ≥ 3. Statistical significance between the control and experimental groups was determined as previously described^53,73^. Statistical analysis was performed using the two-tailed unpaired Student’s *t* test using GraphPad Prism 8 software (GraphPad Software, San Diego, CA, USA). *P* values lower than 0.05 were considered statistically significant.

### Reporting summary

Further information on research design is available in the Nature Research Reporting Summary linked to this article.

## Supplementary information

Supplementary Information

Description of Additional Supplementary Files

Supplementary Video 1

Supplementary Video 2

Supplementary Video 3

Supplementary Video 4

Reporting Summary

## Data Availability

The mass spectrometry proteomics data generated in this study have been deposited in the ProteomeXchange Consortium via the PRIDE partner repository^88^ with the dataset identifier “PXD025397”. The RNA-sequencing data generated in this study are available in the NCBI Gene Expression Omnibus (GEO) under accession code “GSE145386”. Source data are provided with this paper. Raw data for all figures and *p* values for supplementary figures are provided in Source Data file. Lists of total proteins and proteins that are known to be enriched in human brain tissues in all samples identified by proteomic analysis are also provided in Source Data file. The reference datasets of brain regions used for comparison are available in the Allen BrainSpan human transcriptome dataset [www.brainspan.org/static/download.html]. The protein samples were identified by searching MS and MS/MS data of peptides against the *homo sapiens* UniProt database (2020.10 release) for human BEM, *Sus scrofa* UniProt database (2020.12 release) for pBEM, and *Mus musculus* UniProt database (2020.12 release) for Mat. Proteins identified in Mat and human BEM were compared with the datasets in the Human Protein Atlas portal [www.proteinatlas.org]. The contents and composition of matrisome proteins in human brain tissues were analyzed from the data in the Human Proteome Map [www.humanproteomemap.org]. All datasets generated during the current study are available from the corresponding author on reasonable request. Source data are provided with this paper.

## Source data

Source Data
