# Supplementary material for: Microfluidic device with brain extracellular matrix promotes structural and functional maturation of human brain organoids
Source: Nat Commun. 2021 Aug 5;12:4730. doi: 10.1038/s41467-021-24775-5 (PMC8342542; doi:10.1038/s41467-021-24775-5)
Supplement: Supplementary file 1 — Supplementary Information [file 41467_2021_24775_MOESM1_ESM.docx]

**Microfluidic device with brain extracellular matrix promotes structural and functional maturation of human brain organoids**

Ann-Na Cho^1†^, Yoonhee Jin^1†^, Yeonjoo An^1†^, Jin Kim^1^, Yi Sun Choi^1^, Jung Seung Lee^1^, Junghoon Kim^1^, Won-Young Choi^2^, Dong-Jun Koo^3^, Weonjin Yu^4^, Gyeong-Eon Chang^1^, Dong-Yoon Kim^3^, Sung-Hyun Jo^5^, Jihun Kim^6^, Sung-Yon Kim^3,7^, Yun-Gon Kim^5^, Ju Young Kim^8^, Nakwon Choi^9^, Eunji Cheong^1^, Young-Joon Kim^2^, Hyunsoo Shawn Je^4^, Hoon-Chul Kang^6^, Seung-Woo Cho^1,10,11*^

^1^ Department of Biotechnology, Yonsei University, Seoul 03722, Republic of Korea

^2^ Department of Biochemistry, Yonsei University, Seoul 03722, Republic of Korea

^3^ Institute of Molecular Biology and Genetics, Seoul National University, Seoul 08826, Republic of Korea

^4^ Signature Program in Neuroscience and Behavioral Disorders, Duke-NUS Medical School, Singapore 169857, Singapore

^5^ Department of Chemical Engineering, Soongsil University, Seoul 06978, Republic of Korea

^6^ Division of Pediatric Neurology, Department of Pediatrics, Severance Children’s Hospital, Yonsei University College of Medicine, Seoul 03722, Republic of Korea

^7^ Department of Chemistry, Seoul National University, Seoul 08826, Republic of Korea

^8^ Department of Advanced Materials Engineering, Kangwon National University, Samcheok 25913, Republic of Korea

^9^ Brain Science Institute, Korea Institute of Science and Technology (KIST), Seoul 02792, Republic of Korea

^10^ Center for Nanomedicine, Institute for Basic science (IBS), Seoul 03722, Republic of Korea

^11^ Graduate Program of Nano Biomedical Engineering (NanoBME), Advanced Science Institute, Yonsei University, Seoul 03722, Republic of Korea

^†^ These authors contributed equally to this work.

***Corresponding author**

Prof. Seung-Woo Cho

Department of Biotechnology, Yonsei University, 50 Yonsei-ro, Seodaemun-gu, Seoul 03722, Republic of Korea. E-mail: [seungwoocho@yonsei.ac.kr](mailto:seungwoocho@yonsei.ac.kr)

**Supplementary Figures**

**
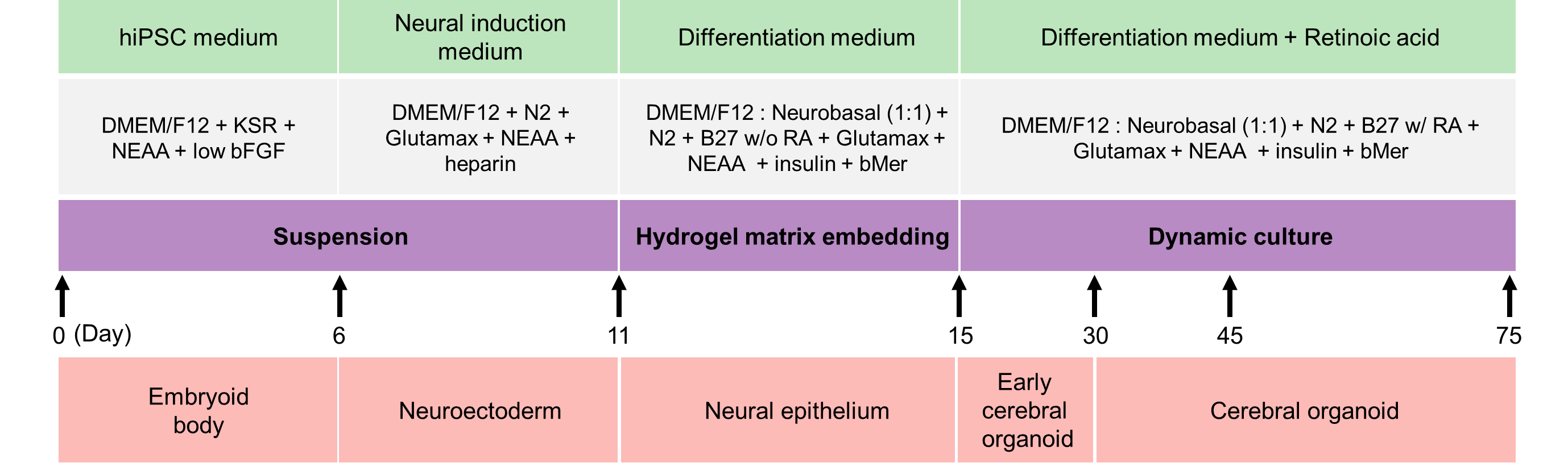
**

**Supplementary Figure 1. The protocol for human brain organoid culture using the microfluidic 3D brain extracellular matrix (BEM) system.**

**
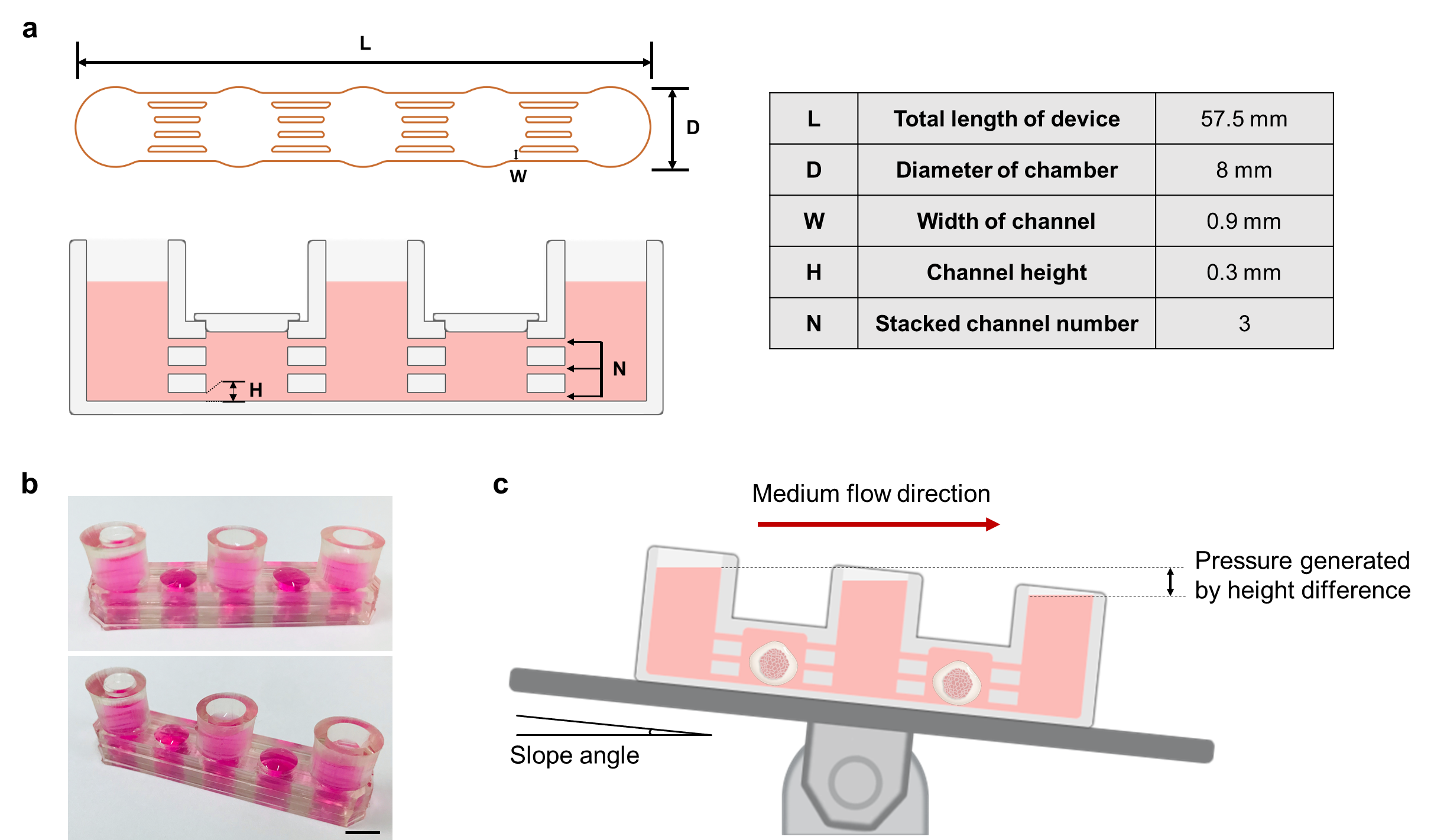
**

**Supplementary Figure 2**. **Design of a pump-free microfluidic device.** (**a**) Schematic diagram depicting the top and cross-sectional views of the microfluidic device and dimensions of each part. (**b**) Pictures depicting the side and top views of the microfluidic device with cultivation chambers interconnected by micron-sized channels (scale bar = 1 cm). (**c**) Principle of generating rocker system-driven medium flow in the chamber of the microfluidic device.

**
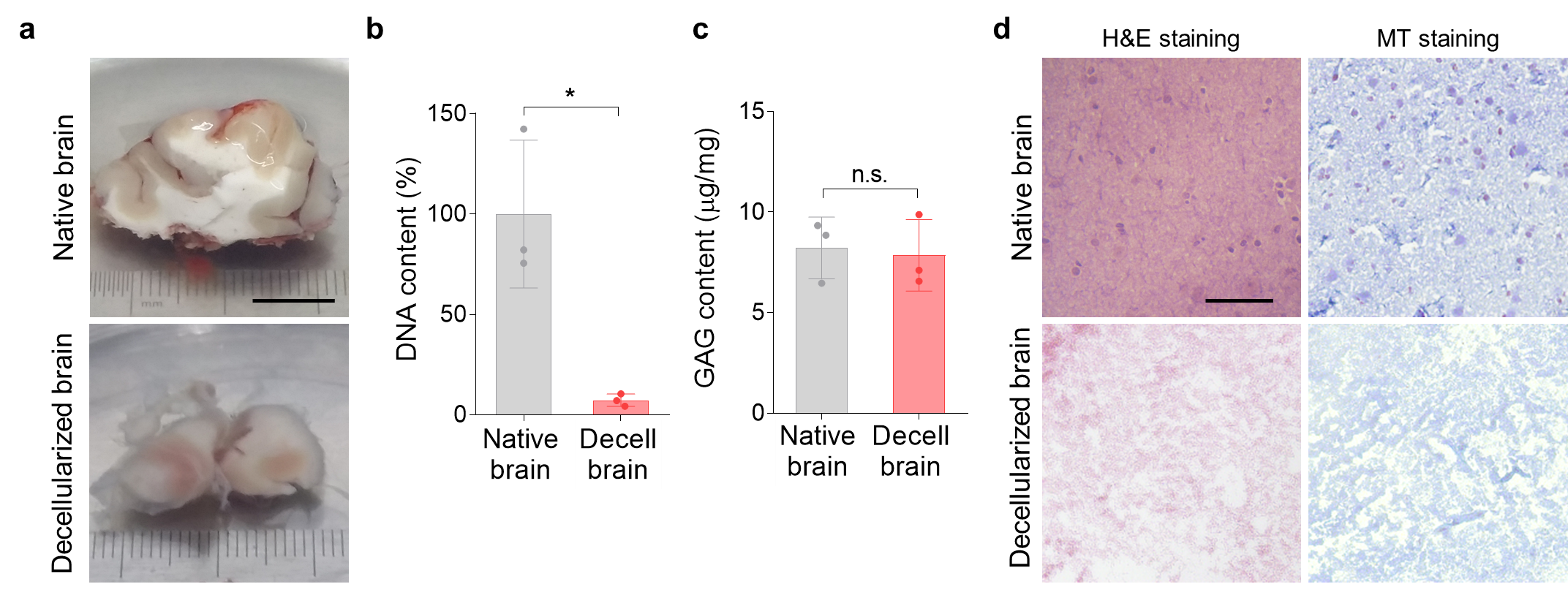
**

**Supplementary Figure 3. Characterization of decellularized human brain tissue.** (**a**) Gross view of the human brain tissue before and after the decellularization process (scale bar = 1 mm). Measurements of (**b**) DNA and (**c**) glycosaminoglycan (GAG) contents in the human brain tissue before and after decellularization (*n* = 3 per group, Native versus Decell *p* = 0.0122, n.s. = not significant). (**d**) Histological analyses of the native and decellularized brain tissues by Hematoxylin & Eosin (H&E) staining and Masson’s Trichrome (MT) staining showing the removal of cellular components and preservation of collagen, respectively (scale bar = 500 μm). Independent replicates = 3. All data are expressed as mean ± standard deviation (SD). Statistical differences between the groups were determined with unpaired two-tailed *t*-test (**p* < 0.05). Source data are provided as a Source Data file.

**
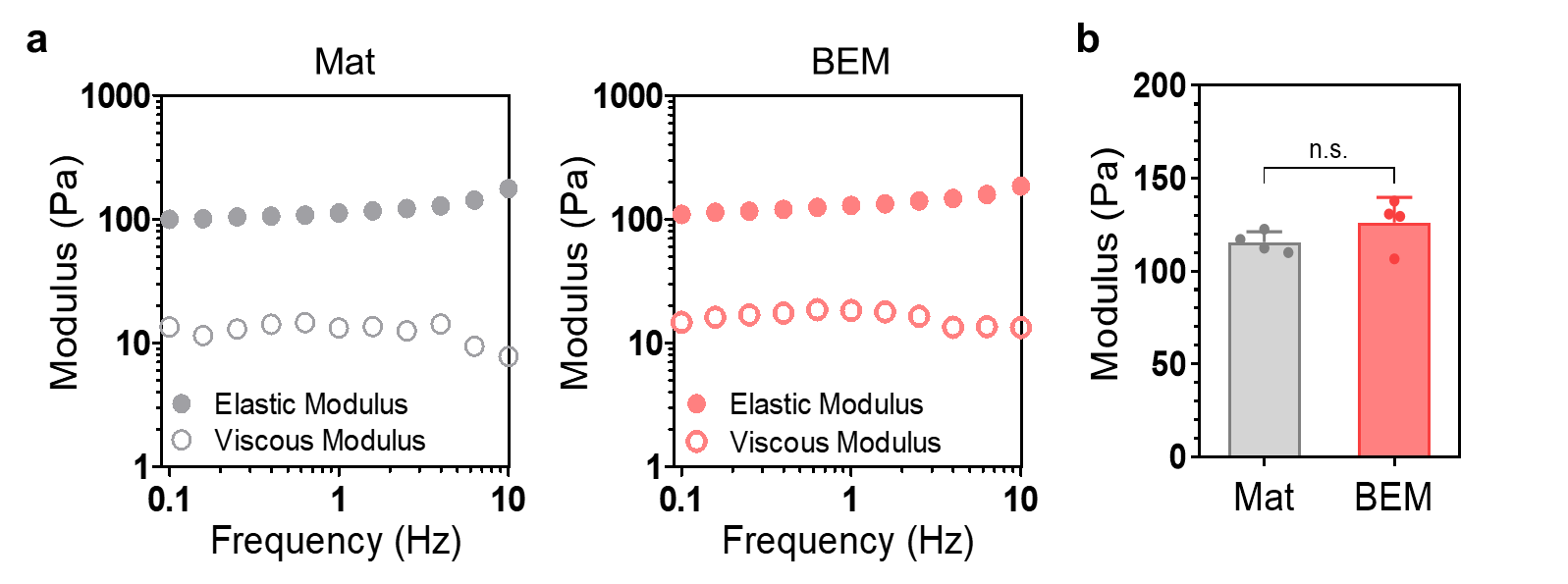
**

**Supplementary Figure 4. Mechanical properties of the decellularized BEM hydrogel.** (**a**) Rheometric analysis of 3D Matrigel (Mat) and BEM hydrogels in a frequency sweep mode. Filled circles are elastic moduli, and empty circles are viscous moduli. (**b**) Average modulus of 3D Mat and BEM hydrogels at 1 Hz frequency (*n* = 4 per group, n.s. = not significant, unpaired two-tailed *t*-test). Independent replicates = 2. All data are expressed as mean ± SD. Source data are provided as a Source Data file.


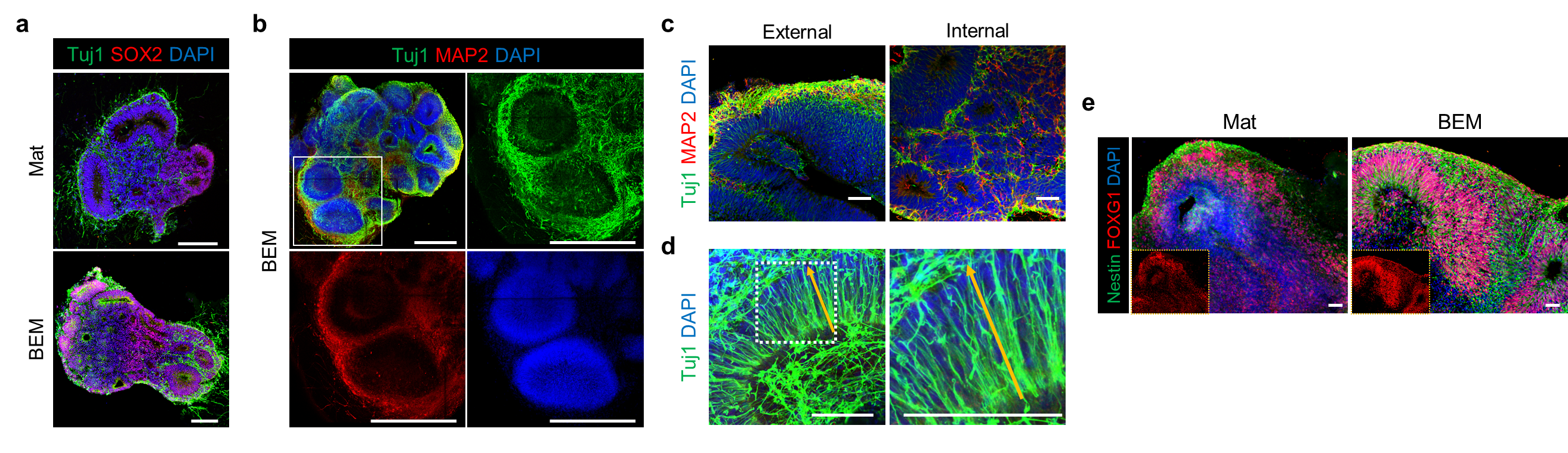


**Supplementary Figure 5. Radially-organized neuronal distribution in brain organoids cultured in the 3D BEM.** (**a**) Co-immunostaining of Tuj1 and SOX2, and (**b**) Tuj1 and MAP2 in BEM organoids at 30 days of culture (scale bars = 500 μm, independent replicates = 2). (**c**) Immunostaining of Tuj1 and MAP2 to visualize neuronal distribution at the external and internal side of BEM organoids at day 30 (scale bar = 100 μm, independent replicates = 2). (**d**) Immunostaining against Tuj1 in the BEM organoid at day 30, showing neuronal cell extension directed from the apical region to the basal side, forming an apico-to-basal axis (scale bars = 50 μm, independent replicate = 1). (**e**) Comparison of the expression of the neural progenitor marker (Nestin) and the forebrain marker (FOXG1) between Mat and BEM organoids at day 75 (scale bars = 50 μm, independent replicates = 3).

**
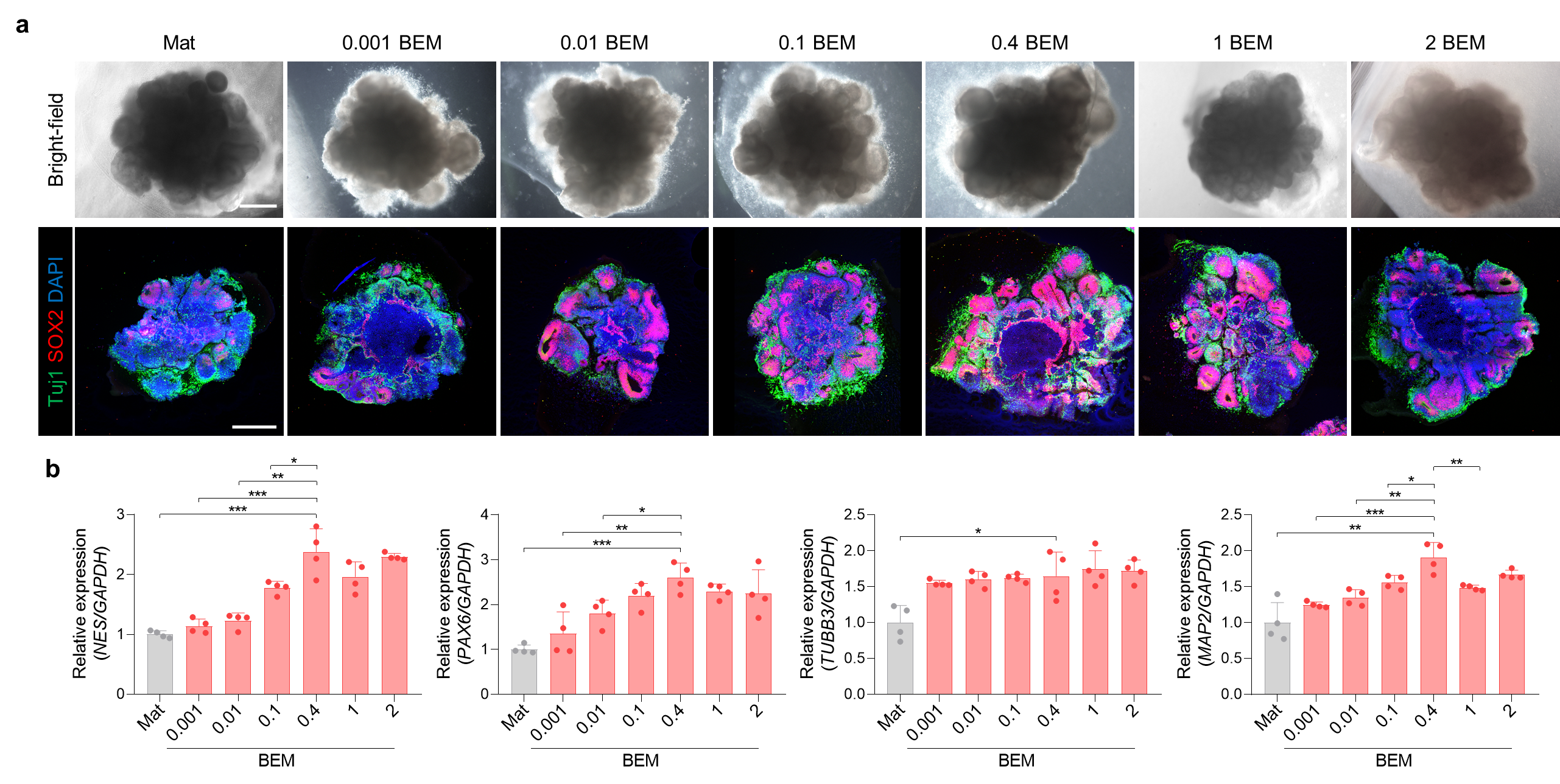
**

**Supplementary Figure 6. The effect of BEM concentrations on brain organoid generation at day 30.** (**a**) Microscopic images (scale bar = 500 μm, independent replicates = 2) and immunohistochemically stained images for SOX2 and Tuj1 in brain organoids cultured in various concentrations of BEM hydrogels at a range from 0.001 mg/ml to 2 mg/ml (scale bar = 500 μm, independent replicates = 2). (**b**) qPCR analysis to compare the expression of neural progenitor cell (*Nestin*), radial glial cell (*PAX6*), and neuronal cell (*TUBB3, MAP2*) markers between brain organoids encapsulated in various concentrations of BEM hydrogels (*n* = 4 per group, Mat versus 0.4 BEM *p* = 0.0004, 0.001 BEM versus 0.4 BEM *p* = 0.0009, 0.01 BEM versus 0.4 BEM *p* = 0.0013, 0.1 BEM versus 0.4 BEM *p* = 0.0244 for *NES*; Mat versus 0.4 BEM *p* < 0.0001, 0.001 BEM versus 0.4 BEM *p* = 0.0052, 0.01 BEM versus 0.4 BEM *p* = 0.0109 for *PAX6*; Mat versus 0.4 BEM *p* = 0.0199 for *TUBB3*; Mat versus 0.4 BEM *p* = 0.0019, 0.001 BEM versus 0.4 BEM *p* = 0.0007, 0.01 BEM versus 0.4 BEM *p* = 0.003, 0.1 BEM versus 0.4 BEM *p* = 0.0219, 1 BEM versus 0.4 BEM *p* = 0.0066 for *MAP2*, independent replicate = 1). All data are expressed as mean ± SD. Statistical differences between the groups were determined with unpaired two-tailed *t*-test (**p* < 0.05, ***p* < 0.01, ****p* < 0.001). Source data are provided as a Source Data file.

**
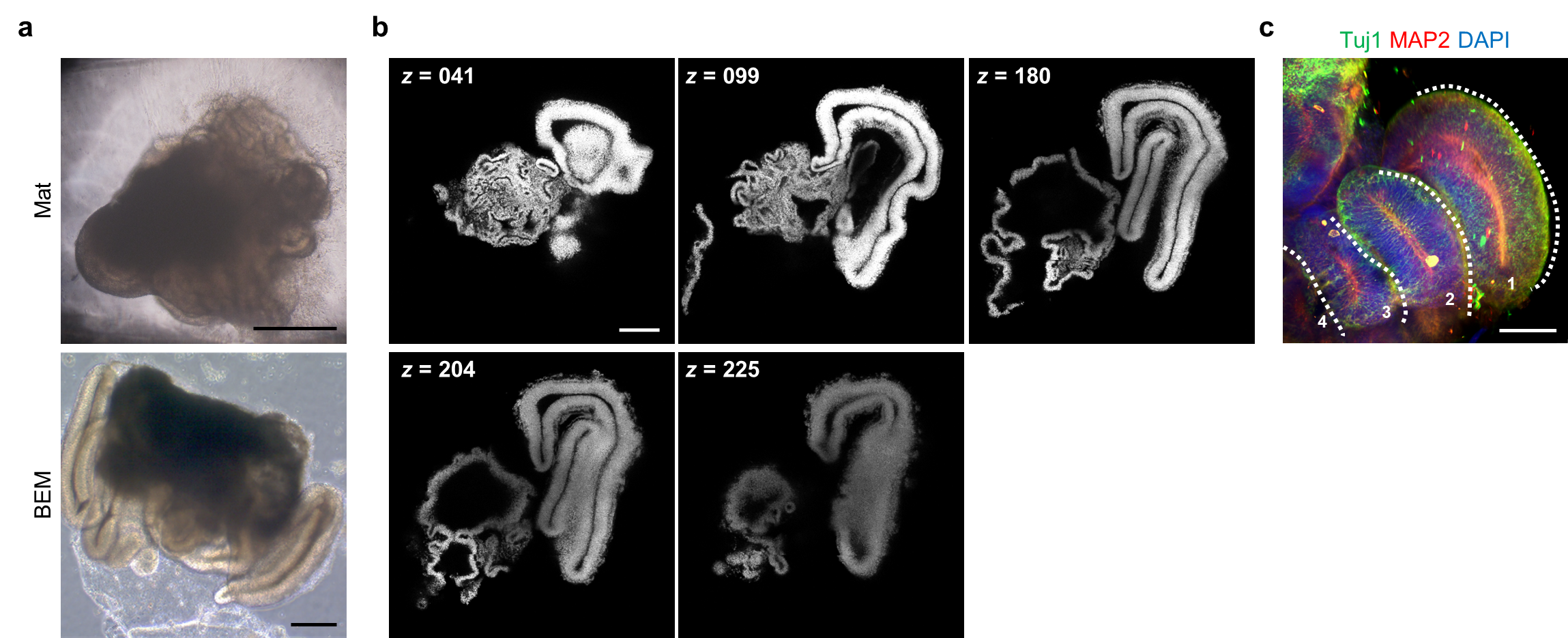
**

**Supplementary Figure 7. Cortical layer structures of brain organoids grown in BEM hydrogel.** (**a**) Bright-field images of Mat and BEM organoids at day 75 (scale bars = 500 μm, independent replicates = 5). (**b**) DAPI-stained BEM organoids processed with tissue clearing by the CUBIC method showing elongated cortical structures containing radially organized cell populations at day 30 (scale bar = 1 mm, independent replicate = 1). (**c**) Tuj1- and MAP2-immunostained BEM organoid at day 75 (scale bar = 200 μm, independent replicates = 3).

**Supplementary Figure 8. Enriched gene expression related to brain diseases in BEM organoids.** Gene ontology term enrichments for the risk genes related to central nervous system diseases in BEM organoids versus Mat organoids. Source data are provided as a Source Data file.

**
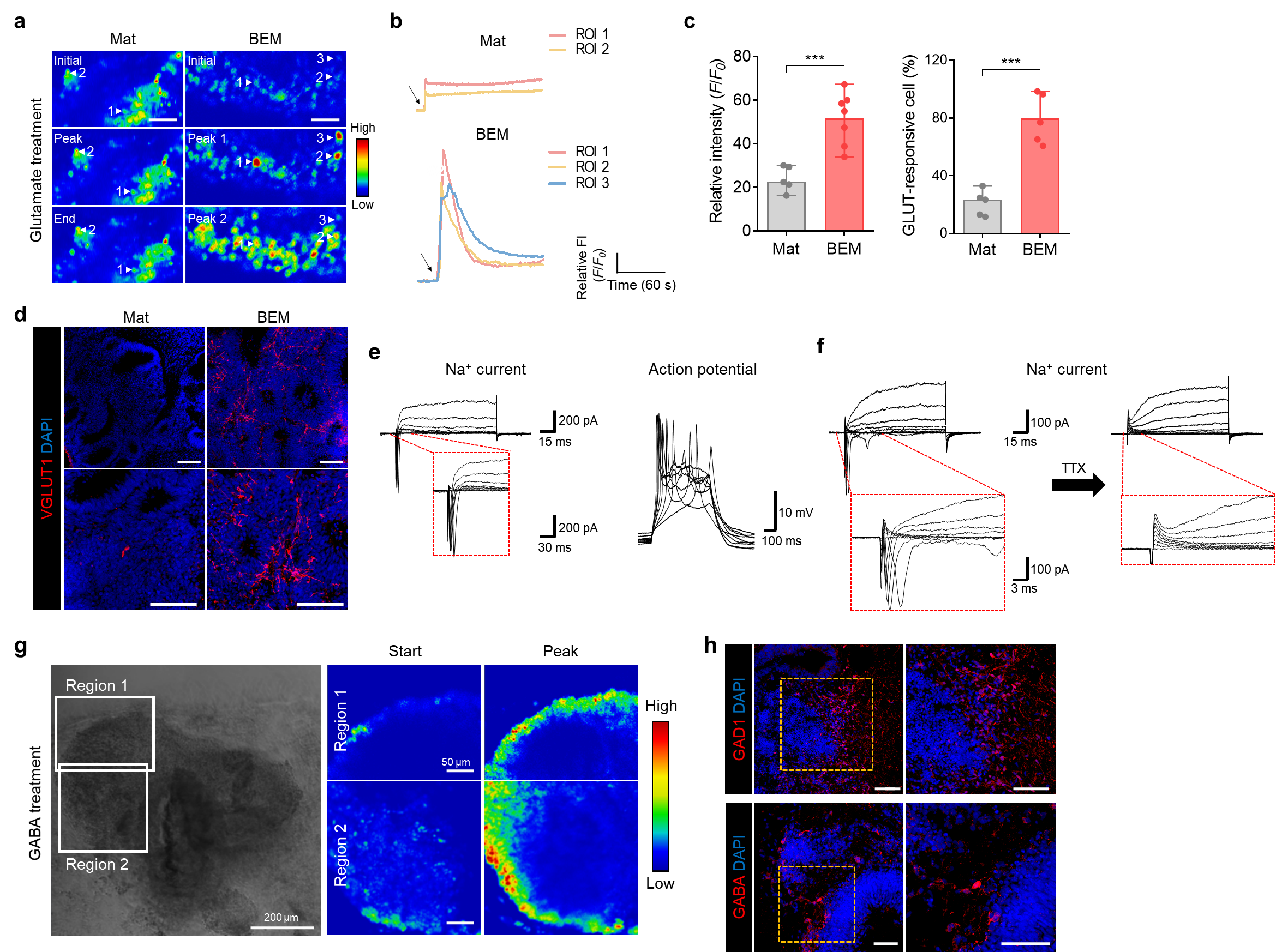
**

**Supplementary Figure 9. Functional properties of cells in BEM-embedded organoids on day 45.** (**a**) Ratiometric images of Fluo-4 AM-loaded cells in 45-day-old organoids in Mat and BEM groups before and after the addition of 100 μM glutamate (scale bar = 50 μm, independent replicates =3). (**b**) Representative time-course changes in Fluo-4 AM fluorescence intensity after applying glutamate to organoids. (**c**) Calcium imaging-based quantification analyses of relative intensity (F/F_0_) at the maximum response and responsive cell population after glutamate treatment (*n* = 5 for Mat group and *n* = 7 for BEM group, Mat versus BEM *p* = 0.0008 for relative intensity (left); *n* = 5, Mat versus BEM *p* = 0.0002 for GLUT-responsive cell (right), independent replicates = 2). All data are expressed as mean ± SD. Statistical differences between the groups were determined with unpaired two-tailed *t*-test (****p* < 0.001). (**d**) Immunostaining of glutamatergic neuronal marker VGLUT1 in 45-day-old Mat and BEM organoids (scale bars = 100 μm, independent replicates = 2). (**e, f**) The electrophysiological analyses of cells in the BEM organoid on day 45 (independent replicates = 2). (**e**) Representative Na^+^ current traces recorded in a voltage-clamp mode (left). Full traces are in the upper panel, and higher magnification is in the lower panel. Traces of evoked action potentials in a current-clamp mode (right). (**f**) Traces of tetrodotoxin (TTX)-sensitive Na^+^ current in BEM organoid. (**g**) Calcium imaging of BEM organoid in response to 50 μM γ-aminobutyric acid (GABA). A bright-field image of the BEM organoid (left, scale bar = 200 μm) and radiometric images of two different regions as marked on the bright-field image before and after adding GABA (right, scale bars = 50 μm, independent replicate = 1). (**h**) Immunostaining of BEM organoids at day 45 for GABAergic neuron markers glutamic acid decarboxylase 1 (GAD1) and GABA (scale bars = 100 μm, independent replicates = 2). Source data are provided as a Source Data file.

**
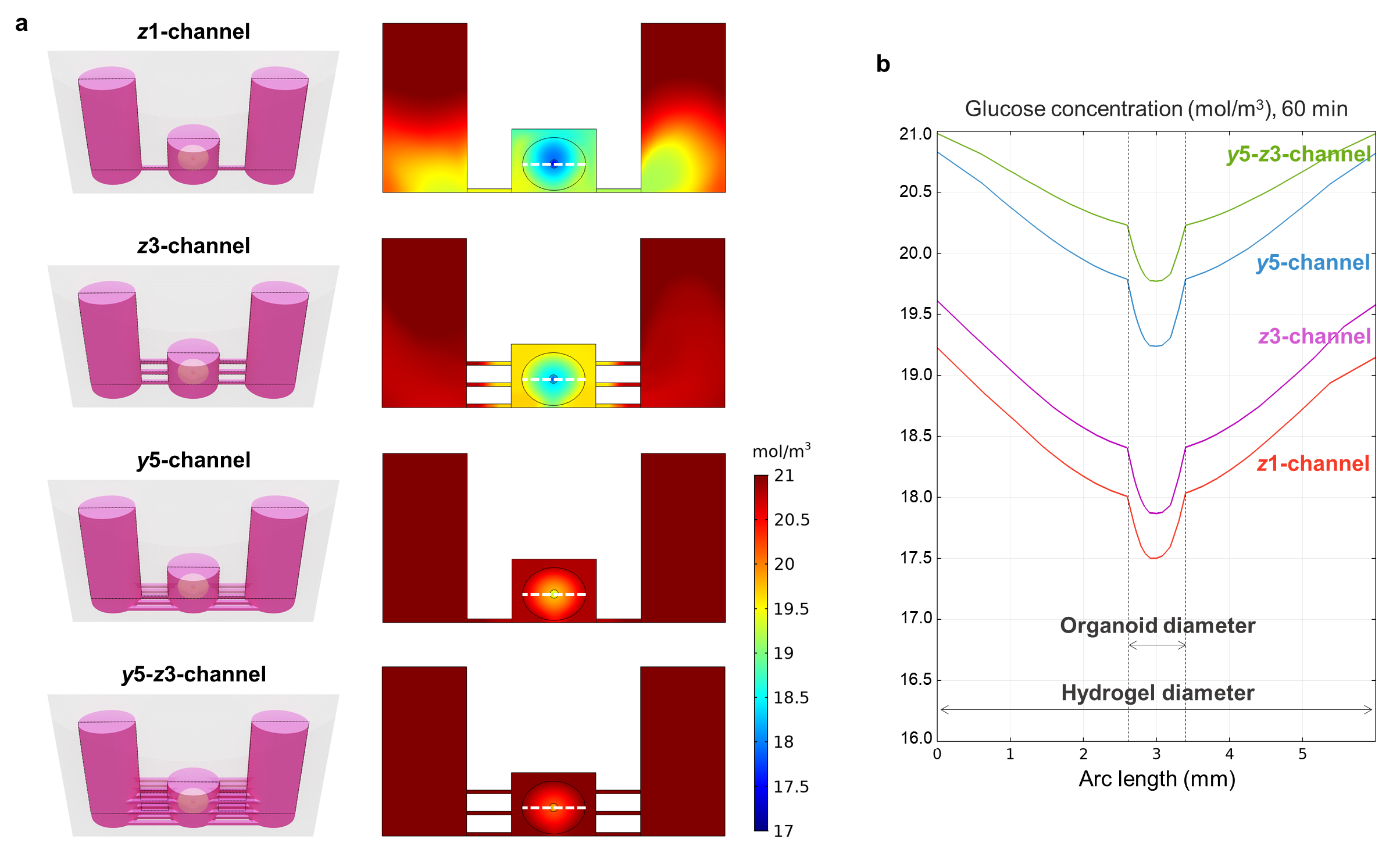
**

**Supplementary Figure 10. Simulation of glucose in a simplified version of microfluidic devices having different numbers of channels using COMSOL Multiphysics® software.** (**a**) Graphic visualization of the diffusive fluid flow state in the device after 60 min. The illustrations on the left panels indicate the frames of the cross-sectional images shown on the right panels. The color scale represents the overall ranges of glucose concentration. (**b**) Glucose distribution profiles along the white dashed lines in the cross-sectional images shown in (a). All factors used for the simulations are presented in Supplementary Table 5. Four different designs of the device were simulated; (1) *z*1-channel model in which an organoid chamber is connected to the medium reservoirs on both sides through one channel, (2) *z*3-channel model in which an organoid chamber is connected to the medium reservoirs on both sides through three channels vertically stacked along the *z*-axis, (3) *y*5-channel model in which an organoid chamber is connected to the medium reservoirs on both sides through five parallel channels along the *y*-axis at the fixed *z* plane, and (4) *y*5-*z*3-channel model comprising a combination of *z*3- and *y*5-channel designs.

**
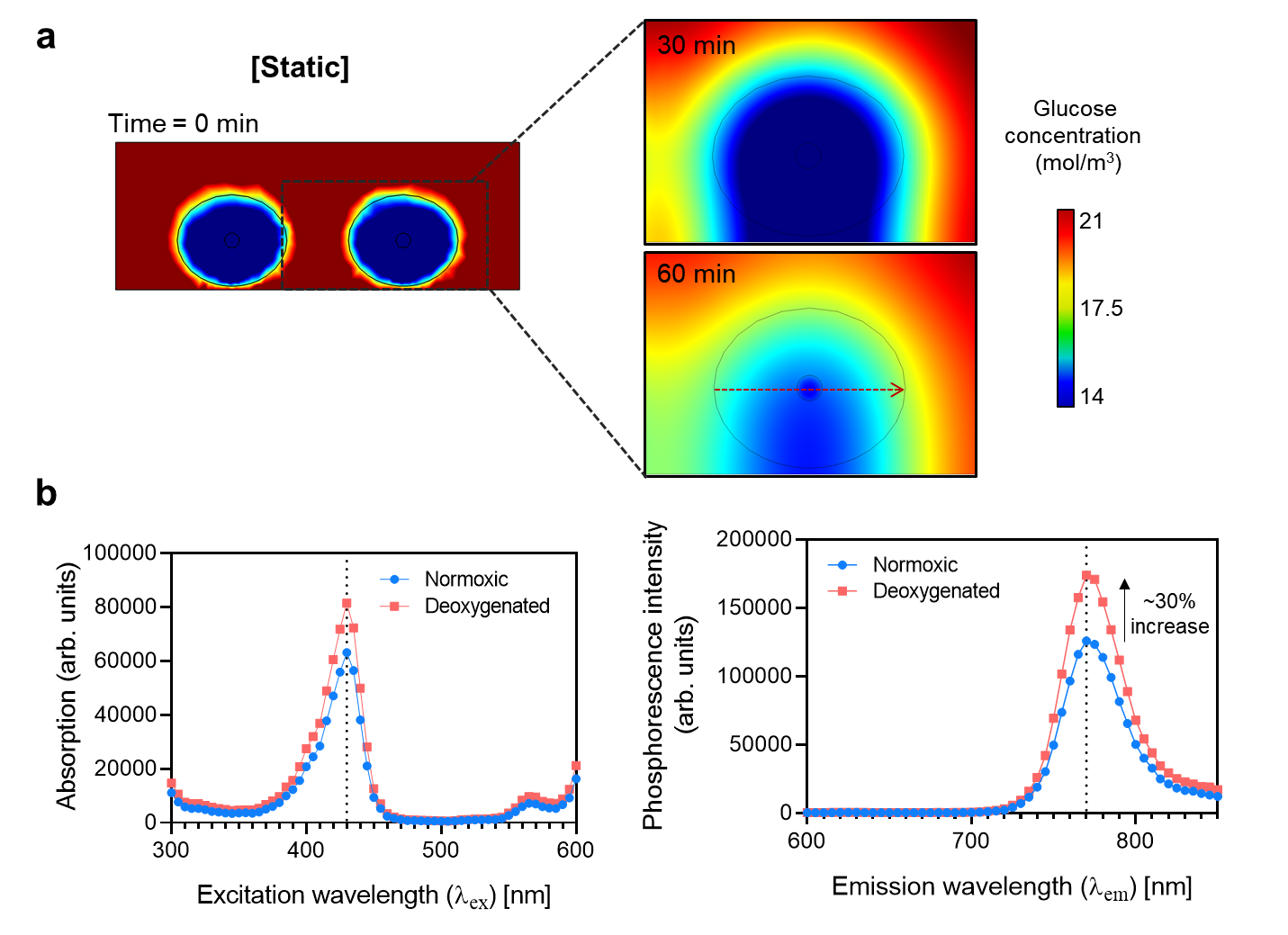
**

**Supplementary Figure 11. Simulation of glucose diffusion in the microfluidic device and measurement of oxygen levels by oxygen-sensitive phosphor nanoparticles.** (**a**) The computational simulation of glucose diffusion in the microfluidic device under static conditions. (**b**) The phosphorescence signal profiles of Pt(II) meso-tetra(pentafluorophenyl)porphine (PtTFPP)-poly(urethane acrylate nonionomer) (PUAN) nanoparticles in the normoxic and deoxygenated states at excitation wavelengths in the range of 300 to 600 nm and emission wavelengths in the range of 600 to 850 nm (independent replicate = 1). Spectral characterization under both normoxic and deoxygenated conditions indicates that optical absorption of the PtTFPP-PUAN nanoparticles and corresponding emission of phosphorescence was at a maximum of 430 nm and 770 nm, respectively. Lower oxygen levels lead to higher phosphorescence intensity, which is attributed to collisional quenching between oxygen molecules and PtTFPP in PUAN nanoparticles. The emission spectra reveal that the phosphorescence intensity of PtTFPP-PUAN nanoparticles under the deoxygenated conditions was approximately 30% higher than that under normoxic conditions. Source data are provided as a Source Data file.

**
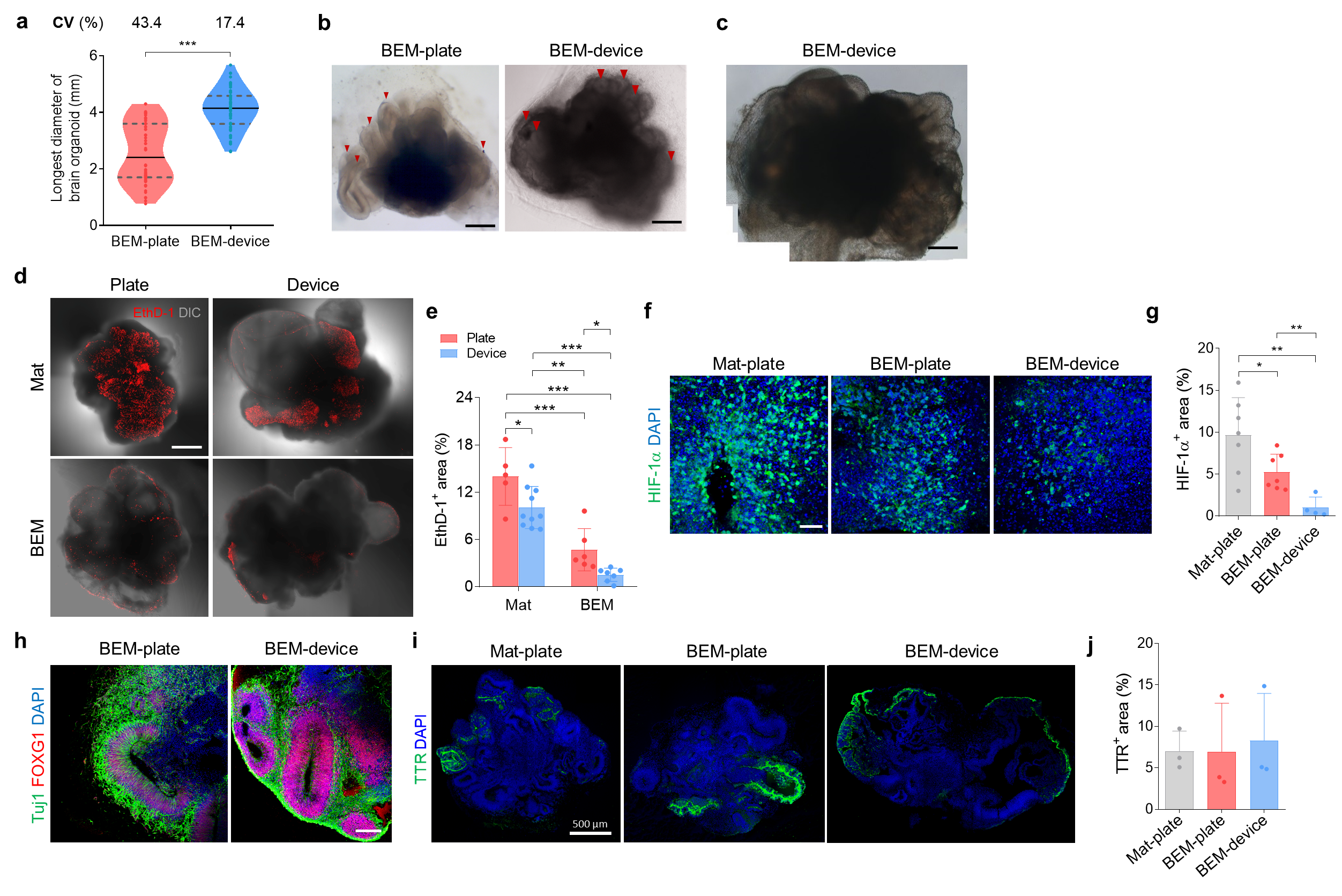
**

**Supplementary Figure 12. The investigation of the effects of microfluidic culture on cerebral organoids.** (**a**) Average and variation of the longest diameter of 60-day organoids grown in BEM hydrogel in the plate or device, which were quantified based on bright-field images. Data are expressed as violin plots. Dark grey dashed lines and black lines indicate 25~75% quartiles and median, respectively (*n* = 62 for BEM-plate group and *n* = 58 for BEM-device group, BEM-plate versus BEM-device *p* < 0.0001). The coefficient of variation (CV) of each group is indicated above the graph. (**b**) Representative bright-field images showing volumetric expansion and increased outgrowth of neuroepithelial structures in the BEM-device group compared to the BEM-plate group at day 30 (scale bars = 1 mm, independent replicates = 3). (**c**) The BEM-device organoid could grow up to 8 mm in diameter by day 60 (scale bar = 1 mm, independent replicates = 3). (**d**) Staining of ethidium homodimer-1 (EthD-1) to label dead cells in organoids cultured under different conditions at day 30 (scale bar = 500 μm), and (**e**) quantification of EthD-1^+^ area (*n* = 5, 6, 10, and 7 for Mat-plate, BEM-plate, Mat-device, and BEM-device groups, respectively; Mat-plate versus Mat-device *p* = 0.0328, Mat-plate versus BEM-plate *p* = 0.0009, Mat-plate versus BEM-device *p* < 0.0001, Mat-device versus BEM-plate *p* = 0.0017, Mat-device versus BEM-device *p* < 0.0001, BEM-plate versus BEM-device *p* = 0.0123, independent replicates = 3). (**f**) Immunostaining of hypoxia-inducible factor-1α (HIF-1α) in Mat-plate, BEM-plate, and BEM-device groups at day 120 (scale bar = 50 μm) and (**g**) quantification of HIF-1α^+^ area in each group (*n* = 7 for Mat-plate and BEM-plate groups and *n* = 4 for BEM-device group; Mat-plate versus BEM-plate *p* = 0.0383, Mat-plate versus BEM-device *p* = 0.0053, BEM-plate versus BEM-device *p* = 0.0062, independent replicates = 2). (**h**) Immunostaining of the forebrain marker FOXG1 and neuronal marker Tuj1 in the BEM-plate and BEM-device organoids at day 30 (scale bar = 200 μm, independent replicates = 4). (**i**) Immunostaining of choroid plexus marker Transthyretin (TTR) and (**j**) comparison of TTR^+^ area in 30-day cultured organoids (*n* = 3, independent replicates = 2). All data are expressed as mean ± SD. Statistical differences between the groups were determined by unpaired two-tailed *t*-test (**p* < 0.05, ***p* < 0.01, ****p* < 0.001). Source data are provided as a Source Data file.

**
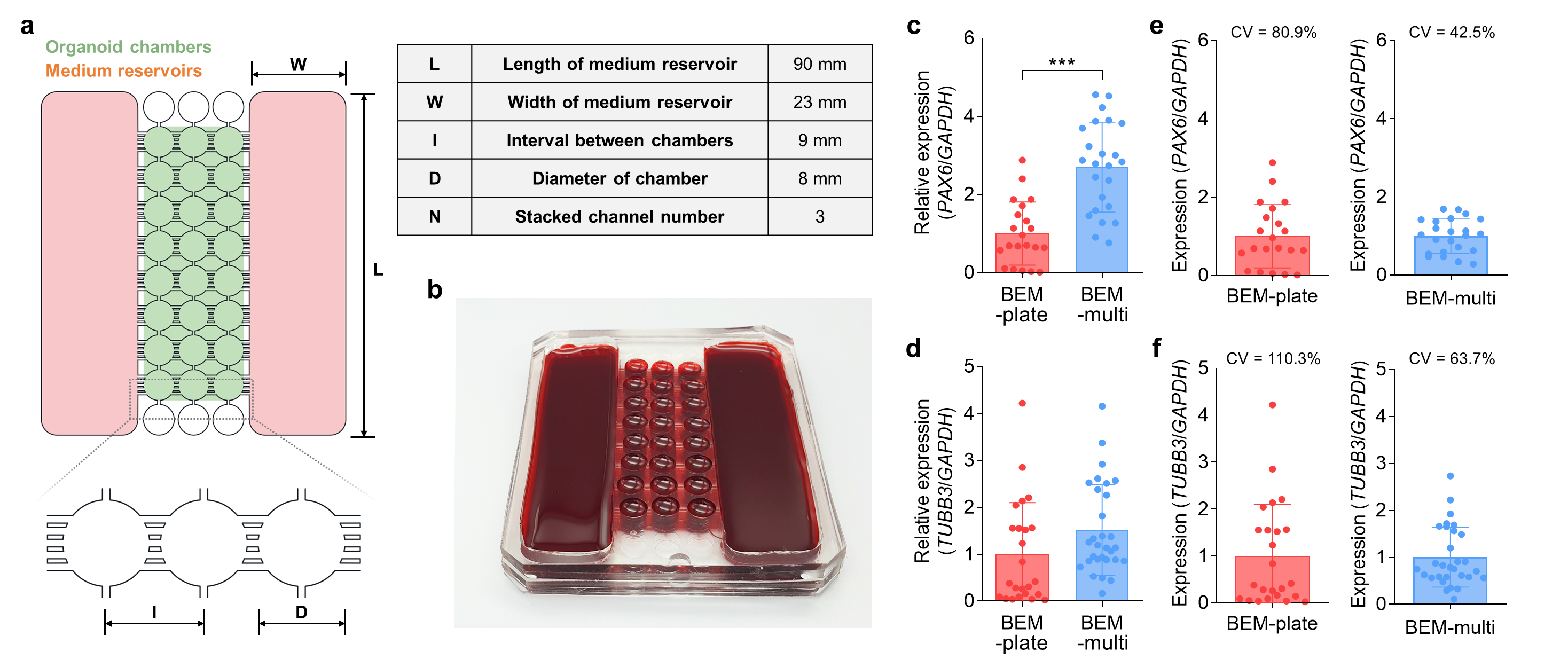
**

**Supplementary Figure 13. Evaluation of brain organoid variability.** (**a**) Schematics to illustrate the design of multi-well microfluidic device and table for dimensions of each part. (**b**) Picture of a multi-well microfluidic device. (**c-f**) Gene expression analysis of BEM-incorporated organoids cultured in well plates and multi-well device at 23 days of culture. Relative expression of (**c**) *PAX6* (*n* = 21 for BEM-plate group and *n* = 24 for BEM-multi group) and (**d**) *TUBB3* (*n* = 24 for BEM-plate group and *n* = 30 for BEM-multi group) in BEM organoids cultured in well plates and multi-well device (BEM-plate versus BEM-multi ****p* < 0.0001, independent replicate = 1). (**e**) *PAX6* and (**f**) *TUBB3* expression of BEM organoids normalized to the average for evaluating the sample variation in each group (CV; coefficient of variation) (*n* = 21 and 24 for *PAX6* in BEM-plate and BEM-multi groups, respectively, and *n* = 24 and 30 for *TUBB3* in BEM-plate and BEM-multi groups, respectively). All data are expressed as mean ± SD. Statistical differences between the groups were determined by unpaired two-tailed *t*-test. Source data are provided as a Source Data file.

**
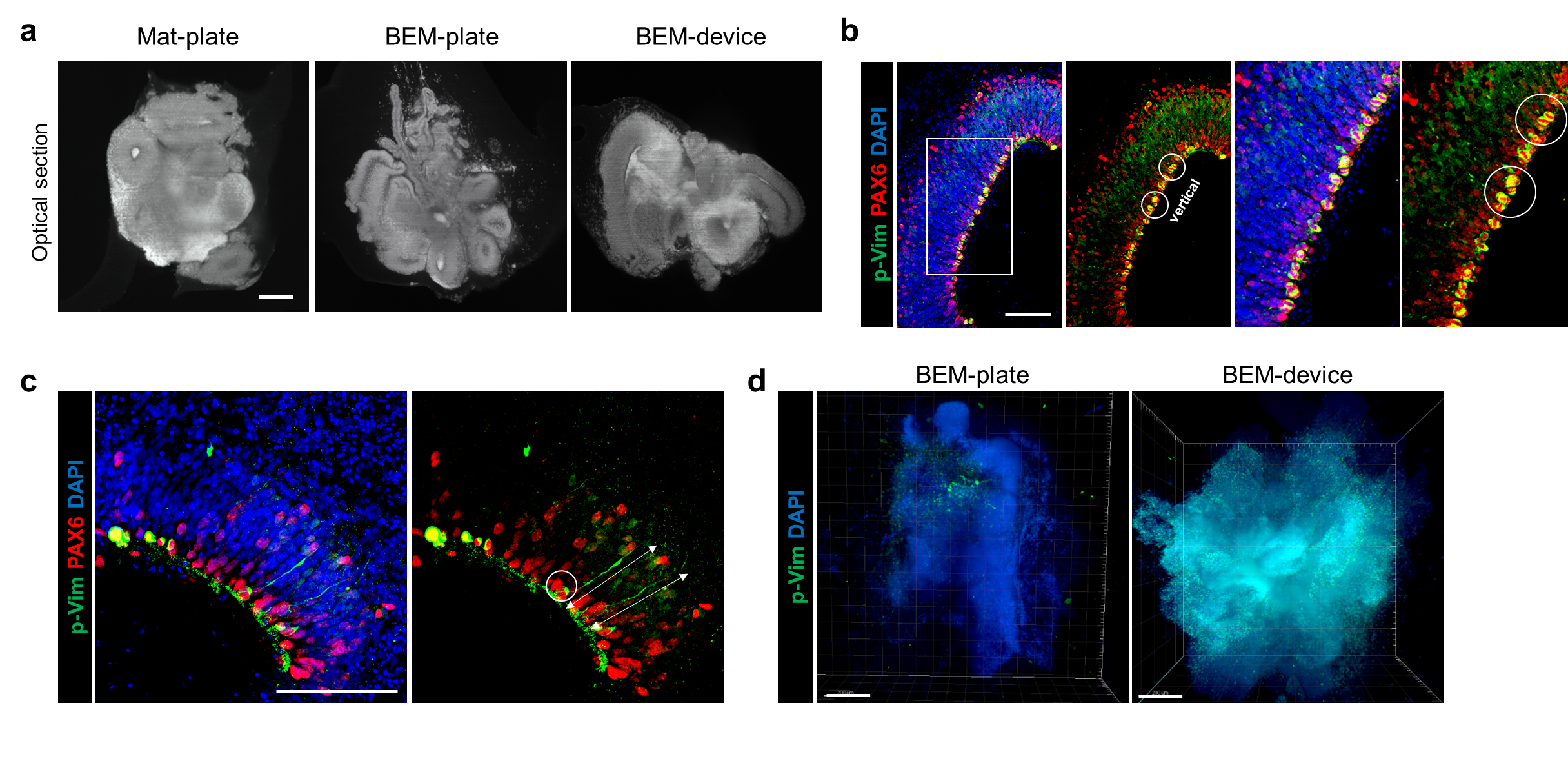
**

**Supplementary Figure 14.** **Complex structural morphology and radially organized radial glial cells (RGCs) in BEM-device organoids.** (**a**) Sectioned images of nuclei-stained (Syto16) organoids taken by light-sheet microscopy show complex inner and outer morphologies of the cortical regions in the BEM organoids compared with the Mat-plate organoids at day 30 (scale bar = 200 μm, independent replicates = 3). (**b, c**) Immunostaining of mitotic RGC marker p-vimentin (p-Vim) and RGC marker PAX6 in the BEM-device organoids at 30 days of culture (scale bars = 200 μm, independent replicates = 5). Highly proliferating RGCs (circle) and extended branches forming the apicobasal axis (arrows) were observed in the BEM-device organoid. (**d**) The 3D fluorescence imaging of the BEM-plate and BEM-device organoids immunostained for p-Vim at 30 days of culture (scale bars = 100 μm, independent replicate = 1).

**
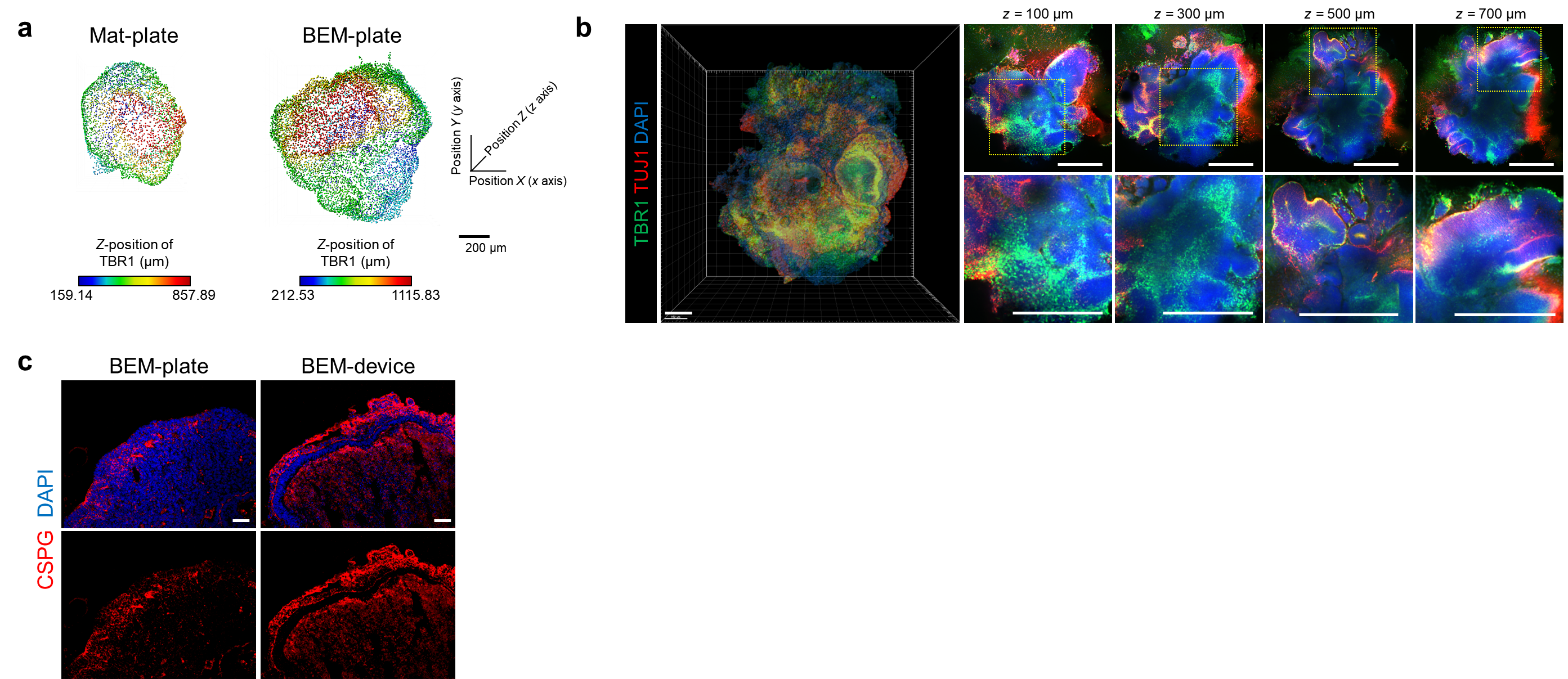
**

**Supplementary Figure 15. Deep-layer formation and preplate splitting in BEM organoids.** (**a**) 3D reconstructed images of the TBR1-stained Mat-plate and BEM-plate organoids at day 30, presenting different z-positions in the radiometric color spectrum (scale bar = 200 μm, independent replicates = 3). (**b**) 3D imaging of the BEM-device organoids immunostained against TBR1 and Tuj1 at day 30 (scale bars = 200 μm, independent replicate = 1). (**c**) Immunostaining against chondroitin sulfate proteoglycan (CSPG) indicating preplate splitting in brain organoids at day 60 (scale bar = 100 μm, independent replicate = 1).

**
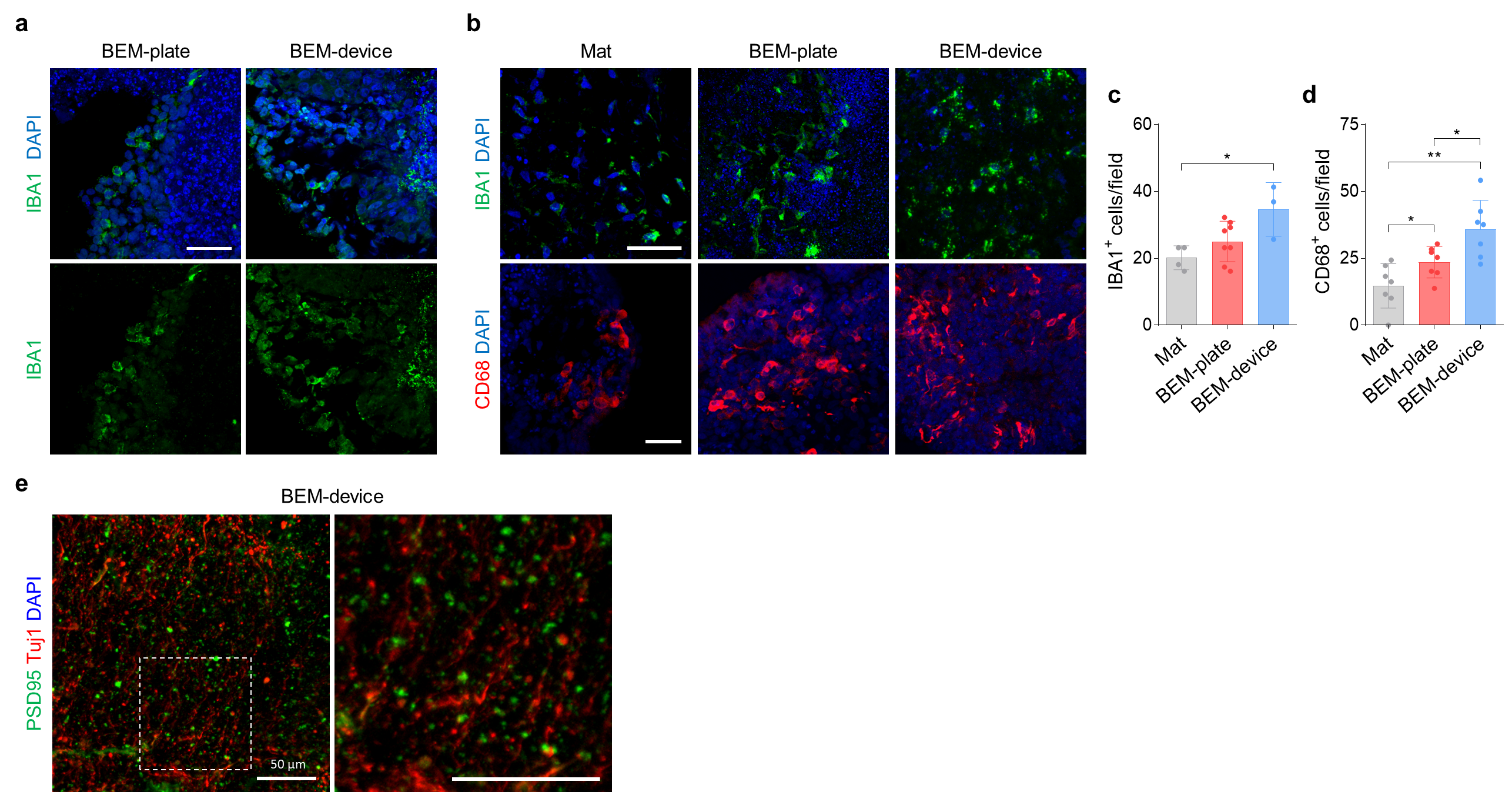
**

**Supplementary Figure 16. Microglia and mature neurons in BEM-device organoids.** (**a**) Immunostaining analysis showing IBA1^+^ microglia in the brain organoids at day 30 (scale bar = 50 μm, independent replicates = 2). (**b**) Immunostaining for IBA1 and CD68 (scale bars = 100 μm, independent replicates = 3–5), and quantification of (**c**) IBA1^+^ (*n* = 4, 8, 3 for Mat, BEM-plate, and BEM-device groups, respectively; Mat versus BEM-device *p* = 0.0222, independent replicates = 3) and (**d**) CD68^+^ (*n* = 7, Mat versus BEM-plate *p* = 0.0398, Mat versus BEM-device *p* = 0.0014, BEM-plate versus BEM-device *p* = 0.0206, independent replicates = 5) microglia in Mat, BEM, and BEM-device organoids at day 74. All data are expressed as mean ± SD. Unpaired two-tailed *t*-test was conducted to determine statistical significance between groups (**p* < 0.05, ***p* < 0.01). (**e**) Immunostaining for postsynaptic marker PSD95 in BEM-device organoids at day 60 (scale bar = 50 μm, independent replicates = 2). Source data are provided as a Source Data file.

**
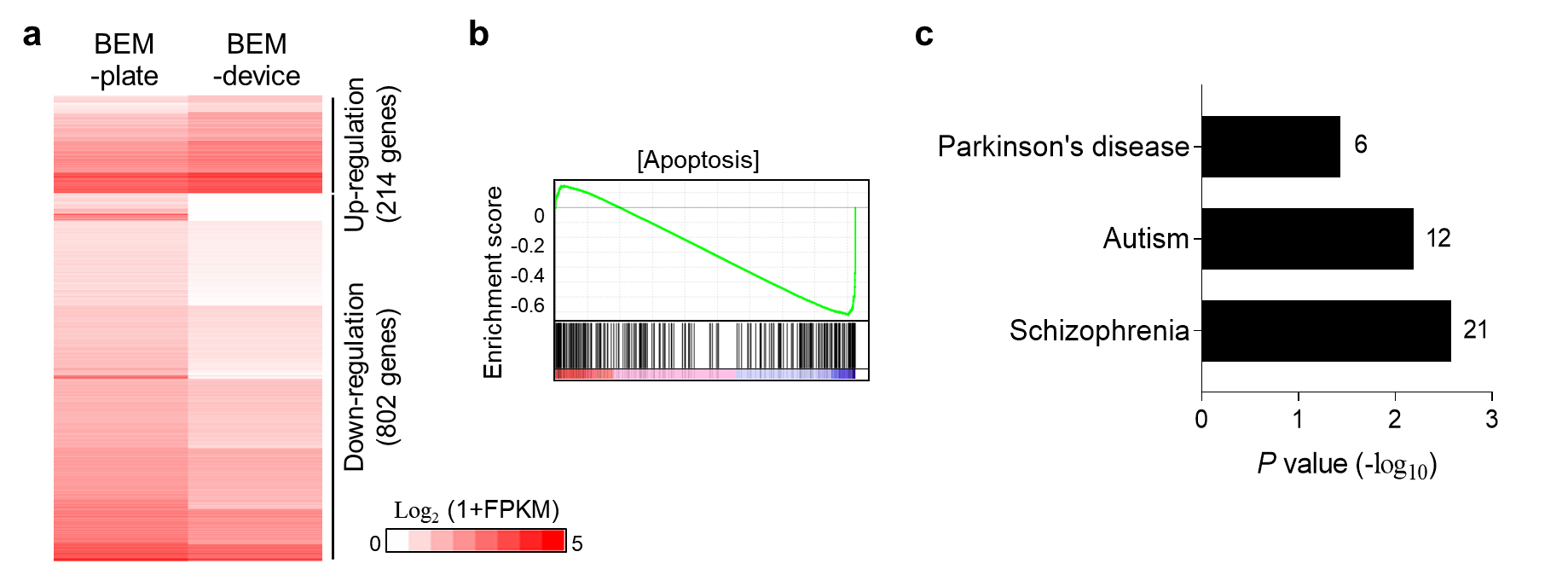
**

**Supplementary Figure 17. Comparison of global transcriptomes between BEM-plate and BEM-device organoids at day 75.** (**a**) Heatmap of differentially expressed genes from RNA-sequencing analysis performed on BEM organoids cultured in a plate and device (*n* = 3 per group, independent replicate = 1). (**b**) The gene set enrichment analysis (GSEA) of apoptosis probe set in the BEM-device organoids versus BEM-plate organoids. (**c**) Gene ontology term enrichments for the genes associated with central nervous system diseases in BEM-device organoids versus BEM-plate organoids. Source data are provided as a Source Data file.

**
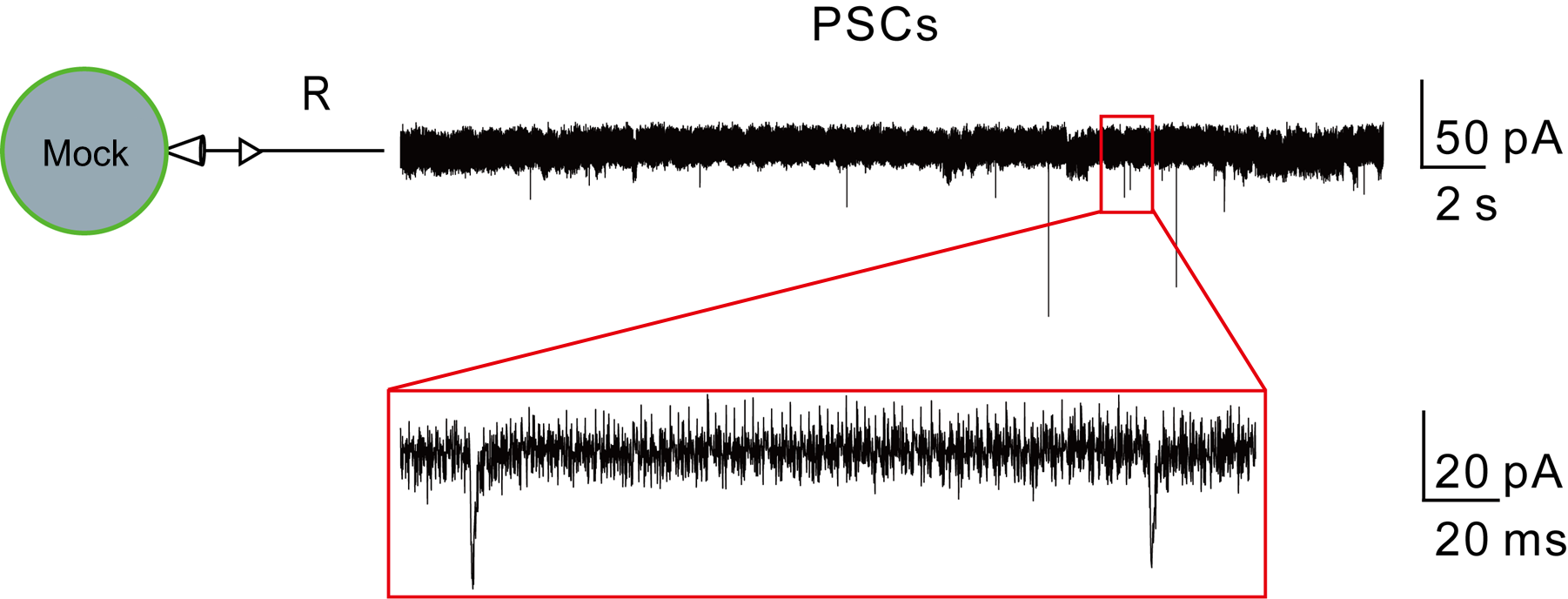
**

**Supplementary Figure 18. Spontaneous postsynaptic currents (PSCs) of a neuron within 60-day-old organoids cultured in the BEM device.** Independent replicate = 1.

**
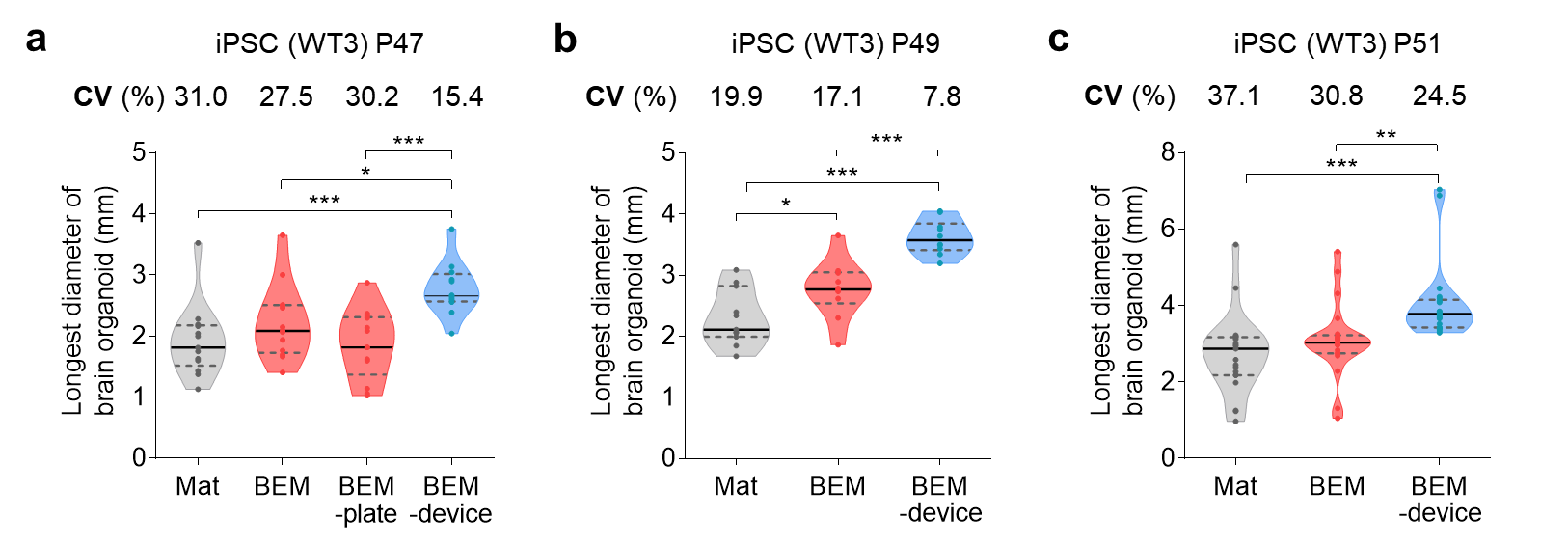
**

**Supplementary Figure 19. Comparison of variation in the size of cerebral organoids at 60 days of culture.** (**a–c**) Measurements of the longest diameter of brain organoids from iPSCs at different passage numbers. Data are expressed as violin plots. Dark grey dashed lines and black lines indicate 25~75% quartiles and median, respectively. The graphs are plotted separately by the biological samples with the same iPSC passage number. Coefficient of variation (CV) values are indicated above the graphs (*n* = 13 for Mat, BEM, and BEM-plate groups, and *n* = 12 for BEM-device group for (**a**), *n* = 11 for Mat group and *n* = 10 for BEM and BEM-device groups for (**b**), and *n* = 21 for (**c**), independent replicates = 3). All data are expressed as mean ± SD. Unpaired two-tailed *t*-test was conducted to determine statistical significance between groups (Mat versus BEM-device *p* = 0.0004, BEM versus BEM-device *p* = 0.0164, BEM-plate versus BEM-device *p* = 0.0001 for (**a**); Mat versus BEM *p* = 0.0315, Mat versus BEM-device *p* < 0.0001, BEM versus BEM-device *p* = 0.0001 for (**b**); Mat versus BEM-device *p* = 0.0002, BEM versus BEM-device *p* = 0.0033 for (**c**); **p* < 0.05, ***p* < 0.01 ****p* < 0.001). Source data are provided as a Source Data file.

**
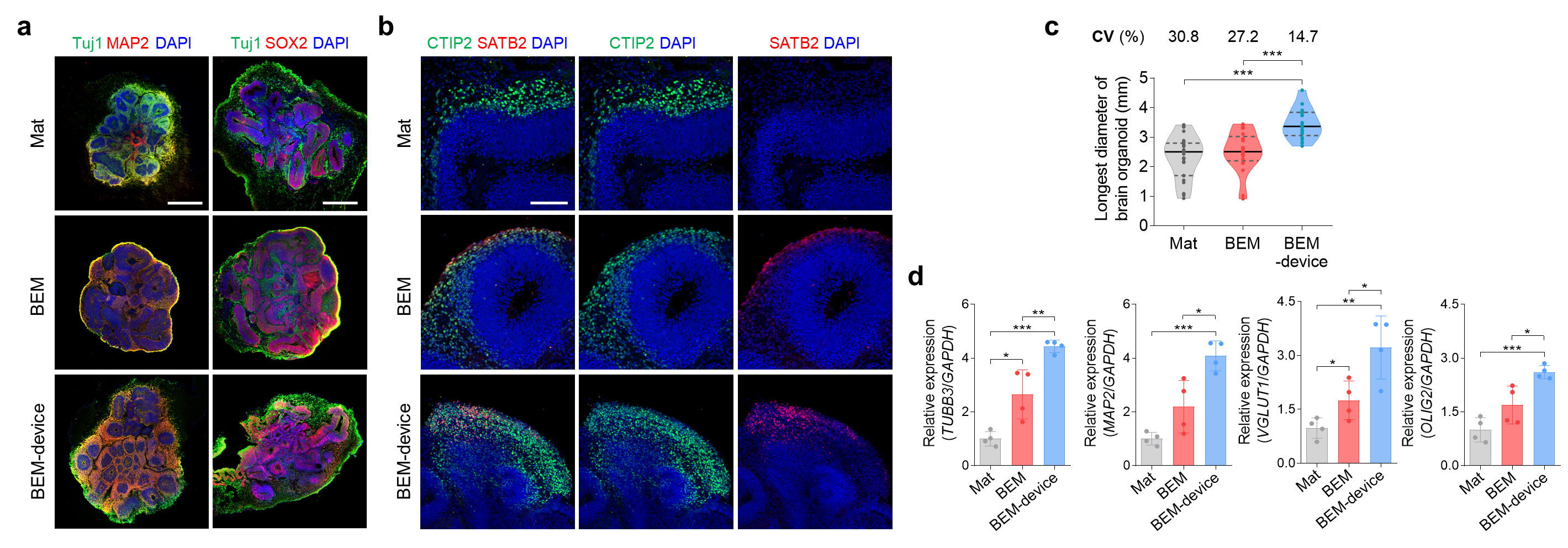
**

**Supplementary Figure 20. Confirmation of the effects of BEM and the microfluidic device on development of cerebral organoids generated from a second iPSC line (KYOU-DXR0109B).** (**a**) Immunostaining of Tuj1, MAP2, and SOX2 at day 30 (scale bars = 500 μm, independent replicates = 2), and (**b**) CTIP2 and SATB2 at day 60 (scale bars = 100 μm, independent replicates = 2). (**c**) Quantification of the longest diameter of the organoid at day 60. Data are expressed as violin plots. Dark grey dashed lines and black lines indicate 25~75% quartiles and median, respectively (*n* = 29, Mat versus BEM *p* < 0.0001, BEM versus BEM-device *p* < 0.0001). (**d**) Comparison of neuronal gene expression analyzed by qPCR in Mat, BEM, and BEM-device organoids at day 60 (*n* = 4, Mat versus BEM *p* = 0.0134, Mat versus BEM-device *p* < 0.0001, BEM versus BEM-device *p* = 0.0091 for *TUBB3*; Mat versus BEM-device *p* < 0.0001, BEM versus BEM-device *p* = 0.0149 for *MAP2*; Mat versus BEM *p* < 0.0441, Mat versus BEM-device *p* = 0.0028, BEM versus BEM-device *p* = 0.0286 for *VGLUT1*; Mat versus BEM-device *p* = 0.0002, BEM versus BEM-device *p* = 0.0168 for *OLIG2*, independent replicate = 1). All data are presented as mean ± SD. Statistical differences between the groups were determined with unpaired two-tailed *t*-test (**p* < 0.05, ***p* < 0.01, ****p* < 0.001). Source data are provided as a Source Data file.

**
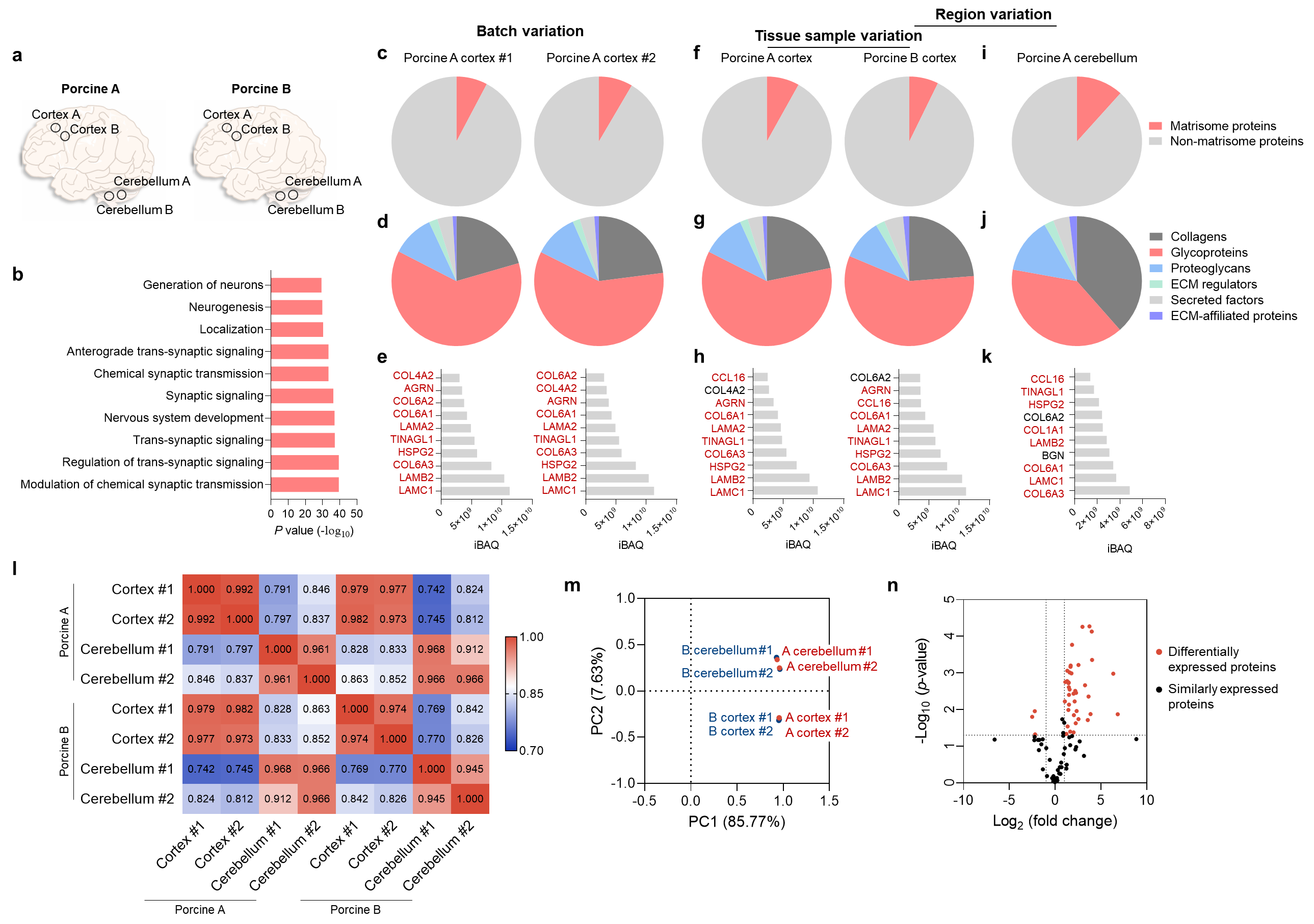
**

**Supplementary Figure 21. Proteomic analysis of BEM derived from porcine brain tissue.** (**a**) Cortex and cerebellum tissues isolated from two different porcine brains were processed for decellularization. (**b**) The lists of biological process terms of the gene ontology enrichment analysis of the genes identified in porcine cortex-derived BEM (pBEM) that are known to be highly expressed in the brain tissue compared to other tissues in the body (*n* = 4). The top 10 terms are ranked by the lowest *p* values. (**c-e**) Batch variation of pBEM derived from the same donor. (**c**) The percentage of matrisome proteins out of total proteins, (**d**) the composition of sub-categorized matrisome proteins, and (**e**) the most abundant 10 matrisome proteins identified in pBEM derived from the same porcine cortex (*n* = 1). (**f-h**) Tissue sample variation of pBEM derived from different donor. (**f**) The percent of matrisome proteins out of total proteins, (**g**) the composition of sub-categorized matrisome proteins, and (**h**) the most abundant 10 matrisome proteins identified in pBEM derived from different porcine cortex (*n* = 1). (**i-k**) Variation of pBEM derived from different brain region. (**i**) The percent of matrisome proteins out of total proteins, (**j**) the composition of sub-categorized matrisome proteins, and (**k**) the most abundant 10 matrisome proteins identified in pBEM derived from cerebellum (*n* = 4). (**l**) A heat map of Spearman’s rank correlation coefficients between various pBEM samples. (**m**) Principal component analysis (PCA) of pBEM samples. (**n**) Volcano plot of the expression of matrisome proteins in pBEM derived from cortex to compared with that in pBEM derived from cerebellum (*n* = 4). Red dots indicate differentially expressed proteins by at least 2-folds with *p* < 0.05. The black dotted vertical lines represent a fold change of 2 and the black dotted horizontal line represents a *p* value of 0.05. Source data are provided as a Source Data file.

**
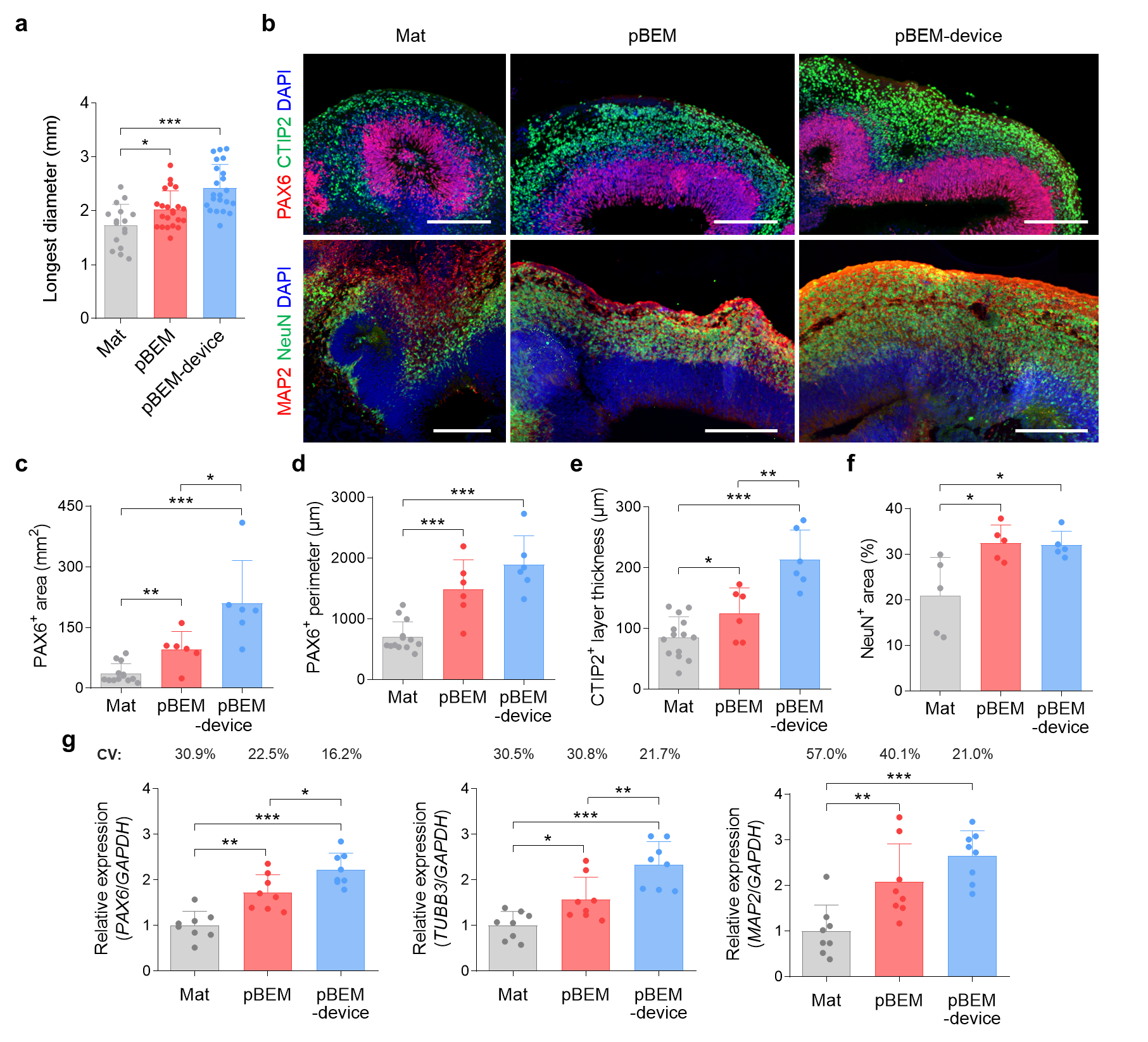
**

**Supplementary Figure 22. Application of pBEM and microfluidic system for human cerebral organoid culture.** (**a**) Measurements of the longest diameter of 60-day Mat, pBEM, and pBEM-device organoids in bright-field images (*n* = 16 for Mat group and *n* = 22 for pBEM and pBEM-device groups, Mat versus pBEM *p* = 0.0178, Mat versus pBEM-device *p* < 0.0001). (**b**) Immunohistochemically stained images for PAX6 and CTIP, and MAP2 and NeuN at day 60 (scale bars = 200 μm, independent replicates = 2). (**c**) Quantification of PAX6^+^ area (*n* = 13 for Mat group and *n* = 6 for pBEM and pBEM-device groups, Mat versus pBEM *p* = 0.0011, Mat versus pBEM-device *p* < 0.0001, pBEM versus pBEM-device *p* = 0.0353), (**d**) PAX6^+^ perimeter (*n* = 13 for Mat group and *n* = 6 for pBEM and pBEM-device groups, Mat versus pBEM *p* = 0.0002, Mat versus pBEM-device *p* < 0.0001), (**e**) CTIP2^+^ layer thickness (*n* = 14 for Mat group and *n* = 6 for pBEM and pBEM-device groups, Mat versus pBEM *p* = 0.0406, Mat versus pBEM-device *p* < 0.0001, pBEM versus pBEM-device *p* = 0.0066), and (**f**) NeuN^+^ area relative to DAPI^+^ area (*n* = 5, Mat versus pBEM *p* = 0.0234, Mat versus pBEM-device *p* = 0.0234) based on immunostained images of the organoids cultured for 60 days in different conditions. (**g**) qPCR analysis to compare the gene expression (*PAX6*, *TUBB3*, and *MAP2*) at day 60 (*n* = 8, Mat versus pBEM *p* = 0.001, Mat versus pBEM-device *p* < 0.0001, pBEM versus pBEM-device *p* = 0.0187 for *PAX6*; Mat versus pBEM *p* = 0.0135, Mat versus pBEM-device *p* < 0.0001, pBEM versus pBEM-device *p* = 0.0084 for *TUBB3*; Mat versus pBEM *p* = 0.0092, Mat versus pBEM-device *p* < 0.0001 for *MAP2*, independent replicate = 1). All data are expressed as mean ± SD. Unpaired two-tailed *t*-test was conducted to determine statistical significance between groups (**p* < 0.05, ***p* < 0.01, ****p* < 0.001). Source data are provided as a Source Data file.

**
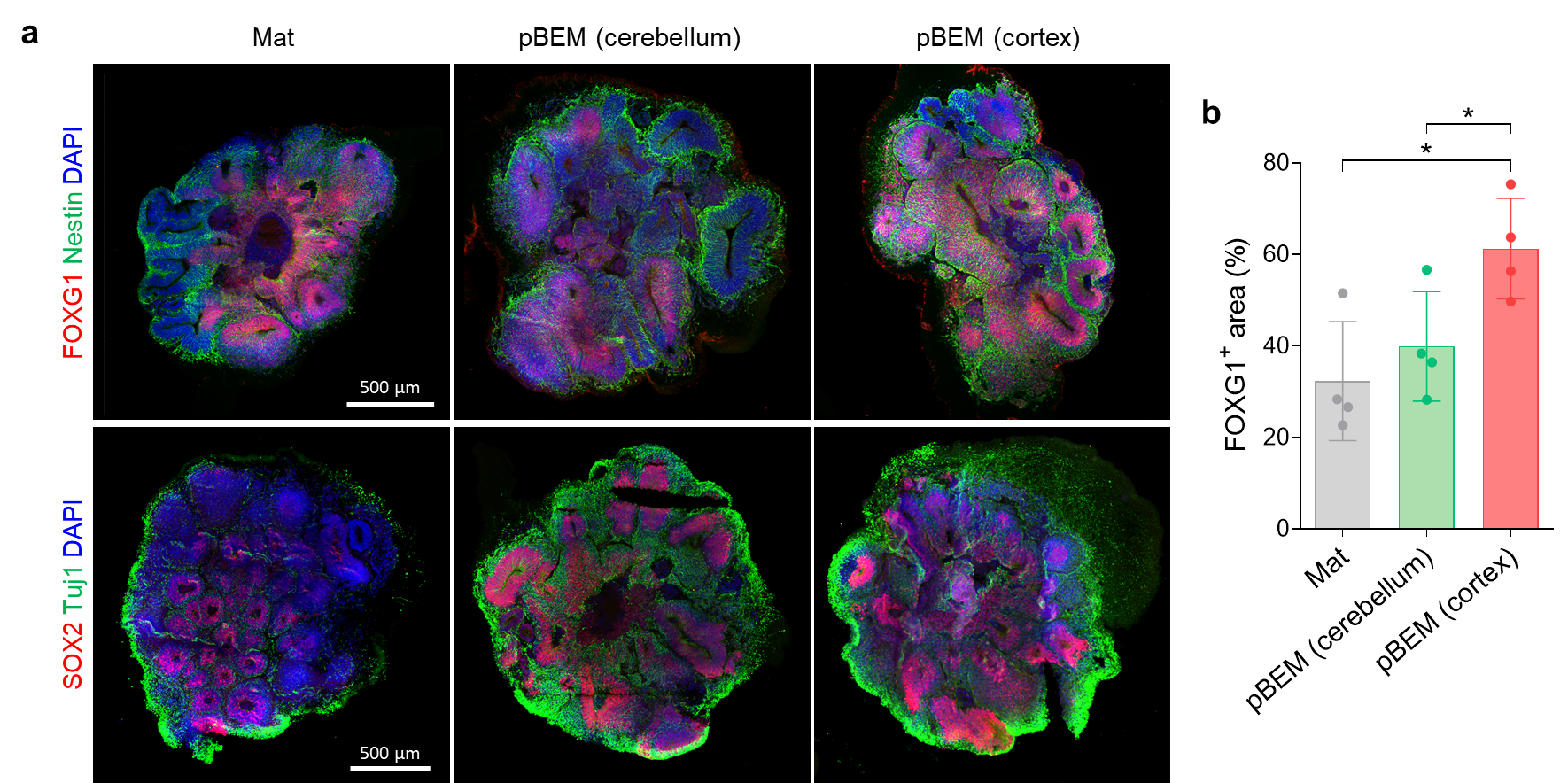
**

**Supplementary Figure 23. Comparison of brain organoids cultured in pBEM derived from cortex or cerebellum region.** (**a**) Immunostaining analysis for FOXG1 and Nestin, and SOX2 and Tuj1 in the organoids cultured for 30 days in Mat, pBEM derived from cerebellum, and pBEM derived from cortex (scale bars = 500 μm). (**b**) Quantification of FOXG1^+^ area relative to DAPI^+^ area (*n* = 4, independent replicate = 1). All data are expressed as mean ± SD. Unpaired two-tailed *t*-test was conducted to determine statistical significance between groups (**p* < 0.05; Mat versus pBEM (cortex) *p* = 0.0145, pBEM (cerebellum) versus pBEM (cortex) *p* = 0.039). Source data are provided as a Source Data file.

**
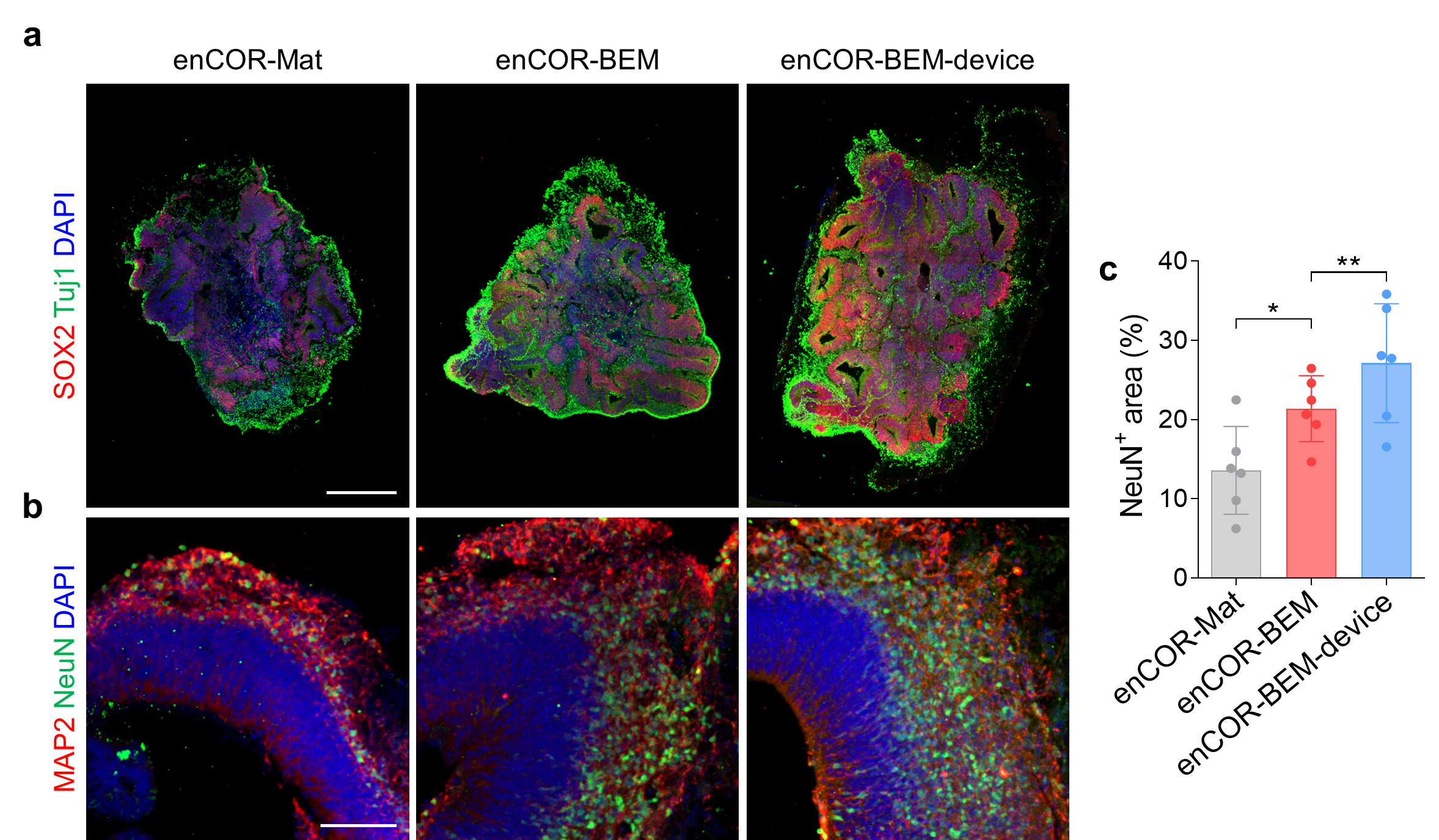
**

**Supplementary Figure 24. Application of BEM and microfluidic device for culturing microfilament-engineered cerebral organoids (enCORs).** Immunostained images for (**a**) SOX2 and Tuj1 (scale bar = 500 μm, independent replicate = 1), and (**b**) MAP2 and NeuN (scale bar = 100 μm, independent replicate = 1) of brain organoids encapsulated with Mat, BEM, or BEM in the microfluidic device at 30 days of culture. (**c**) Quantification of NeuN^+^ area relative to DAPI^+^ area (*n* = 6, independent replicate = 1). All data are expressed as mean ± SD. Unpaired two-tailed *t*-test was conducted to determine statistical significance between groups (**p* < 0.05, ***p* < 0.01; enCOR-Mat versus enCOR-BEM *p* = 0.0206, enCOR-BEM versus enCOR-BEM-device *p* = 0.0052). Source data are provided as a Source Data file.

**Supplementary Tables**

**Supplementary Table 1.** Information on the human brain tissue sources that were used to generate BEM.

**
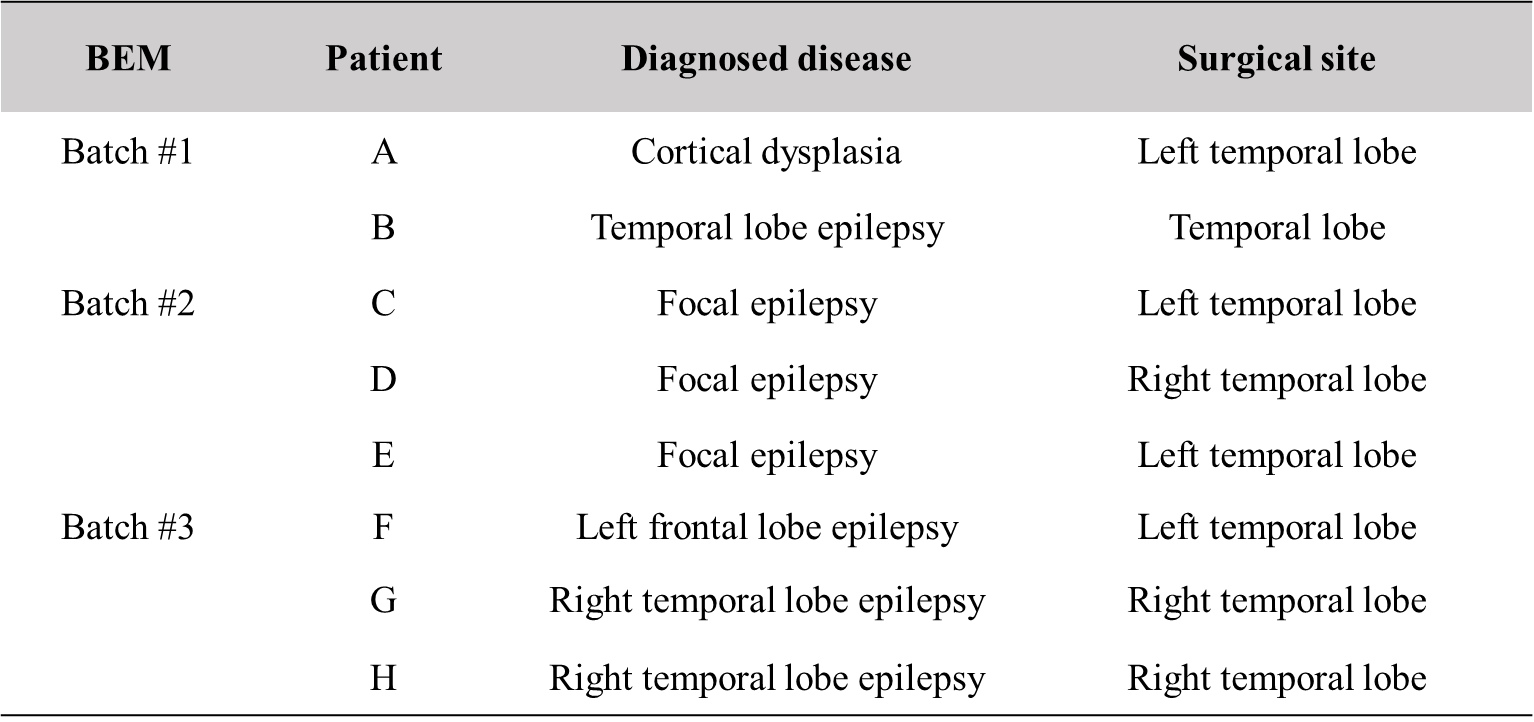
**

**Supplementary Table 2.** Lists of matrisome subcategorized as core matrisome proteins (subdivided as collagens, glycoproteins, and proteoglycans) and matrisome-associated proteins (subdivided as ECM-affiliated proteins, ECM regulators, and secreted factors) in Mat, human BEM (BEM), and porcine BEM (pBEM). Source data are provided as a Source Data file.

**
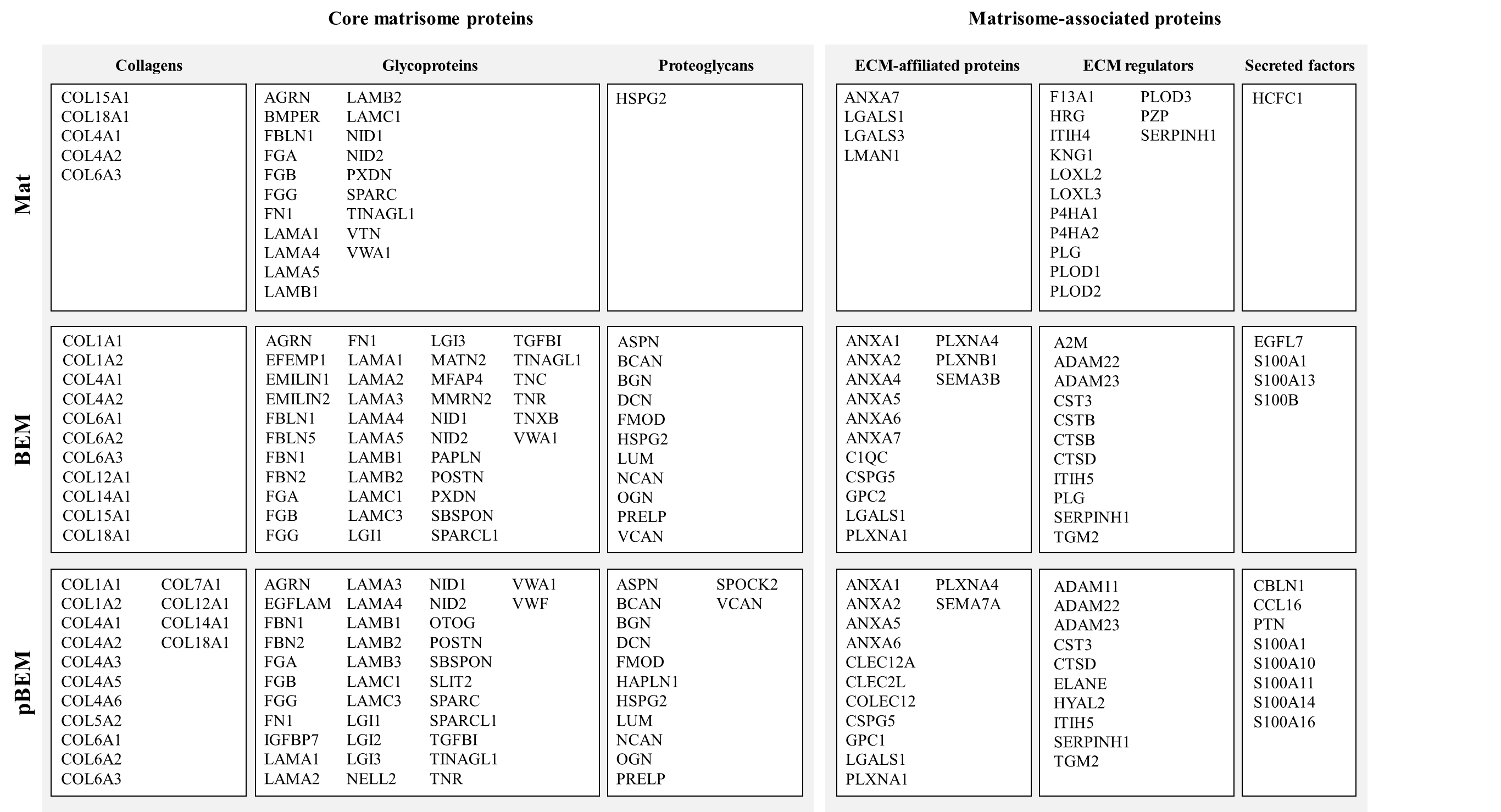
**

**Supplementary Table 3.** Sub-categorized matrisome proteins identified in Mat, human BEM (BEM), and porcine BEM (pBEM) by proteomic analysis in this study, and human brain tissue from the previously reported study.[^1^](#_ENREF_1)

**
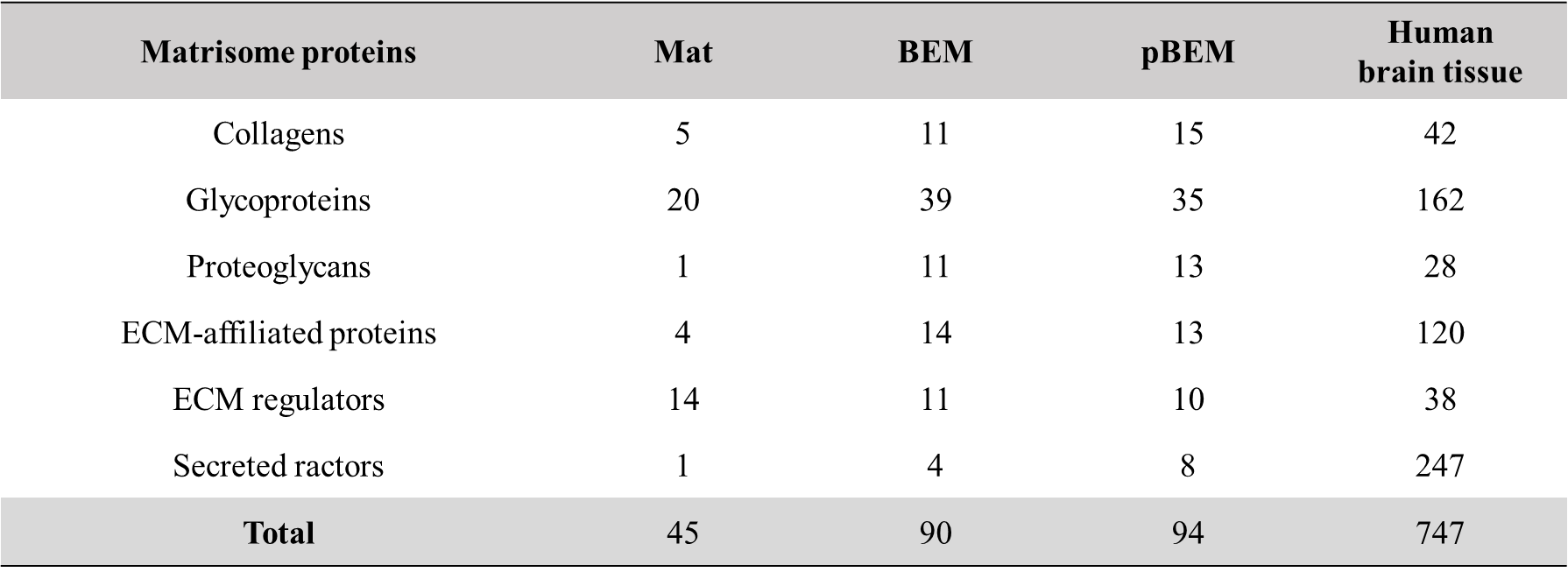
**

**Supplementary Table 4.** The top 30 biological process terms of the gene ontology enrichment analysis of all the proteins identified in BEM. For the statistical test, Fisher’s exact test was used with false discovery rate (FDR) correction.


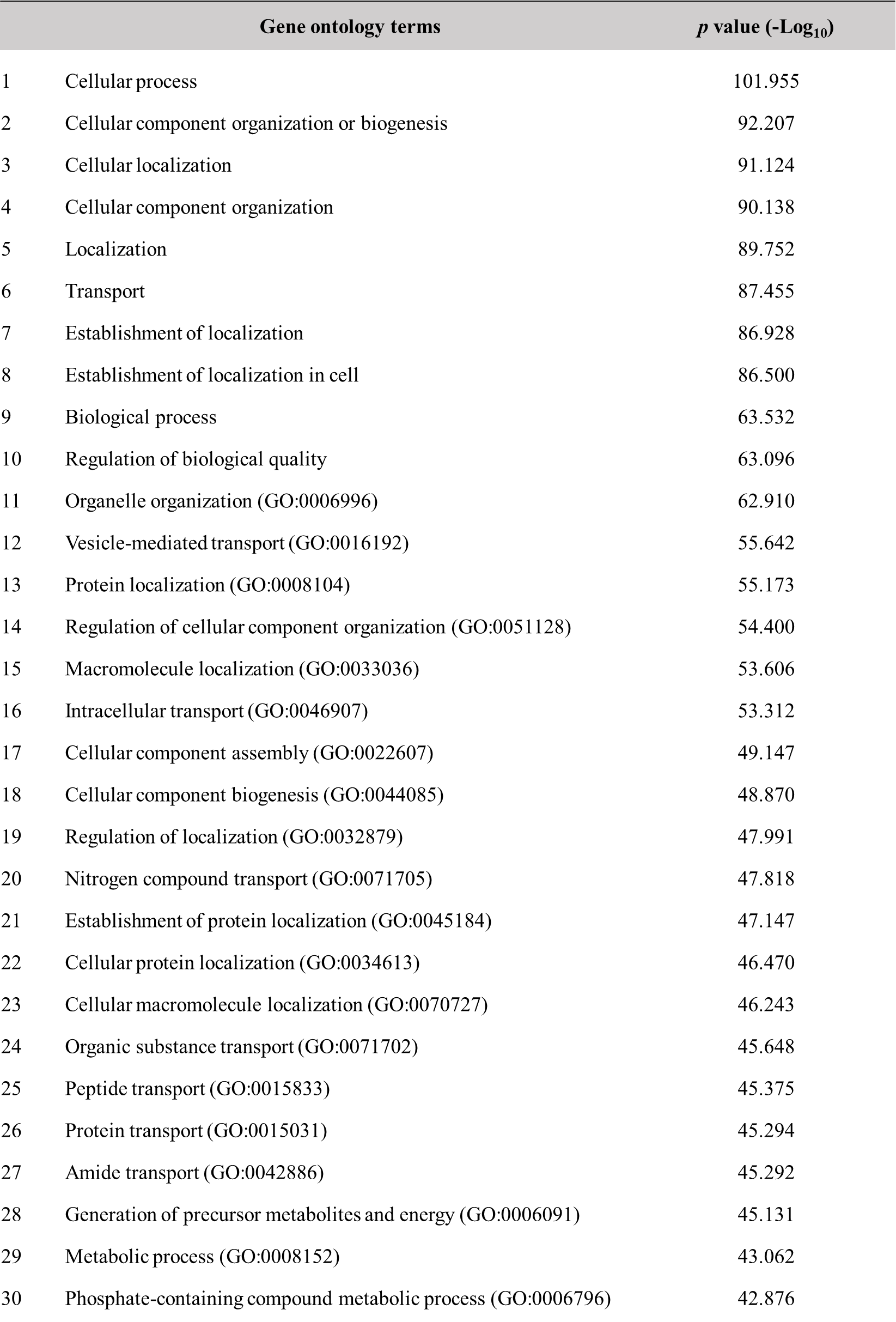


**Supplementary Table 5.** Simulation parameters used to predict the glucose diffusion profiles in the microfluidic device.

| **Parameter** | **Value** | **Ref.** |
| --- | --- | --- |
| Density of culture medium (kg/m^3^) | 1030 | [^2^](#_ENREF_2) |
| Dynamic viscosity of culture medium (Pa⋅s) | 0.0025 | [^2^](#_ENREF_2) |
| Glucose diffusion coefficient in medium (cm^2^/s) | 9.25 × 10^-6^ | [^3^](#_ENREF_3) |
| Glucose diffusion coefficient in organoid (cm^2^/s) | 5.5 × 10^-7^ | [^4^](#_ENREF_4) |
| Glucose concentration in culture medium (mM) | 21.25 | Calculated from our data |
| Porosity of hydrogel | 0.9 | [^5^](#_ENREF_5) |
| Maximum glucose consumption (mol/cell⋅s) | 3.9 × 10^-17^ | [^6^](#_ENREF_6) |
| Michaelis-Menten constant *k_m_* of glucose (mM) | 4 × 10^-2^ | [^6^](#_ENREF_6) |

**Supplementary Table 6.** The information of antibodies used for immunostaining.

**
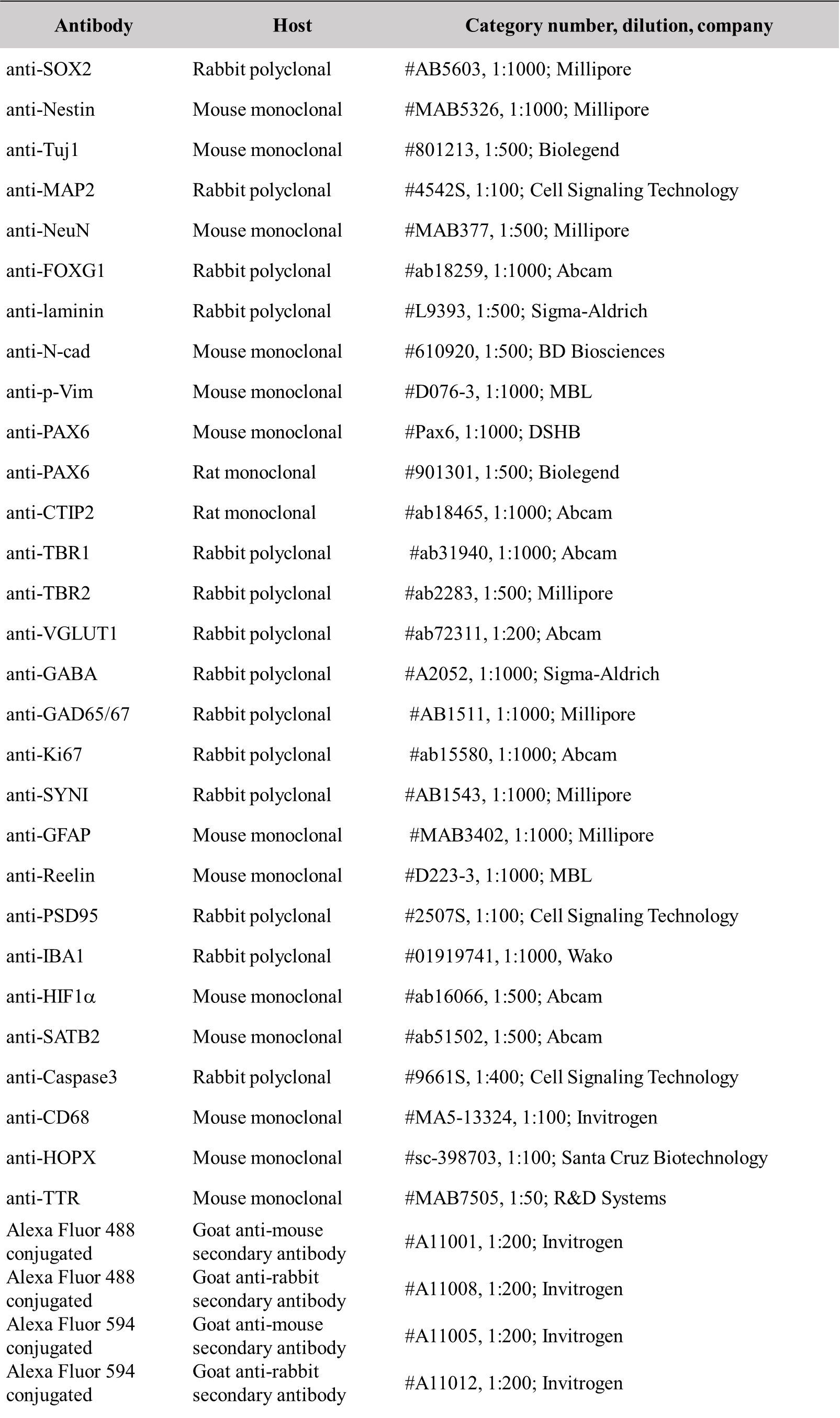
**

**Supplementary Table 7.** The information of Taqman primers used for qPCR.

**
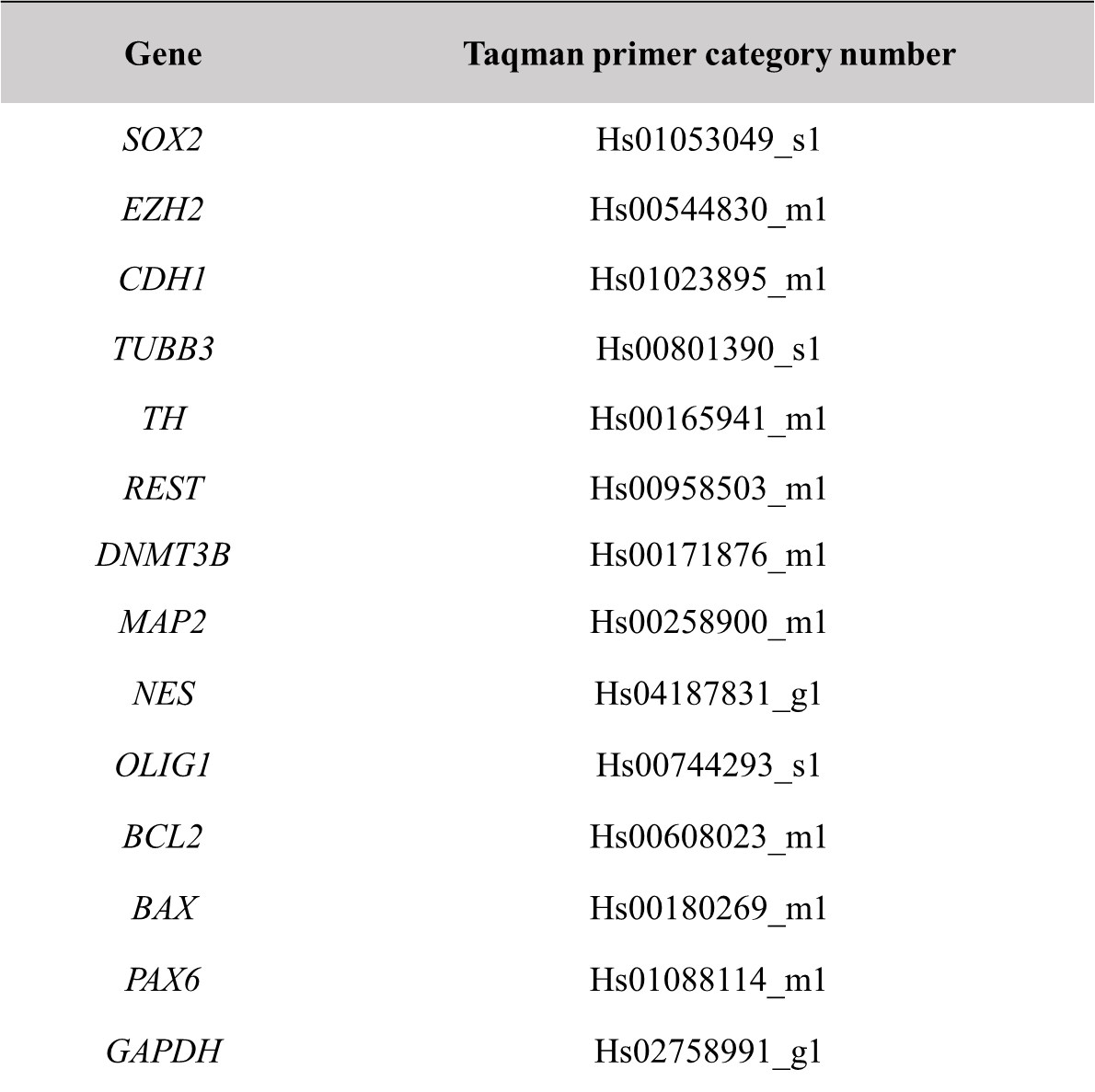
**

**References**

1. Uhlén M*, et al.* Tissue-based map of the human proteome. *Science* **347**, 1260419 (2015).

2. Singh H, Teoh SH, Low HT, Hutmacher DW. Flow modelling within a scaffold under the influence of uni-axial and bi-axial bioreactor rotation. *J. Biotechnol.* **119**, 181-196 (2005).

3. Li CK. The glucose distribution in 9L rat brain multicell tumor spheroids and its effect on cell necrosis. *Cancer* **50**, 2066-2073 (1982).

4. Casciari JJ, Sotirchos SV, Sutherland RM. Glucose diffusivity in multicellular tumor spheroids. *Cancer Res.* **48**, 3905-3909 (1988).

5. Fissell WH*, et al.* Solute partitioning and filtration by extracellular matrices. *Am. J. Physiol. Renal. Physiol.* **297**, F1092-F1100 (2009).

6. Rousset N, Monet F, Gervais T. Simulation-assisted design of microfluidic sample traps for optimal trapping and culture of non-adherent single cells, tissues, and spheroids. *Sci. Rep.* **7**, 245 (2017).
