# Supplementary material for: Microfluidic device with brain extracellular matrix promotes structural and functional maturation of human brain organoids
Source: Nat Commun. 2021 Aug 5;12:4730. doi: 10.1038/s41467-021-24775-5 (PMC8342542; doi:10.1038/s41467-021-24775-5)
Supplement: Supplementary file 2 — Description of Additional Supplementary Files [file 41467_2021_24775_MOESM2_ESM.docx]

**Description of Additional Supplementary Files**

Title: Supplementary Video 1.

Description: The nuclei-stained (Cyto16) organoid in the human brain extracellular matrix (BEM) in various zstacks (75-day sample) (scale bar = 200 μm).

Title: Supplementary Video 2.

Description: The human brain organoid cultured in the brain extracellular matrix (BEM) immunostained with neuronal markers (Tuj1, MAP2) at different z-stacks (30-day sample). Samples were cleared using the CUBIC protocol before immunostaining.

Title: Supplementary Video 3.

Description: Multi-well microfluidic device placed on the rocker showing fluid flow between wells.

Title: Supplementary Video 4.

Description: Real-time spontaneous changes of intracellular Ca2+ levels in Fluo-4 AM-loaded brain organoid cultured in human brain extracellular matrix (BEM) in the microfluidic device on day 75 (scale bar = 100 μm).
