# Supplementary material for: Microfluidic device with brain extracellular matrix promotes structural and functional maturation of human brain organoids
Source: Nat Commun. 2021 Aug 5;12:4730. doi: 10.1038/s41467-021-24775-5 (PMC8342542; doi:10.1038/s41467-021-24775-5)
Supplement: Supplementary file 7 — Reporting Summary [file 41467_2021_24775_MOESM7_ESM.pdf]

nature research

Corresponding author(s): Seung-Woo Cho

Last updated by author(s): Jun 25, 2021

Reporting Summary

Nature Research wishes to improve the reproducibility of the work that we publish. This form provides structure for consistency and transparency in reporting. For further information on Nature Research policies, see Authors & Referees and the Editorial Policy Checklist .

Statistics

For all statistical analyses, confirm that the following items are present in the figure legend, table legend, main text, or Methods section.

n/a	Confirmed	
☐	☒	The exact sample size (*n*) for each experimental group/condition, given as a discrete number and unit of measurement
☐	☒	A statement on whether measurements were taken from distinct samples or whether the same sample was measured repeatedly
☐	☒	The statistical test(s) used AND whether they are one- or two-sided<br>*Only common tests should be described solely by name; describe more complex techniques in the Methods section.*
☐	☒	A description of all covariates tested
☐	☒	A description of any assumptions or corrections, such as tests of normality and adjustment for multiple comparisons
☐	☒	A full description of the statistical parameters including central tendency (e.g. means) or other basic estimates (e.g. regression coefficient) AND variation (e.g. standard deviation) or associated estimates of uncertainty (e.g. confidence intervals)
☐	☒	For null hypothesis testing, the test statistic (e.g. *F*, *t*, *r*) with confidence intervals, effect sizes, degrees of freedom and *P* value noted<br>*Give P values as exact values whenever suitable.*
☒	☐	For Bayesian analysis, information on the choice of priors and Markov chain Monte Carlo settings
☒	☐	For hierarchical and complex designs, identification of the appropriate level for tests and full reporting of outcomes
☐	☒	Estimates of effect sizes (e.g. Cohen's *d*, Pearson's *r*), indicating how they were calculated

Our web collection on statistics for biologists contains articles on many of the points above.

Software and code

Policy information about availability of computer code

Data collection	Zeiss ZEN Microscope Software (black edition), Infinite M200 Pro, Step One Plus Real-Time PCR system, MultiClamp 700B amplifier, Digidata 1322A analog-to-digital board, HISEQ 2500 sequencing system, Tophat (v2.0.13), Cuffdiff (v2.2.0), Xcaliber software (v3.1), Proteome discoverer 2.2
Data analysis	Graphpad Prism (v8), CummeRbund (v2.8.2), Cufflinks (v1.3.0), DAVID program (v6.8), Broad Institute GSEA software (v3.0), COMSOL Multiphysics software (v5.3)

For manuscripts utilizing custom algorithms or software that are central to the research but not yet described in published literature, software must be made available to editors/reviewers. We strongly encourage code deposition in a community repository (e.g. GitHub). See the Nature Research guidelines for submitting code & software for further information.

Data

Policy information about availability of data

All manuscripts must include a data availability statement. This statement should provide the following information, where applicable:

- Accession codes, unique identifiers, or web links for publicly available datasets
- A list of figures that have associated raw data
- A description of any restrictions on data availability

The mass spectrometry proteomics data generated in this study have been deposited in the ProteomeXchange Consortium via the PRIDE partner repository with the dataset identifier PXD025397 [http://proteomecentral.proteomexchange.org/cgi/GetDataset?ID=PXD025397]. The RNA sequencing data generated in this study are available in the NCBI Gene Expression Omnibus (GEO) under accession code GSE145386 [https://www.ncbi.nlm.nih.gov/geo/query/acc.cgi?acc=GSE145386]. Source data are provided with this paper. Raw data for all figures and p values for supplementary figures are provided in Source Data file. Lists of total proteins and proteins that are known to be enriched in human brain tissues in all samples identified by proteomic analysis are also provided in Source Data file. The reference datasets of brain regions used for comparison are available in the Allen BrainSpan human transcriptome data set [www.brainspan.org/static/download.html]. The protein samples were identified by searching MS and MS/MS data of peptides against the homo sapiens UniProt database (2020.10 release) for human BEM, Sus scrofa UniProt database (2020.12 release) for pBEM, and Mus musculus UniProt database (2020.12 release) for Mat. Proteins identified in Mat and human BEM were

compared with the datasets in the Human Protein Atlas portal [www.proteinatlas.org]. The contents and composition of matrisome proteins in human brain tissues were analyzed from the data in the Human Proteome Map [www.humanproteomemap.org]. All microscopic image datasets used to make the figures and for quantification are too large and numerous to be publicly shared, yet they are available for research purposes from the corresponding author on reasonable request.

Field-specific reporting

Please select the one below that is the best fit for your research. If you are not sure, read the appropriate sections before making your selection.

[x] Life sciences   [ ] Behavioural & social sciences   [ ] Ecological, evolutionary & environmental sciences

For a reference copy of the document with all sections, see nature.com/documents/nr-reporting-summary-flat.pdf

Life sciences study design

All studies must disclose on these points even when the disclosure is negative.

Sample size	We chose the sample size based on our preliminary studies and in accordance to the standards in the field. We performed at least 3 biological replicates over 1 to 7 independent experiments. For image-based quantification and gene expression analysis, we performed at least 3 to 6 biological samples. For RNA-sequencing, three biological replicates were analyzed.
Data exclusions	For the quality control, when majority of Mat organoids (control) were suboptimally formed and showed low survival rate [Lancaster, M.A. & Knoblich, J.A. Generation of cerebral organoids from human pluripotent stem cells. Nat. Protoc. 9, 2329–2340 (2014)], the organoid samples of all groups in the same experimental batches were not analyzed. If not, all samples were analyzed in order to obtain the full spectrum of variability of Mat and BEM organoids [Lancaster MA, et al. Guided self-organization and cortical plate formation in human brain organoids. Nat. Biotechnol. 35, 659-666 (2017)].
Replication	The key findings in the study were reliably reproduced in several independent experiments, except for the neuronal spontaneous postsynaptic current measurement study, where multiple samples were measured within single independent experiment. All attempts at replication were successful.
Randomization	The embryoid bodies (EBs) were randomized to be encapsulated into 3D hydrogel for the generation of brain organoids. Mat and BEM organoids were randomly selected for each type of experiment.
Blinding	The investigators who performed the proteomics, RNA-sequencing, 3D imaging, and electrophysiology were blinded to the genotypes of the samples. During the quantification of the immunostainings, and analysis of RNA-sequencing and electrophysiological properties, the investigators were blinded.

Reporting for specific materials, systems and methods

We require information from authors about some types of materials, experimental systems and methods used in many studies. Here, indicate whether each material, system or method listed is relevant to your study. If you are not sure if a list item applies to your research, read the appropriate section before selecting a response.

Materials & experimental systems

n/a	Involved in the study	
[ ]	[x]	Antibodies
[ ]	[x]	Eukaryotic cell lines
[x]	[ ]	Palaeontology
[x]	[ ]	Animals and other organisms
[ ]	[x]	Human research participants
[x]	[ ]	Clinical data

Methods

n/a	Involved in the study	
[x]	[ ]	ChIP-seq
[x]	[ ]	Flow cytometry
[x]	[ ]	MRI-based neuroimaging

Antibodies

Antibodies used

anti-SOX2 Rabbit polyclonal #AB5603, 1:1000; Millipore
anti-Nestin Mouse monoclonal #MAB5326, 1:1000; Millipore
anti-Tuj1 Mouse monoclonal #801213, 1:500; Biolegend
anti-MAP2 Rabbit polyclonal #4542S, 1:100; Cell Signaling Technology
anti-NeuN Mouse monoclonal #MAB377, 1:500; Millipore
anti-FOXG1 Rabbit polyclonal #ab18259, 1:1000; Abcam
anti-laminin Rabbit polyclonal #L9393, 1:500; Sigma-Aldrich
anti-N-cad Mouse monoclonal #610920, 1:500; BD Biosciences
anti-p-Vim Mouse monoclonal #D076-3, 1:1000; MBL
anti-PAX6 Mouse monoclonal #Pax6, 1:1000; DSHB

anti-PAX6 Rat monoclonal #901301, 1:500; Biolegend
anti-CTIP2 Rat monoclonal #ab18465, 1:1000; Abcam
anti-TBR1 Rabbit polyclonal #ab31940, 1:1000; Abcam
anti-TBR2 Rabbit polyclonal #ab2283, 1:500; Millipore
anti-VGLUT1 Rabbit polyclonal #ab72311, 1:200; Abcam
anti-GABA Rabbit polyclonal #A2052, 1:1000; Sigma-Aldrich
anti-GAD65/67 Rabbit polyclonal #AB1511, 1:1000; Millipore
anti-Ki67 Rabbit polyclonal #ab15580, 1:1000; Abcam
anti-SYNI Rabbit polyclonal #AB1543, 1:1000; Millipore
anti-GFAP Mouse monoclonal #MAB3402, 1:1000; Millipore
anti-Reelin Mouse monoclonal #D223-3, 1:1000; MBL
anti-PSD95 Rabbit polyclonal #2507S, 1:100; Cell Signaling Technology
anti-IBA1 Rabbit polyclonal #01919741, 1:1000, Wako
anti-HIF1alpha Mouse monoclonal #ab16066, 1:500; Abcam
anti-SATB2 Mouse monoclonal #ab51502, 1:500; Abcam
anti-Caspase3 Rabbit polyclonal #9661S, 1:400; Cell Signaling Technology
anti-CD68 Mouse monoclonal #MA5-13324, 1:100; Invitrogen
anti-HOPX Mouse monoclonal #sc-398703, 1:100; Santa Cruz Biotechnology
anti-TTR Mouse monoclonal #MAB7505, 1:50; R&D Systems
Alexa Fluor 488 goat anti-mouse secondary antibody #A11001, 1:200; Invitrogen
Alexa Fluor 488 goat anti-rabbit secondary antibody #A11008, 1:200; Invitrogen
Alexa Fluor 594 goat anti-mouse secondary antibody #A11005, 1:200; Invitrogen
Alexa Fluor 594 goat anti-rabbit secondary antibody #A11012, 1:200; Invitrogen

Validation

The antibodies used in this work have been used in previously published reports and/or validated from the companies where we purchased the antibodies.

Anti-SOX2 (Millipore, #AB5603) reacts with human and mouse, and has been published and validated for use in immunohistochemistry, as stated on the Millipore website.
Anti-Nestin (Millipore, #MAB5326) reacts with human, and has been published and validated for use in immunohistochemistry, as stated on the Millipore website.
Anti-Tuj1 (Biolegend, #801213) reacts with human, mouse, and rat, and has been published and validated for use in immunohistochemistry, as stated on the Biolegend product sheet.
Anti-MAP2 (Cell Signaling Technology, #4542S) reacts with human, mouse, rat, and monkey, and has been published and validated for use in immunohistochemistry, as stated on the Cell Signaling Technology product page.
Anti-NeuN (Millipore, #MAB377) reacts with human, salamander, pig, chicken, mouse, ferret, avian, and rat, and has been published and validated for use in immunohistochemistry, as stated on the Millipore website.
Anti-FOXG1 (Abcam, #ab18259) reacts with human, rat, and mouse, and has been published and validated for use in immunohistochemistry, as stated on the Abcam website.
Anti-laminin (Sigma-Aldrich, #L9393) reacts with human, and has been published and validated for use in immunohistochemistry, as stated on the Sigma-Aldrich website.
Anti-N-cadherin (BD Biosciences, #610920) reacts with human, rat, mouse, and chicken, and has been validated for use in immunohistochemistry, as stated on the BD Biosciences website.
Anti-p-Vimentin (MBL, #D076-3) reacts with human, rat, and mouse, and has been published and validated for use in immunohistochemistry, as stated on the MBL website.
Anti-PAX6 (DSHB, #Pax6) reacts with human and various animals and has been published and validated for use in immunohistochemistry, as stated on the DSHB data sheet online.
Anti-PAX6 (Biolegend, #901301) reacts with human, rat, and mouse, and has been published and validated for use in immunohistochemistry, as stated on the Biolegend website.
Anti-CTIP2 (Abcam, #ab18465) reacts with human and mouse, and has been published and validated for use in immunohistochemistry, as stated on the Abcam website.
Anti-TBR1 (Abcam, #ab31940) reacts with human, rat, and mouse, and has been published and validated for use in immunohistochemistry, as stated on the Abcam website.
Anti-TBR2 (Millipore, #ab2283) reacts with human and mouse, and has been published and validated for use in immunohistochemistry, as stated on the Millipore website.
Anti-VGLUT1 (Abcam, #ab72311) reacts with human and rat, and has been published and validated for use in immunohistochemistry, as stated on the Abcam product sheet.
Anti-GABA (Sigma-Aldrich, #A2052) reacts with wide range of species, and has been published and validated for use in immunohistochemistry, as stated on the Sigma-Aldrich website.
Anti-GAD65/67 (Millipore, #AB1511) reacts with human, rat, mouse, and cat, and has been published and validated for use in immunohistochemistry, as stated on the Millipore website.
Anti-Ki67 (Abcam, #ab15580) reacts with human and mouse, and has been published and validated for use in immunohistochemistry, as stated on the Abcam website.
Anti-SYNI (Millipore, #AB1543) reacts with human, rat, mouse, and bovine, and has been published and validated for use in immunohistochemistry, as stated on the Millipore website.
Anti-GFAP (Millipore, #MAB3402) reacts with human, rat, mouse, pig, chicken, bovine, and rabbit, and has been published and validated for use in immunohistochemistry, as stated on the Millipore website.
Anti-Reelin (MBL, #D223-3) has been validated to react with mouse, and for use in immunohistochemistry, as stated on the MBL

website. Several published papers have used this antibody for immunostaining with human-derived samples [e.g. Ogawa et al., Glioblastoma model using human cerebral organoids. Cell Rep. 23, 1220–1229 (2018); Yoon et al., Reliability of human 3D cortical organoid generation. Nat. Methods 16, 75–78 (2019); Eze et al., Single-cell atlas of early human brain development highlights heterogeneity of human neuroepithelial cells and early radial glia. Nat. Neuroci. 24, 584–594 (2021)].

Anti-PSD95 (Cell Signaling Technology, #2507S) reacts with human, rat, and mouse, and has been validated for use in West blotting and immunoprecipitation, as stated on the Cell Signaling Technology website. Several published papers have used this antibody for immunostaining analysis [e.g. Okubo et al., Treatment with a gamma-secretase inhibitor promotes functional recovery in human iPSC-derived transplants for chronic spinal cord injury. Stem Cell Rep. 11, 1416–1432 (2018); Gupta et al., Fibroblast growth factor 2 regulates activity and gene expression of human post-mitotic excitatory neurons. J. Neurochem. 145, 188–203 (2017)].

Anti-IBA1 (Wako, #01919741) reacts with human, rat, and mouse, and has been validated for use in immunohistochemistry, as stated on the Wako website.

Anti-HIF1alpha (Abcam, #ab16066) reacts with human, and has been published and validated for use in immunohistochemistry, as stated on the Abcam website.

Anti-SATB2 (Abcam, #ab51502) reacts with mouse and is predicted to react with human and rat. Several published papers have used this antibody for immunostaining with human-derived samples [e.g. Giandomenico et al., Cerebral organoids at the air-liquid interface generate diverse nerve tracts with functional output. Nat. Neurosci. 22, 669–679 (2019); Yoon et al., Reliability of human 3D cortical organoid generation. Nat. Methods 16, 75–78 (2019)]. The antibody has been validated for use in immunohistochemistry, as stated on the Abcam website.

Anti-CD68 (Invitrogen, #MA5-13324) reacts with human, rat, mouse, hamster, pig, rabbit, and rhesus monkey, and has been published and validated for use in immunohistochemistry, as stated on the Invitrogen website.

Anti-HOPX (Santa Cruz Biotechnology, #sc-398703) reacts with human, rat, and mouse, and has been published and validated for use in immunohistochemistry, as stated on the Santa Cruz Biotechnology website.

Anti-TTR (R&D Systems, #MAB7505) reacts with human, and has been validated for use in immunohistochemistry, as stated on the R&D Systems website.

Eukaryotic cell lines

Policy information about cell lines

Cell line source(s)	Human induced pluripotent stem cell (iPSC) lines WT3 (available upon request, Yonsei Stem Cell Research Center, Yonsei University College of Medicine) and KYOU-DXR0109B (#ACS-1023, ATCC) were used for generation of brain organoids.
Authentication	Human iPSCs were authenticated through immunostaining against several pluripotency markers, including OCT4, Sox2, and Tra-1-60.
Mycoplasma contamination	Human iPSCs were tested for mycoplasma contamination and confirmed to be free from mycoplasma contamination.
Commonly misidentified lines (See ICLAC register)	No commonly misidentified cell lines were used.

Human research participants

Policy information about studies involving human research participants

Population characteristics	We predicted that the ECM proteins may vary between the regions of the brain, and thus temporal lobe tissues from patients with epilepsy were used.
Recruitment	Patients who received surgical treatment at temporal lobe had a choice whether to participate or not. Samples were collected with no bias.
Ethics oversight	The study protocol was approved by Yonsei University College of Medicine. Full information on the approval of the study protocol is provided in the manuscript.

Note that full information on the approval of the study protocol must also be provided in the manuscript.
